# Time trends of comparative self-rated health in adults aged 25-34 in the Northern Sweden MONICA study, 1990-2014

**DOI:** 10.1371/journal.pone.0187896

**Published:** 2017-11-20

**Authors:** Mattias Waller Lidström, Patrik Wennberg, Robert Lundqvist, Annika Forssén, Göran Waller

**Affiliations:** 1 Department of Public Health and Clinical Medicine, Division of Family Medicine, Umeå University, Umeå, Sweden; 2 Research Unit, County Council of Norrbotten, Luleå, Sweden; Leibniz Institute for Prvention Research and Epidemiology BIPS, GERMANY

## Abstract

Self-rated health (SRH) accounts comprehensively for many health domains. The aim of this paper was to investigate time trends and associations between age-comparative self-rated health and some known determinants in a general population aged 24–34 years. Population-based cross-sectional surveys were performed in 1990, 1994, 1999, 2004, 2009 and 2014 in Northern Sweden. Out of 3500 invited persons, 1811 responded. Comparative SRH was measured on a three-grade ordinal scale by the question: “How would you assess your general health condition compared to persons of your own age?” with the alternatives “better/worse/similar”. Over the period 1990 to 2014, the percentage of women rating comparative SRH as “worse” increased steadily, from 8.5% in 1990 reaching 20% in 2014 (p for trend 0.007). Among men, this pattern was almost the opposite, with increasing proportions rating “better” (p for trend <0.000). Time trends for physical activity in leisure time; length of education; Body Mass Index; anxiety; depressive emotions and satisfaction with economy showed a similar pattern for men and women. Factors that might contribute to the development of time trends for comparative SRH are discussed.

## Introduction

Self-rated health (SRH) is a widespread method of assessing health in populations and is an independent predictor of future morbidity, such as myocardial infarction, risk of diabetes, depression, rheumatic disease and sick leave; medical care utilisation; and mortality [1 2 3]. Two principal variants of SRH questions are often used: the general “How would you rate your health at present time?” and the comparative “How would you rate your health in comparison to others of the same age?” [4]. These are answered on a five- or three-level categorical scale. General and comparative SRH have similar associations with outcomes in cohort studies and with determinants in cross-sectional studies, but reflect different aspects of health and are not equivalent [4 5]. The comparative question contains in itself a reference for evaluations (compared to others) and steers the comparison to real persons and might thus be considered semantically clearer [6].

Gender issues are important in public health research. Gender is defined by the World Health Organization: “Sex refers to the biological and physiological characteristics that define men and women. Gender refers to the socially constructed roles, behaviours, activities, and attributes that a given society considers appropriate for men and women” [7]. Sweden is regarded as a country with a high level of gender equality in international comparison, and gender equality is an undisputable societal norm [8]. Still gender continues to play a considerable part in how work, wealth, power, and time use are distributed in the Swedish society [9]. Women in Sweden are gainfully employed to the same degree as men [9]. The Swedish labour market is however, gender-segregated; a large number of women work in the public sector, in caring and service professions, while men dominate in the private sector and work in areas such as construction and transportation [9]. Women earn less than men, despite being higher educated [9]. Women spend almost twice as much time as men performing household chores, and to a large extent shoulder the main responsibility for their families, including invisible tasks such as planning for everyday life to run smoothly, in addition to the caring of children and ailing or sick relatives [9 10]. Furthermore, women continue to be exposed to sexual, physical and psychological violence from their partners and former partners to a much higher degree than men [11].

International comparisons on self-reported items of health in several countries have been made based on WHO world health survey data [12 13]. Gendered differences in health could be seen in pooled data from all countries with women reporting worse health than men. Social determinants, mainly employment and education accounted for much of the differences although some of the differences were unexplained and presumably due to factors not in the model.

The situation in Sweden shows the same overall picture, women rating worse health than men, women having higher rates of sick listing particularly long term sick listing [14 15]. Johansson et al. investigated time trends of SRH from 1980 to 2005 in Sweden and found that SRH became poorer or was unchanged in those aged 16–47 but got better among person aged 48 or older [16]. Time trend for the period 1997–2006 indicated a highly prevalent, mental ill-health among the young in Stockholm County, a region representative of urbanized Western societies [17]. Swedish women in general and young women in particular report higher rates of anxiety and depression than men, both associated with poor SRH [9 14]. Concern has been raised over the increasing levels of anxiety and depressive emotions among young adults [17 18]. As we previously had done some work on comparative self-rated health and had access to a database from the Northern Sweden Monica study with time trends among young adults for comparative SRH and factors known to be associated with SRH we set out to investigate the issue. To the best of our knowledge, there are no studies using comparative self-rated health as an outcome variable among young adults in the Swedish setting. Several factors are associated with SRH. Among these we chose to investigate time trends of physical activity in leisure time; educational level; Body Mass Index (BMI); depressive feelings; anxiety; and satisfaction with economy. The rationale for this lies in the age group investigated where diseases on a population level play a minor role but habits reflected in BMI, physical activity, psychosocial factors, educational level, satisfaction with economy, feelings of depression and anxiety are factors that presumably play a greater role in SRH. These determinants have also been described in the Northern Sweden Monica study on a population basis for the ages 25–74 [14].

The aim of this article was to investigate time trends of comparative SRH over the period 1990 to 2014 in men and women aged 25–34 years. A secondary aim was to describe time trends of some determinants.

### Research questions

- Has comparative SRH of men and women aged 25 to 34 changed over the period 1990 to 2014 and if so, in what way?
- Have physical activity in leisure time, educational level, Body Mass Index, depressive feelings, anxiety and satisfaction with economy changed in the age group during the same period?
- Do associations between comparative SRH and physical activity in leisure time, educational level, Body Mass Index, depressive feelings, anxiety and satisfaction with economy show the same pattern for men and women?

## Material and methods

### The Northern Sweden MONICA study

The Northern Sweden MONICA study is a cross-sectional sequential study primarily focused on trends and determinants for cardiovascular disease and diabetes in the two northernmost counties in Sweden. Data were collected as independent, randomly selected population-based cross-sectional samples on six different occasions from 1990 to 2014 [19]. At the time of the 2014 survey, the number of participants cumulative for all years exceeded 12,000 individuals. All respondents between 25 and 34 years of age (944 women and 867 men) in the MONICA population between 1990 and 2014 formed the study group of this article. Both postal questionnaires and questionnaires filled in at visits at local health centres were used.

### Comparative self-rated health

Comparative SRH was measured by the question: “How would you rate your health compared to others the same age: better, the same or worse?”

### Determinants

Physical activity in leisure time was assessed with a six-grade categorical scale where respondents rated the intensity and frequency of physical activities the last year ranging from “hardly ever” to “intense, several times per week”. Answers one and two were classified as low physical activity in leisure time, three and four as intermediate and five and six as high physical activity. Length of education was assessed with a 7- or 10-grade categorical scale based on current and previous Swedish school systems. Nine years of schooling or less was considered as short education, 10–12 years of schooling as intermediate education and post-secondary education as long education. BMI was calculated from measurements of height and weight performed by trained personnel at local health centres as part of the MONICA study concept. Participants were also asked if in the last month they “often have felt uneasy, depressed or that the future seems hopeless” and “have felt nervousness, anxiety or unrest”, with response alternatives “yes” or “no”. Personal economy was assessed by asking “How satisfied are you with your current situation regarding economy?” and providing a 7-grade Likert scale ranging from “very bad” to “excellent, could not be better”. Answers 1–2 were grouped as discontent with personal economy, 3–5 as intermediate and 6–7 as content with personal economy. The determinants of anxiety, depressive feelings and personal economy were introduced in 1999. In the 2014 survey, the items regarding anxiety, depression, personal economy and physical activity were omitted or rephrased, making these variables impossible to compare. We described data for determinants from 1990 to 2014 and we used data from 1999 to 2009 for calculations of associations between determinants and comparative self-rated health.

### Data analyses

Data analyses and statistical calculations were performed using SPSS version 22. We used the χ^2^ test to assess whether variations over time in the distribution of categorical variables were statistically significant. We used Linear-by-Linear Associations to test for trends on a table with df = 1. Associations between determinants and comparative SRH were assessed in a logistic ordinal regression model. The model uses an ordinal scale of outcomes instead of only two possible outcomes as in logistic regression analysis. The odds ratio (OR) can be interpreted as a change in odds when moving to the next category in an independent category/factor (for instance from intermediate to short educational length). The reference value for the determinants is set as the anticipated most favourable situation. Initially, we performed a univariate regression to determine crude ORs for all proposed determinants and subsequently a multivariable regression. Analyses were done separately for men and women.

The Northern Sweden MONICA Study has been approved by the regional ethical committee of Umeå, Sweden

## Results

**Fig 1:**
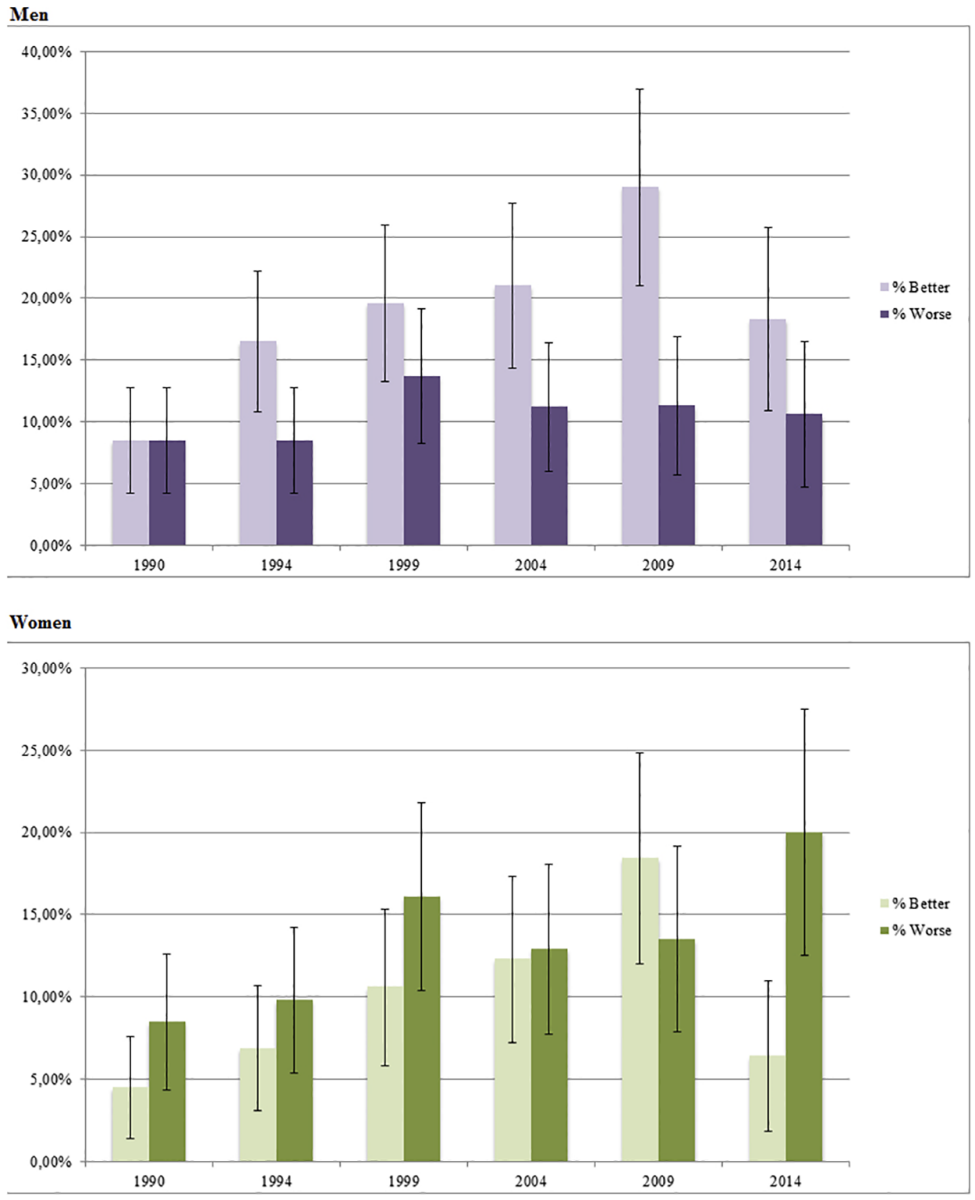
Time trends in proportions of "better" and "worse" self-rated health 1990–2014. Data from the Northern Sweden Monica Study 1990–2014.

The distribution of comparative self-rated health for men and women is presented in Fig 1. For men there was an increase of “worse” health, from 8.5% to 10.6% (p for trend 0.385), Men rating “better” rose from 8.5% to 18.3% (p for trend <0.000). For women, there was an increase in the proportions rating their health as “worse”, from 8.5% in 1990 to 20.0% in 2014 (p for trend 0.007). However when comparing the overall trends of health no statistical significant difference between men and women could be ascertained. Among women, the proportion rating “better” gradually increased from 4.5% in 1990 to 18.4% in 2009 and then fell to 6.4% in 2014 (p for trend 0.006).

**Fig 2:**
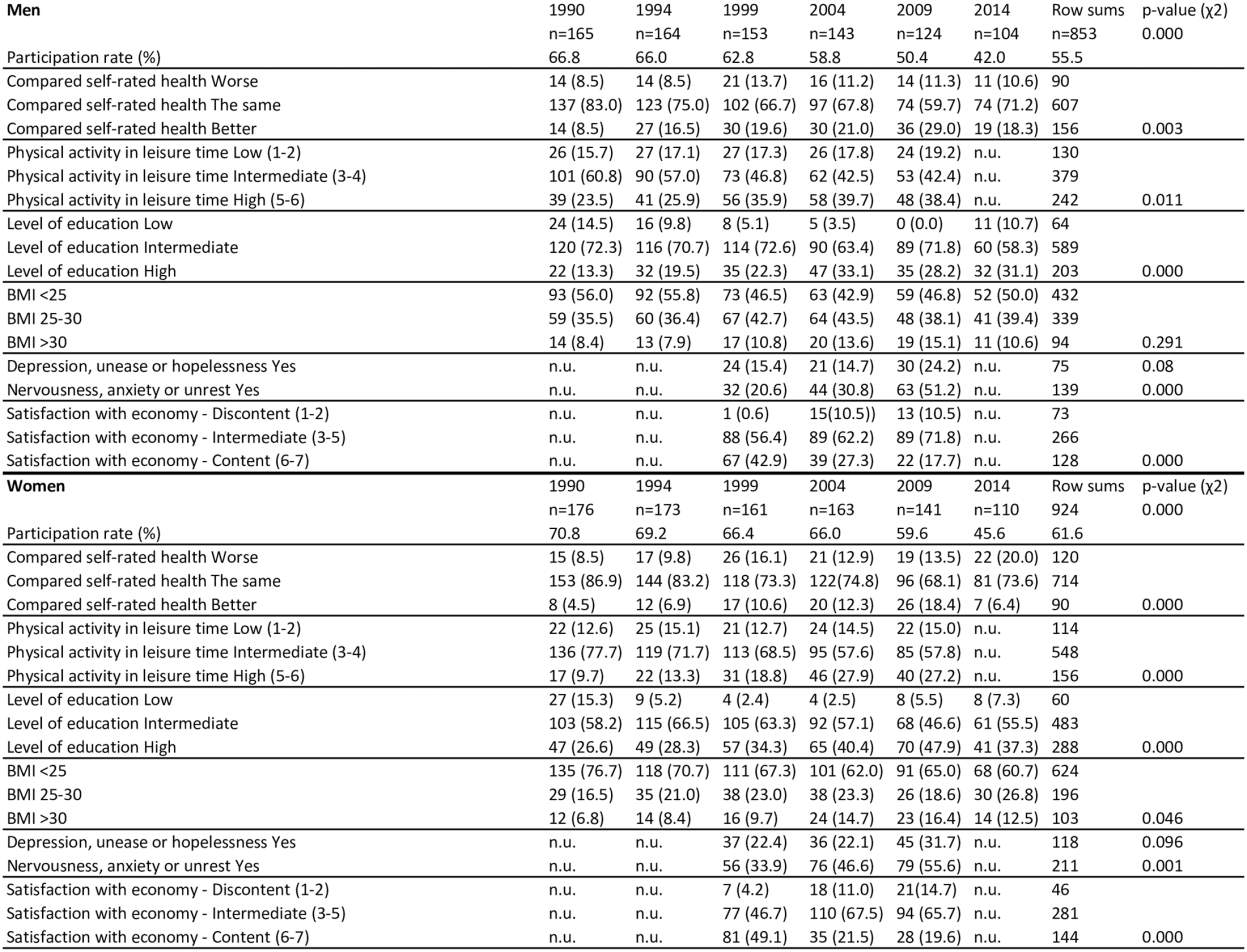
Time trends for self-rated health and determinants. Data from the Northern Sweden Monica Study 1990–2014.

Fig 2 shows absolute numbers and proportion of respondents in each category of comparative SRH and participation rates 1990 to 2014 and time trends for determinants from 1990 to 2014. There has been a downward trend of the participation rate for the survey since 1990; in 2014 the rate for the first time was below 50% in the selected age group.

Time trends for determinants in men and women showed that high physical activity in leisure time rose over time (p for trend men 0.011; women <0.000); as did BMI over 30 (p for trend men 0 08; women 0.005); nervousness, anxiety or unrest (p for trend men 0.001; women <0.000) and discontent with personal economy (p for trend men 0.001; women 0.002). For depression, the trend with increasing percentages answering “yes” was similar in both men and women but did not reach statistical significance.

**Fig 3:**
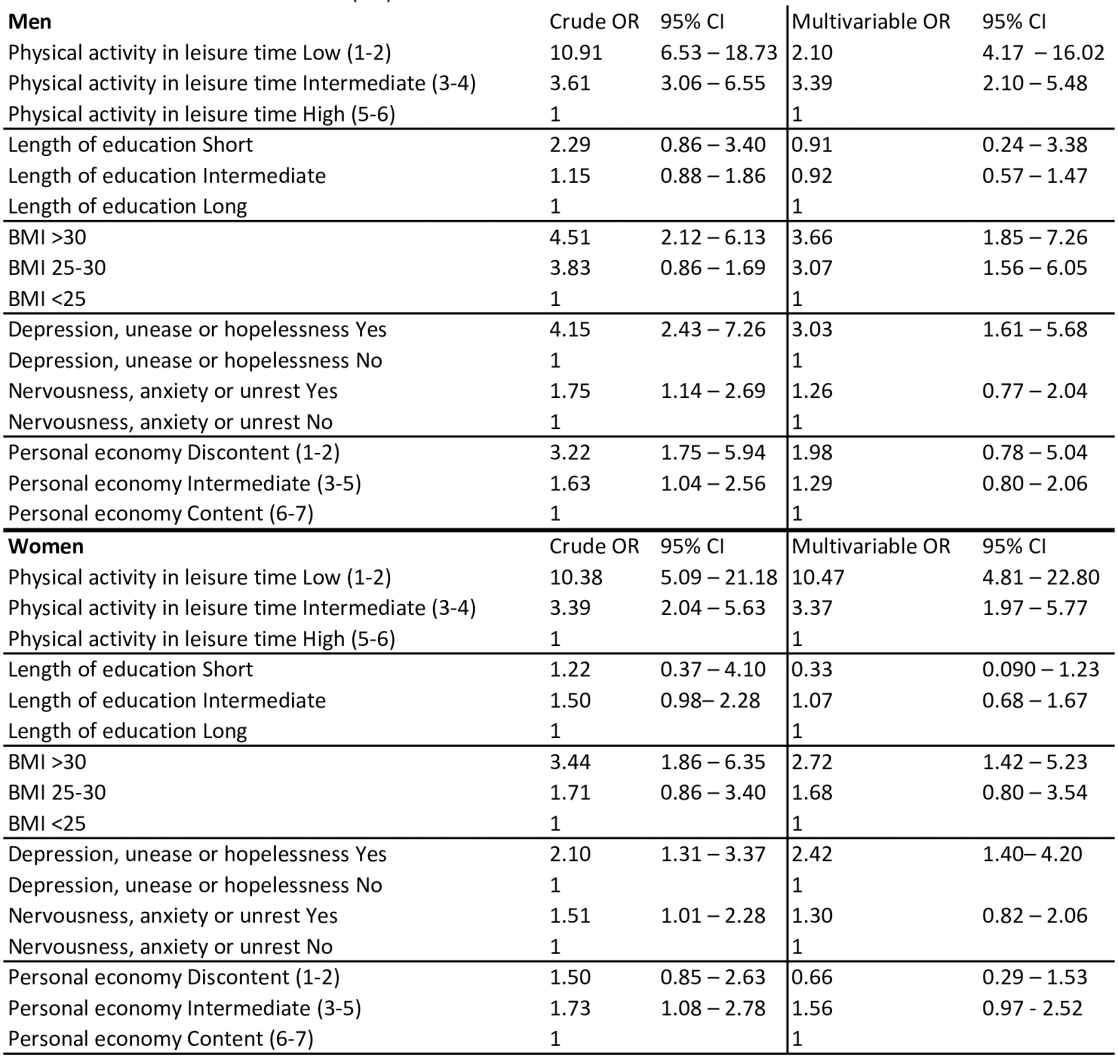
Ordinal regression analysis with compared self-rated health as dependent variable. Data from the Northern Sweden Monica Study 1990–2014.

The results of the ordinal logistic regression analysis are presented in Fig 3, showing associations between determinants and comparative SRH as odds ratios. In the multivariable analysis, adjusted for all variables, low and intermediate physical activity, BMI>30 and emotions of depression, unease or hopelessness retained their statistically significant associations with comparative SRH. The associations between variables and comparative SRH were similar for men and women.

## Discussion

### Principal findings

Over the period 1990 to 2014, the percentage of women rating comparative SRH as “worse” steadily increased, while the percentage rating “better” rose until 2009 and then fell in the 2014 survey, almost to the level of 1990. Among men, this pattern was almost the opposite, with increasing proportions rating “better”. There are thus different health trends for men and women but we could not ascertain a statistical significant difference in overall health ratings for men and women. Among both women and men time trends showed an increase in frequency of BMI >30; feelings of nervousness, anxiety or unrest and discontent with economy. Associations between SRH and determinants were similar for men and women in the period 1999 to 2009.

### Findings in relation to the literature

The trends of deteriorating SRH described in this study are in accordance with previous research in Australia [20]. Caution is however recommended by researchers in interpreting time trends in general self-rated health among young persons as four national surveys in the US showed striking discrepancies in time trends [21]. The Public Health Agency of Sweden uses the five-step general SRH question. Time trends from 1980 in Sweden show bad general health for men and women hovering between 0.9 and 4.4% without apparent time trend and only small differences between men and women [22]. The difference in time trends between these figures and ours could be due to our use of comparative SRH as it reflects the situation for a larger proportion of the population, around 10–20%, whereas only a few per cent report quite poor or poor health. Comparative SRH includes both a statement of health and a comparison and thus reflects other aspects of a subjective health evaluation. According to the status syndrome theory it can be argued that this comparison to others is important.[23]. Following the same theory, gender inequities and gendered norms are judged important to both the health and survival gap between women and men [24 8].

Our study shows deteriorating comparative SRH in young women despite Sweden being a strong welfare state. The rates of unemployed young adults compared to other countries are low. Large shares of young adults have post-secondary education and a high level of physical activity. This should make SRH better. Levels of nervousness, anxiety or unrest as well as discontent with personal economy have increased and this might worsen SRH. The changes over time in overall health could hardly be explained by increased prevalence of manifest disease in the young women in northern Sweden. Social changes not covered in our database have taken place in Sweden over the studied time periods. During the increased urbanisation in the last decades more women than men have moved to larger towns, often to study, which might affect the demographics of the sparsely populated area and SRH in those who stay. Similar to many other countries, downsizing and frequent reorganisations are performed in the public sector, such as health, elderly and social care [25 26] Such changes are related to burnout and stress of conscience in caregivers [26] and might be linked to the increased long-term sick-listing due to mental ill health among, particularly, young women (20–29 years) since 2010 [15]. The lack of equality in the private sphere usually becomes evident when a couple gets children, which usually happens in the studied ages [8]. Men and women perceive and report gender equality differently [8]. Women’s experience of gender inequality both at work and at home, create tiredness, tension and worry, as well as feelings of personal failure [10]. Women in Sweden also seem to worry more than men about other large collective questions, such as the climate threat [27]. To our understanding the differences in perceived equality must not be trivialized or disregarded.

Our previous work with SRH has taught us the importance of “subjectivity”. In ratings of SRH, differences do occur even between persons who seemingly share the same situation when it comes to sex education, income, social capital, wealth, medical health, functional level etcetera. Yet there can be a discrepancy in SRH. Difference in SRH is associated to hard medical outcomes such as disease and death [28 2] Causative biological factors are connected to the subjectivity of SRH [29]. Such links have been found in cytokines and physiological regulation (allostatic) of the human body [30 31]. The concept of allostatic load gives, by measuring psychophysiological stressors, a quantifiable measure of the pressure put on regulatory systems in the body striving to uphold equilibrium or homeostasis. The concept encompasses the concept of embodiment, how sociocultural and environmental influences translate to the human body) [32]. Researchers conclude: “It is becoming increasingly clear how subtle yet longstanding challenges impact on the human physiology and predispose to disease…likewise, it is acknowledged that it is subjective experience, not objectively quantifiable events, that becomes biologically inscribed.” [32].

It is thus necessary to also include not so “easily measured factors” trying to understand differences in SRH between men and women. Young adults in general and young women in particular suffer from expectations of being successful in school or work, being socially active and caring about one’s appearance [33]. A shift in the basis of a person’s value from intrinsic personal qualities and relations to external qualities and achievements such as success in school or at work, consumption patterns or activities in the leisure time is taking place today [34]. Self-esteem based on achievement is a risk for deteriorating health [34]. Furthermore, in upholding gender equality two norm systems conflict, the discourse of gender equality and the traditional family norm system [8]. The tension between these two systems puts a heavy burden on women trying to encompass both norm systems simultaneously.

Why men increasingly report better health is not readily explained. One possible explanation might be found in the development of more diversified norms for how to behave as a man, how to express oneself and what is expected by men thus putting less pressure on men and more lenient gendered norms. It has been argued that this gender convergence also might lead to health convergence for both men and women when it comes to well defined health outcomes [8 35]. In Sweden, men are leading the trend to less frequent smoking and the trend for parental leave is rising, although very slowly for men [36 37]. The use of internet might also give access to a wider array of male norms compared to being confined to models available in local social life e.g. small towns, rural areas or in isolated social groups.

Regarding both women and men, attention has been drawn to the situation of broken prospects and difficulties for young people in establishing themselves in adult life, such as achieving a job and finding housing [38]. Experiences of overwhelming stress while entering work life could be devastating.

Young persons have to orient among all these factors and forces, norms and expectations, stressors and conflicts. Making meaning of ones’ existence is a never ending human endeavour and entering adulthood forces this process of orientation and meaning making upon people. The process of existential meaning making is of great importance for health [39]. The term existential meaning making encapsulates what resources, strategies, and ideas/notions, individuals use to face the challenges of life, and to create meaning in one’s life. Attempts have been made to define the concept operationally, among others by the World Health Organization [39 40].

We propose that the issue of meaning making and subjectivity are included and expanded on, in a gender sensitive way, in efforts trying to understand the different health trends for men and women described by our data. The way to grasp these matters, we believe, is by qualitative research, systematically approaching and understanding stress, health and the effect of social constructs.

### Strengths and limitations

The main strength of this study is the time-trend perspective from 1990 to 2014 and the population-based methodology of the Northern Sweden MONICA Study. The decreasing participation rate is a limitation. A comparison of participants and non-participants conducted in 2009 for the Northern Sweden MONICA Study found that non-participants were younger, more likely to be smokers or to have diabetes and less likely to have a university education [41]. Non-participation is a matter of concern in research [42]. The effects of the dropout, however, are not entirely clear. We have not found arguments in the literature to claim that the dropouts are better off than participants, rather the contrary. [43 44]. This might mean that the trends towards larger proportions of “worse” health ratings in this material are underestimated. Another limitation is the fact that not all determinants could be followed for the entire analysed time period.

The questions on anxiety and depressive emotions were not used to indicate psychiatric disease but are self-reported emotions. The questions are not formally validated but used on their face value. The questions have, however, been used for many years by the Public Health Agency of Sweden.

The generalisability of our findings can be discussed. Time trends of comparative SRH among young adults should be followed in other settings. This might give further information on the appropriateness of using comparative SRH to follow time trends of health.

## Conclusion

Time-trends of comparative self rated health 1990–2014 in the Northern Sweden MONICA Study show an increase of women rating their health as worse whereas men increased the proportion rating better. Physical activity in leisure time, educational level, BMI, depressive feelings, anxiety and dissatisfaction with economy have also increased during the period. Time trends for these variables show the same pattern for men and women. Our findings point to that gender aspects of SRH need to be further explored.

## Supporting information

S1 Text Questionnaire 1990. (PDF)

S2 Text Questionnaire 1994. (PDF)

S3 Text Questionnaire 1999. (PDF)

S4 Text Questionnaire 2004. (PDF)

S5 Text Questionnaire 2009. (PDF)

S6 Text Questionnaire 2014. (PDF)

S7 Text Code book variables. (DOCX)
