# Supplementary material for: Time trends of comparative self-rated health in adults aged 25-34 in the Northern Sweden MONICA study, 1990-2014
Source: PLoS One. 2017 Nov 20;12(11):e0187896. doi: 10.1371/journal.pone.0187896 (PMC5695772; doi:10.1371/journal.pone.0187896)
Supplement: S1 Text — (PDF) [file pone.0187896.s001.pdf]

monica centre
WHO/OMS
northern sweden

Plats för etikett

VÄRLDSHÄLSOORGANISATIONENS KAMPANJ MOT HJÄRT-/KÄRLSJUKDOMAR

FRÅGEFORMULÄR DEL 1

INFORMATION

Det frågeformulär som vi nu ber dig besvara, innehåller frågor rörande bl a hälsa, arbete, fysisk aktivitet, rök- och motionsvanor. Alla uppgifter, som du lämnar, kommer att behandlas konfidentiellt .
Resultaten kommer att ställas samman på sådant sätt att det inte går att identifiera enskilda individer.

För att undersökningen skall ge ett rättvisande resultat är det viktigt att alla besvarar alla frågor. Varje person, som ej besvarar formulären, minskar undersökningens tillförlitlighet.

Om det är något du funderar på angående frågeformulären eller undersökningen, kontakta gärna någon av oss. Du når oss säkrast på telefon på förmiddagarna.

Tack på förhand för din vänliga medverkan!

Birgitta Stegmayr
MONICA-sekretariatet
Medicinska kliniken
Regionsjukhuset
901 85 UMEÅ
tel 090 - 10 25 18

Vivan Lundberg
MONICA-sekretariatet
Medicinska kliniken
Kalix lasarett
952 01 KALIX
tel 0923 - 131 33

Endast ett X på varje fråga där inte annat anges

Frågor rörande CIVILSTÅND och BOENDE

1. Vilket är ditt nuvarande civilstånd?

(1) I__I = ogift
(2) I__I = gift eller sammanboende
(3) I__I = skild eller separerad
(4) I__I = änka/änkling
(5) I__I = annat______________________________

2. Vilka personer sammanbor du med ? *(Flera alternativ kan vara aktuella, sätt kryss där det är lämpligt)*

(1) I__I = vuxen (make, maka, sambo)
(2) I__I = barn, ange antal I__I__I
(3) I__I = syskon, ange antal I__I__I
(4) I__I = mor
(5) I__I = far
(6) I__I = annan
(7) I__I = bor helt ensam

3. Var bor du?

(1) I__I = större tätort - Umeå, Skellefteå, Luleå, Boden, Piteå, Kiruna-orter med mer än 15 000 invånare
(2) I__I = annan tätort med mer än 1 000 invånare
(3) I__I = ort med mindre än 1 000 invånare

4. I vilket land är du född?

(1) I__I = Sverige
(2) I__I = Finland
(3) I__I = annat land

5. Vilken är den högsta utbildningsnivå som du har avslutat?

(1) I__I = folkskola
(2) I__I = grundskola
(3) I__I = folkhögsskola motsvarande grundskolekompetens
(4) I__I = yrkesskola
(5) I__I = realskola
(6) I__I = flickskola
(7) I__I = fackskola
(8) I__I = gymnasieskola
(9) I__I = folkhögsskola motsvarande gymnasiekompetens
(10) I__I = högskola eller universitet

6. Hur många år har du gått i skola eller ägnat dig åt studier på heltid?
(Ange antal år från första klass och framåt)

I__I__I

Frågor rörande TOBAKSVANOR

7. Röker du cigaretter för närvarande?

(1) I__I = ja, regelbundet
(2) I__I = nej, ➧ gå till fråga **9**.
(3) I__I = ibland (mindre än 1 cigarett per dag) ➧ gå till fråga **9**.

8. Ungefär hur många cigaretter röker du i genomsnitt per dag?
(Ange antal cigaretter/dag)

I__I__I__I

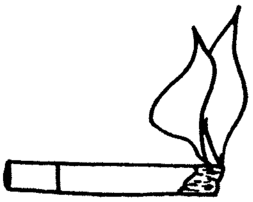

9. Har du någonsin rökt cigaretter tidigare?

(1) I__I = ja, regelbundet
(2) I__I = nej, aldrig ➧ gå till fråga **14**.
(3) I__I = ja, ibland

10. Hur gammal var du när du började röka cigaretter?
(Ange ålder i år)

I__I__I år

Frågorna 11 - 13 besvaras endast om du har slutat röka cigarette[illegible]

11. När slutade du röka? *(Ange årtal)*

I__I__I__I__I

12. Om du slutade röka under det senaste året, när slutade du?

(1) I__I = för mindre än en månad sedan
(2) I__I = för 1-6 månader sedan
(3) I__I = för 7-12 månader sedan

13. Vilken är den främsta orsaken till att du slutat röka?
(Ange endast ett alternativ)

(1) I__I = av hälsoskäl, på eget initiativ
(2) I__I = på inrådan av läkare/sjukvårdspersonal
(3) I__I = pga annan information/upplysning
(4) I__I = pga tryck från kamrater/familjemedlemmar
(5) I__I = av andra skäl

14. Hur många <u>cigarrer/cigariller</u> röker du per vecka?
(Ange antal cigarrer/cigariller per vecka)

I__I__I__I

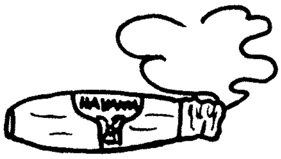

15. Hur många gram tobak röker du per vecka?
Ett paket piptobak väger 50 gram
(Ange antal gram/vecka)

I__I__I__I

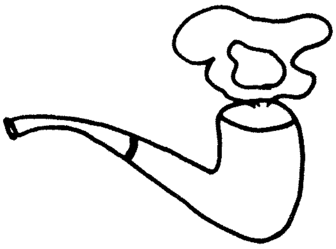

16. Har du någonsin använt snus?

(1) I__I = ja, snusade tidigare men inte nu
(2) I__I = ja, snusar mindre än 2 dosor per vecka
(3) I__I = ja, snusar 2-4 dosor per vecka
(4) I__I = ja, snusar mer än 4 men mindre än 7 dosor per vecka
(5) I__I = ja, snusar 7 dosor eller fler per vecka
(6) I__I = nej ➧ gå till fråga **19**

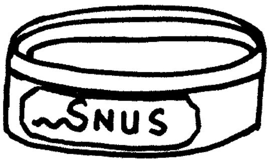

17. Hur många år har du använt snus?
(Ange antal år)

I__I__I

18. Började du att snusa i samband med att du slutade röka?

(1) I__I = ja
(2) I__I = nej
(3) I__I = jag både röker och snusar

Frågor rörande HÄLSA

19. Har du fått ditt blodtryck mätt under det senaste året?

(1) I__I = ja
(2) I__I = nej

20. Har du vid något tillfälle av läkare eller annan sjukvårds-personal fått besked om att du har högt blodtryck?

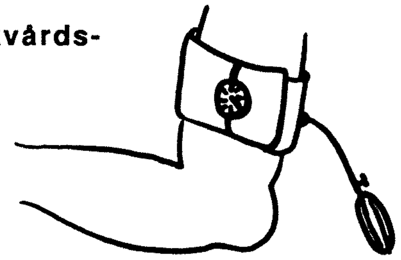

(1) I__I = ja
(2) I__I = nej ➧ gå till fråga 22

21. Har du under de senaste 2 veckorna tagit läkemedel mot förhöjt blodtryck?

(1) I__I = ja
(2) I__I = nej

22. Har du fått ditt kolesterol/blodfett mätt under det senaste året?

(1) I__I = ja
(2) I__I = nej

23. Har du vid något tillfälle av läkare eller annan sjukvårdspersonal fått besked om att du har förhöjda kolesterol-/blodfettvärden?

(1) I__I = ja
(2) I__I = nej ➧ gå till fråga 26.

24. Har läkare eller annan sjukvårdspersonal föreskrivet en särskild diet till dig för att sänka dina kolesterol-/blodfettnivåer?

(1) I__I = ja
(2) I__I = nej

25. Har du under de senaste två veckorna tagit medicin för att sänka kolesterol-/blodfettvärdet?

(1) I__I = ja
(2) I__I = nej
(8) I__I = vet ej om medicinen jag äter är för att sänka kolesterolet

26. Har du under de senaste två veckorna tagit acetylsalicylika t ex Albyl, Aspirin, Bamyl, Dispril, Magnecyl, Premaspin för att förebygga eller behandla hjärtsjukdom?

(1) I__I = ja
(2) I__I = jag använder acetylsalicylika regelbundet men för att behandla annan sjukdom än hjärtsjukdom
(3) I__I = nej, använder inte acetylsalicylika regelbundet.

27. Har du under de senaste två veckorna tagit acetylsalicylika t ex Albyl, Aspirin, Bamyl, Dispril, Magnecyl, Premaspin för att förebygga eller behandla slaganfall (propp i hjärnan)?

(1) I__I = ja
(2) I__I = jag använder acetylsalicylika regelbundet men för att behandla annan sjukdom än slaganfall (propp i hjärnan)
(3) I__I = nej, använder inte acetylsalicylika regelbundet.

28. Har du under de senaste 14 dagarna använt något läkemedel som ordinerats av läkare?

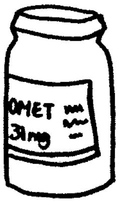

(1) I__I = ja
(2) I__I = nej

29. Om du svarat "ja" i så fall vilken eller vilka mediciner?

___

___

___

30. Har du under de senaste 14 dagarna använt någon annan medicin? (T.ex. naturmedicin, vitamin- eller järnpreparat, förebyggande medicin).

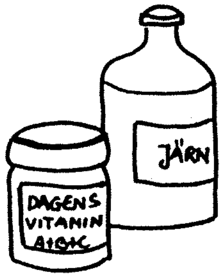

(1) I__I = ja
(2) I__I = nej

31. Om du svarat "ja" i så fall vilken eller vilka mediciner?

___

___

___

32. Får du smärtor - stickningar - ont i bröstet när du går uppför backar eller trappor, eller när du går fort på plan mark?

(1) I__I = ja
(2) I__I = nej

33. Får du smärtor - stickningar - ont i bröstet när du går i vanlig takt på plan mark?

(1) I__I = ja
(2) I__I = nej ➧ gå till fråga **38**.

34. Om du får smärtor eller obehag i bröstet i samband med att du rör dig, brukar du då?

(1) I__I = stanna
(2) I__I = sakta ner farten
(3) I__I = fortsätta i samma takt

35. Om du stannar eller saktar ner, försvinner smärtorna då?

(1) I__I = ja
(2) I__I = nej

36. Om de försvinner, hur snart försvinner de?

(1) I__I = efter mindre än 10 minuter
(2) I__I = efter mer än 10 minuter

37. VAR BRUKAR DU KÄNNA DESSA SMÄRTOR ELLER OBEHAG?
(Markera med X på figuren där du känner smärta eller obehag. Om smärta eller obehag känns ut i armarna eller upp mot halsen, markera med X även där)!

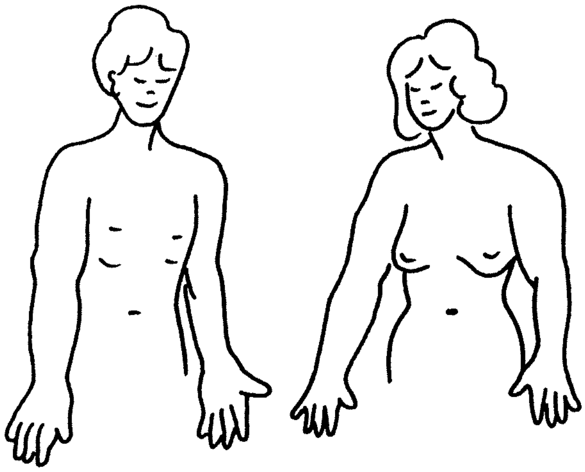

38. **Har du någon gång haft svår smärta i bröstet som varat i en halvtimme eller mer?**

(1) I__I = ja
(2) I__I = nej

39. **Har du legat på sjukhus för säker hjärtinfarkt (propp i hjärtat)?**

(1) I__I = ja, ange årtal I__I__I__I__I och sjukhus______________________________
(2) I__I = nej

40. **Brukar du få smärtor i vaderna, när du går i uppförsbackar, trappor, eller på plan mark?**

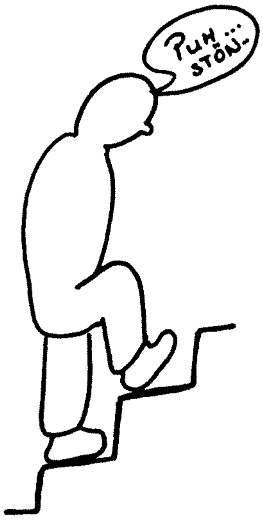

(1) I__I = ja
(2) I__I = nej

41. **Blir du andfådd av att gå två trappor upp eller motsvarande i samma takt som jämnåriga?**

(1) I__I = ja
(2) I__I = nej

42. **Har du haft slaganfall (hjärnblödning eller propp i hjärnan)?**

(1) I__I = ja, ange årtal I__I__I__I__I och sjukhus ______________________________
(2) I__I = nej

43. **Har du diabetes /sockersjuka?**

(1) I__I = ja
(2) I__I = nej ➡ gå till fråga **46.**

44. **Hur behandlar du din diabetes/sockersjuka?**

(1) I__I = insulin
(2) I__I = tabletter
(3) I__I = enbart kost
(4) I__I = ingetdera

45. **Hur gammal var du när du fick diabetes/sockersjuka?**

I__I__I år

46 Finns det någon i din släkt som avlidit i hjärtinfarkt <u>före</u> 65 års ålder? Med släkt menas i detta fall: Föräldrar, syskon, fastrar, farbröder, mostrar och morbröder.

(1) I__I = ja, min ____________________
(2) I__I = nej
(9) I__I = vet ej

47. Finns det någon i din släkt som avlidit i slaganfall (propp eller blödning i hjärnan) <u>före</u> 65 års ålder? Med släkt menas i detta fall: Föräldrar, syskon, fastrar, farbröder, mostrar och morbröder.

(1) I__I = ja, min ____________________
(2) I__I = nej
(9) I__I = vet ej

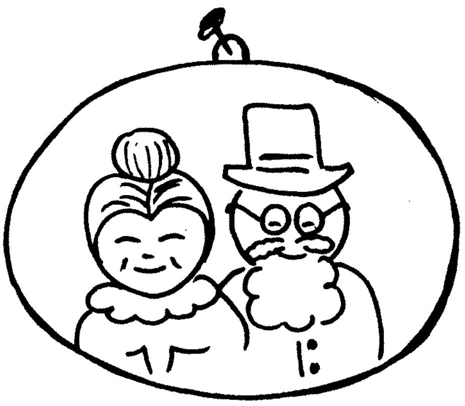

Besvara följande frågor genom att ange det svarsalternativ som bäst stämmer överens med din personliga situation/uppfattning

48. Hur bedömer du ditt allmänna hälsotillstånd?

(1) I__I = det är gott
(2) I__I = det är dåligt
(3) I__I = något däremellan

49. Hur bedömer du ditt allmänna hälsotillstånd jämfört med andra personer i din egen ålder?

(1) I__I = det är bättre
(2) I__I = det är sämre
(3) I__I = ungefär likadant

Frågor rörande FYSISK AKTIVITET

50. Hur mycket har du rört dig eller ansträngt dig kroppsligt på din fritid under det senaste året? *(Sätt bara X i ett av alternativen).*

(1) I__I = knappast något alls.

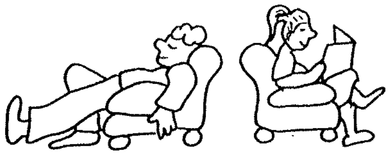

(2) I__I = mestadels stillasittande, ibland någon promenad eller liknande.

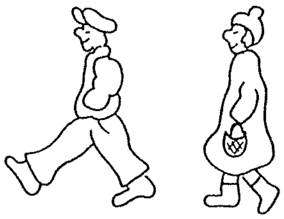

(3) I__I = lättare fysisk ansträngning minst 2 timmar i veckan, *t.ex gång och cykling (även till och från arbetet eller skolan) fiske, dans etc.*

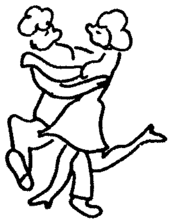

(4) I__I = mer ansträngande motion 1-2 timmar i veckan, *t.ex motionslöpning, tennis, simning, badminton, motionsgymnastik*

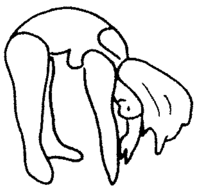

(5) I__I = mer ansträngande motion minst 3 timmar i veckan, *t.ex motionslöpning, tennis, simning, badminton, motionsgymnastik*

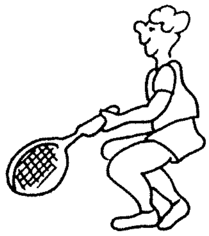

(6) I__I = hård träning eller tävning regelbundet och flera gånger i veckan där den fysiska ansträngningen är stor, *t.ex löpning, skidåkning, fotboll, simning*

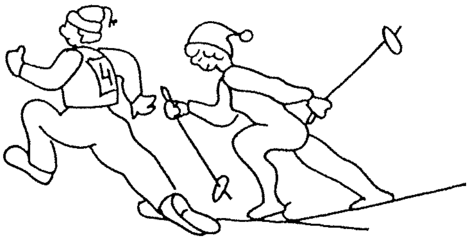

51. Hur mycket har du rört dig eller ansträngt dig kroppsligt i ditt arbete under det senaste året?
(Sätt bara X i ett av alternativen).

(1) I__I = stillasittande arbete. *Du har övervägande stillasittande arbete och går inte mycket ut under tiden t.ex skrivbordsarbete, urmakeri och montering av lättare delar.*

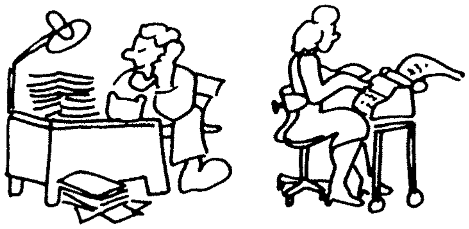

(2) I__I = lätt, men något rörligt arbete. *Du har ett arbete där du går ganska mycket men bär eller lyfter ej tyngre saker. T.ex rörligt expeditions-arbete, lätt industriarbete, förmanssysslor, sådan undervisning där är man går mycket, affärsbiträde, hushållsarbete.*

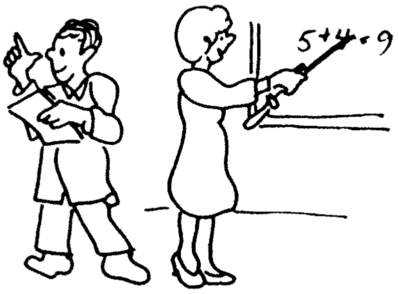

(3) I__I = måttligt tungt arbete. *Du går mycket och lyfter dessutom ganska mycket eller går uppför trappor eller backar. t.ex brevbärare, arbete vid tyngre industri, byggnadssnickeri, rörläggningsarbete, vårdarbete*

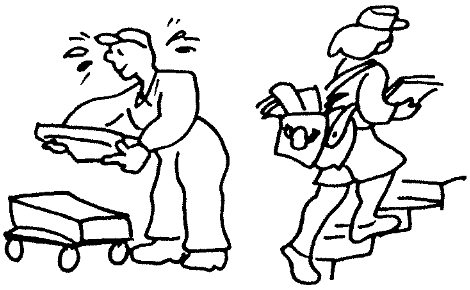

4) I__I = tungt arbete. *Du har ett tungt kroppsarbete, lyfter tunga föremål och anstränger dig mycket kroppsligt. T.ex skogsarbete, tungt lantbruksarbete, fiske med tunga redskap, byggnadsarbete.*

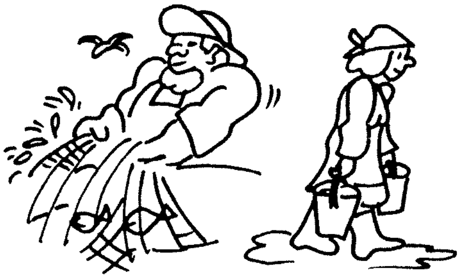

52. **Vilket är ditt yrke/din befattning?** Förvärvsarbetar du inte nu ska du ange yrke/sysselsättning som du tidigare huvudsakligen haft.

(Undvik allmänna yrkesbeteckningar som lärare, tjänsteman, byggnadsarbetare etc. Skriv i stället högstadielärare, kontorist, byggnadssnickare etc så att det framgår mer exakt vad du arbetar med).

____

____

53. **Beskriv kortfattat dina arbetsuppgifter, samt ange arbetsplats eller typ av företag.**

____

____

BESVARAS AV GIFTA/SAMBOENDE

54. **Vilket är din make/makas/samboendes yrke eller befattning?** *(Förvärvsarbetar han/hon inte nu ska du ange yrke/sysselsättning som han/hon tidigare huvudsakligen haft).*

____

____

55. **Beskriv kortfattat hans/hennes arbetsuppgifter.**

____

____

BESVARAS AV DE SOM ÄR EGNA FÖRETAGARE

56. **Hur många anställda har du i ditt företag?**

Antal anställda ______________

BESVARAS AV JORDBRUKARE/LANTBRUKARE

57. **Är ditt jordbruk/lantbruk att betrakta som ett litet eller stort jordbruk** *(Kryssa för det alternativ som stämmer bäst med din situation, samt ange hur mycket skog och åkermark du har).*

(1) I__I litet jordbruk hektar skog ______________

(2) I__I stort jordbruk hektar åkermark ___________

Med arbete avses din huvudsakliga sysselsättning.
OBS: Ange endast ett alternativ

58. Hur är dina nuvarande arbetsförhållanden?

(1) I__I = fast anställning, anställning tills vidare
(2) I__I = egen företagare
(3) I__I = tillfällig anställning, vikariat, beredskapsarbete
(4) I__I = arbetar i hemmet
(5) I__I = arbetslös ➧ gå till fråga **81**
(6) I__I = studerande ➧ gå till fråga **79**
(7) I__I = pensionerad ➧ gå till fråga **80**

59. Hur många timmar förvärvsarbetar du i normala fall per vecka?

(1) I__I = jag förvärvsarbetar inte
(2) I__I = mindre än 15 timmar
(3) I__I = 15-35 timmar
(4) I__I = mer än 35 timmar

60. Vad har du för arbetstider i normala fall?

(1) I__I = jag förvärvsarbetar inte
(2) I__I = fast arbetstid (t ex 7-16 eller 8-17)
(3) I__I = skiftarbete (t ex 2-skift, 3-skift, 5-skift)
(4) I__I = varierande arbetstider (ibland dagar, ibland kvällar, ibland helger)

61. Är ditt arbete fysiskt (kroppsligt) tungt?

(1) I__I = ja, ofta
(2) I__I = ja, ibland
(3) I__I = nej, sällan
(4) I__I = nej, så gott som aldrig

62. Kräver ditt arbete att du arbetar mycket fort?

(1) I__I = ja, ofta
(2) I__I = ja, ibland
(3) I__I = nej, sällan
(4) I__I = nej, så gott som aldrig

63. Är ditt arbete psykiskt påfrestande?

(1) I__I = ja, ofta
(2) I__I = ja, ibland
(3) I__I = nej, sällan
(4) I__I = nej, så gott som aldrig

64. Har du tillräckligt med tid för att hinna med arbetsuppgifterna?

(1) I__I = ja, ofta
(2) I__I = ja, ibland
(3) I__I = nej, sällan
(4) I__I = nej, så gott som aldrig

65. Förekommer det ofta motstridiga krav i ditt arbete?

(1) I__I = ja, ofta
(2) I__I = ja, ibland
(3) I__I = nej, sällan
(4) I__I = nej, så gott som aldrig

66. Får du lära dig nya saker i ditt arbete?

(1) I__I = ja, ofta
(2) I__I = ja, ibland
(3) I__I = nej, sällan
(4) I__I = nej, så gott som aldrig

67. Kräver ditt arbete skicklighet?

(1) I__I = ja, ofta
(2) I__I = ja, ibland
(3) I__I = nej, sällan
(4) I__I = nej, så gott som aldrig

68. Kräver ditt arbete påhittighet?

(1) I__I = ja, ofta
(2) I__I = ja, ibland
(3) I__I = nej, sällan
(4) I__I = nej, så gott som aldrig

69. Innebär ditt arbete att man gör samma sak om och om igen?

(1) I__I = ja, ofta
(2) I__I = ja, ibland
(3) I__I = nej, sällan
(4) I__I = nej, så gott som aldrig

70. Har du frihet att bestämma <u>hur</u> ditt arbete skall utföras?

(1) I__I = ja, ofta
(2) I__I = ja, ibland
(3) I__I = nej, sällan
(4) I__I = nej, så gott som aldrig

71. Har du frihet att bestämma <u>vad</u> som skall utföras i ditt arbete?

(1) I__I = ja, ofta
(2) I__I = ja, ibland
(3) I__I = nej, sällan
(4) I__I = nej, så gott som aldrig

72. Har du i vanliga fall möjlighet att tala med dina arbetskamrater under raster, om du skulle vilja det?

(1) I__I = ja, alltid
(2) I__I = ja, för det mesta
(3) I__I = nej, jag har inga raster
(4) I__I = nej, jag har inga raster tillsammans med arbetskamrarter

73. Är ditt arbete av den karaktären att du kan lämna det ett tag om du vill tala med en arbetskamrat?

(1) I__I = ja, för det mesta
(2) I__I = ja, ibland
(3) I__I = bara för brådskande ärenden
(4) I__I = nej, det är helt omöjligt

74. Har du, som en del i ditt arbete, en mängd kontakter med dina arbetskamrater?

(1) I__I = ja, ständigt nya kontakter i arbetet
(2) I__I = ja, ibland kontakter i arbetet
(3) I__I = nej, jag arbetar för det mesta ensam
(4) I__I = nej, jag arbetar ständigt ensam

75. Hur ofta är du vanligen tillsammans med en eller flera av dina arbetskamrater på fritid?

(1) I__I = en eller flera ggr/vecka
(2) I__I = en eller ett par ggr/månad
(3) I__I = en eller ett par ggr/år
(4) I__I = sällan eller aldrig

76. När besökte någon av dina arbetskamrater dig senast?

1) I__I = för en till fyra veckor sedan
(2) I__I = för en till tolv månader sedan
(3) I__I = för mer än ett år sedan
(4) I__I = har aldrig haft besök av någon arbetskamrat

77. Tror du att det finns risk att du blir arbetslös?

(1) I__I = ja, inom den närmaste tiden
(2) I__I = ja, på längre sikt
(3) I__I = nej

78. Antag att du har ett arbete och blir uppsagd. Hur stora chanser tror du att du har att få ett nytt arbete inom en månad?

(1) I__I = mycket stora
(2) I__I = ganska stora
(3) I__I = små
(4) I__I = inga chanser

79. På vilken nivå studerar du?
(Denna fråga besvaras endast av studerande)

(1) I__I = grundskolenivå
(2) I__I = gymnasial nivå
(3) I__I = eftergymnasial nivå
(4) I__I = annan, vilken ____________________

80. Har du varit arbetslös någon gång?

(1) I__I = ja, för mer än 12 månader sedan
(2) I__I = ja, någon gång under de senaste 12 månaderna
(3) I__I = nej, aldrig ➡ gå till fråga **82**

81. Om du varit/eller är arbetslös, hur länge har du sammanlagt varit det?

(1) I__I = mindre än 3 månader
(2) I__I = 3-12 månader
(3) I__I = mer än 12 månader

82. Har du tvingats flytta någon gång?

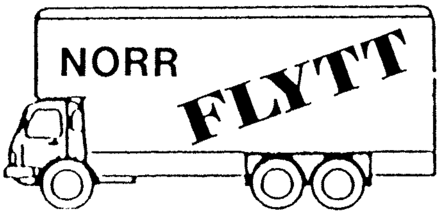

(1) I__I = ja, för mer än 12 månader sedan
(2) I__I = ja, de senaste 12 månaderna
(3) I__I = nej aldrig ➡ gå till fråga **84**

83. Vilken var anledningen till flyttningen?

(1) I__I = familjeskäl
(2) I__I = arbetsmarknadsskäl
(3) I__I = annat

84. Har någon eller några personer som betytt eller betyder mycket för dig, flyttat så att du inte längre kan umgås med honom/henne eller dem?

(1) I__I = ja
(2) I__I = nej ➡ gå till fråga **86**

85. Om du svarat "ja" på föregående fråga, vilken var anledningen till flyttningen?

(1) I__I = familjeskäl
(2) I__I = arbetsmarknadsskäl
(3) I__I = annat skäl

86. Är du just nu sjukskriven, har ålderpension, förtidspension, sjukpension eller sjukbidrag?

(1) I__I = ja, ålderpension/sjukpension på <u>heltid</u>
(2) I__I = ja, ålderpension/sjukpension på <u>deltid</u>
(3) I__I = ja, förtidspension/sjukbidrag
(4) I__I = ja, sjukskriven
(5) I__I = nej

87. Ibland när man äter på restaurang får man matfettet i portionsförpackningar. Hur stor del av ett sådant paket a' 10 gram motsvarar den mängd som du brukar använda på en brödskiva?
(Ange endast ett alternativ. Markera med X i lämplig ruta)

(1) I__I = inget
(2) I__I = högst 1/3 av paketet
(3) I__I = ungefär 1/2 av paketet
(4) I__I = ungefär 2/3 av paketet
(5) I__I = hela paketet
(6) I__I = mer än ett paket

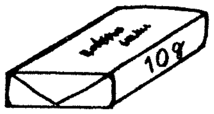

88. Hur är det kaffe du dricker vanligtvis tillagat?
(Ange endast ett alternativ. Markera med X i lämplig ruta)

(1) I__I = jag dricker mest kokkaffe
(2) I__I = jag dricker mest bryggkaffe
(3) I__I = annat (t ex snabbkaffe)
(4) I__I = jag dricker mest kaffe tillagat i sk. perkulator
(5) I__I = jag dricker inte kaffe ➡ gå till fråga **90**

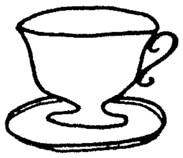

89. Hur många koppar kaffe dricker du i genomsnitt varje dag?

I__I__I koppar

90. Vilket av följande alternativ på morgonmål överensstämmer bäst med ditt eget?

(1) I__I = kaffe eller te
(2) I__I = kaffe/te och smörgås
(3) I__I = kaffe/te och vetebröd eller skorpor
(4) I__I = fil, flingor och smörgås
(5) I__I = gröt och smörgås
(6) I__I = välling
(7) I__I = jag äter inget alls

91. Hur mycket mjölk och eller fil äter eller dricker du? Om du inte dricker/äter mjölk/fil dagligen så försök uppskatta antalet glas per vecka. Om du inte alls dricker/äter mjölk/fil ange 00 som svarsalternativ. *(Obs! Flera alternativ kan förekomma)*

(1) I__I__I glas standardmjölk/fil per dag *eller* I__I__I glas standardmjölk/fil per vecka
(2) I__I__I glas mellanmjölk per dag *eller* I__I__I glas mellanmjölk /per vecka
(3) I__I__I glas lättmjölk/fil per dag *eller* I__I__I glas lättmjölk/fil per vecka

92. Använder du extrasaltat matfett?

(1) I__I = ja
(2) I__I = nej

93. Har du ändrat dina matvanor under de senaste två åren?

(1) I__I = ja
(2) I__I = nej ➡ gå till fråga **96**
(3) I__I = vet ej

94. På vilket sätt har du ändrat dina matvanor?
(Kryssa för det eller de alternativ som stämmer bäst)

(1) I__I minskat mängden fett
(2) I__I övergått till mera fleromättat fett
(3) I__I ökat användningen av fiberrika produkter t ex bröd och rotsaker
(4) I__I minskat mängden salt
(5) I__I minskat mängden socker
(6) I__I ökat mängden färsk frukt eller grönsaker

95. Varför har du ändrat dina matvanor?
(Kryssa för det eller de alternativ som stämmer bäst)

(1) I__I = föreskriven diet pga sjukdom
(2) I__I = påverkan (t ex tidningar, vänner)
(3) I__I = för att gå ner i vikt
(4) I__I = annat

96. Har du ändrat din vikt under det senaste året?

(1) I__I = ökat i vikt
(2) I__I = står på samma vikt
(3) I__I = minskat i vikt

97. Har du dina egna tänder kvar eller har du tandprotes i överkäken?

(1) I__I = enbart egna tänder
(2) I__I = egna tänder + tandprotes
(3) I__I = enbart tandprotes

98. Har du dina egna tänder kvar eller har du tandprotes i underkäken?

(1) I__I = enbart egna tänder
(2) I__I = egna tänder + tandprotes
(3) I__I = enbart tandprotes

99. Hur ofta äter du något från nedanstående grupper av livsmedel? 18

Ange endast ett svarsalternativ på varje rad. Markera tydligt med X i lämplig ruta.

Frekvens<br>Födo ämne	Aldrig	Någon gång per år	1-3 ggr per månad	1 gång per vecka	2-3 ggr per vecka	4-6 ggr per vecka	1 gång per dag	2-3 ggr per dag	4ggr per dag el mer
Bregott på smörgås 01									
Smör på smörgås 02									
Lättmargarin på smörgås (ex Lätta) 03									
Margarin på smörgås 04									
Smör till matlagning 05									
Margarin till matlagning 06									
Olja till matlagning eller dressing 07									
Hårdost 08									
Fil, yoghurt, kefir 09									
Lättfil, lättyoghurt 10									
Grädde, créme fraiche gräddfil 11									
Hårt bröd (ex husmans) 12									
Grovt mjukt fullkornsbröd 13									
Kokt eller bakad potatis 14									
Stekt potatis 15									
Pommes frites 16									

Frekvens<br>Födo ämne	Aldrig	Någon gång per år	1-3 ggr per månad	1 gång per vecka	2-3 ggr per vecka	4-6 ggr per vecka	1 gång per dag	2-3 ggr per dag	4ggr per dag el mer
Rotfrukter morötter mfl 17									
Vitkål 18									
Tomat, gurka 19									
Sallad, isbergssallad salladskål 20									
Spenat, grönkål 21									
Applen,päron persikor plommon 22									
Apelsin mandarin grapefrukt 23									
Banan 24									
Bär (färska el djupfrysta) 25									
Gröt av havre 26									
Flingor mysli, bryta 27									
Ris 28									
Spagetti makaroner 29									
Palt kroppkakor 30									
Pizza 31									
Bacon, sidfläsk, "syltefläsk" 32									
Korv (som maträtt) 33									
Korv (som pålägg) 34									

Frekvens Födo ämne	Aldrig	Någon gång per år	1-3 ggr per månad	1 gång per vecka	2-3 ggr per vecka	4-6 ggr per vecka	1 gång per dag	2-3 ggr per dag	4ggr per dag el mer
Kyckling höns 35									
Agg,ägg-rätter omeletter 36									
Mager fisk (ex gädda aborre,torsk mfl) 37									
Fet fisk (ex lax, röding strömming sik mfl) 38									
Salt fisk (salt ström-ming, salt sill mfl) 39									
Chips, pop-corn, salta nötter m m 40									
Lättmjölk 41									
Mellanmjölk 1,5 % 42									
Standard-mjölk, 3 % 43									
Starköl 44									
Vin 45									
Sprit 46									
Läskedrycker Coca-Cola mm 47									
Juice 48									
Kaffe 49									

monica centre
WHO/OMS
northern sweden

Plats för etikett

VÄRLDSHÄLSOORGANISATIONENS KAMPANJ MOT HJÄRT-/KÄRLSJUKDOMAR

FRÅGEFORMULÄR DEL 3

Frågor rörande SOCIALT STÖD

De följande frågorna handlar om dina personliga förhållanden och om människorna i din omgivning. Frågorna handlar både om människor som står dig mycket nära, om vänner och om bekanta.
Vissa frågor kan kanske kännas främmande men försök att besvara dem ändå! Några frågor kan kännas väldigt lika, även om formuleringarna är olika. Försök ändå att besvara dem så noggrant som möjligt.

Endast ett X på varje fråga där annat ej anges

100. **Hur många människor känner du och har kontakt med, som har samma intressen som du?** Det gäller kontakter både i arbetet och på fritiden.

(1) I__I = ingen
(2) I__I = 1-2 personer
(3) I__I = 3-5 personer
(4) I__I = 6-10 personer
(5) I__I = 11-15 personer
(6) I__I = mer än 15 personer

101. **Hur många människor, som du känner, träffar du eller samtalar du med under en vanlig vecka?** Räkna ej med människor som du träffar tillfälligtvis och som du knappast kommer att återse, t ex kunder i en affär.

(1) I__I = ingen
(2) I__I = 1-2 personer
(3) I__I = 3-5 personer
(4) I__I = 6-10 personer
(5) I__I = 11-15 personer
(6) I__I = mer än 15 personer

102. **Är det ungefär lagom många människor du träffar under ditt dagliga liv?** Skulle du vilja träffa fler eller färre människor?

(1) I__I = färre
(2) I__I = lagom många
(3) I__I = fler

103. **Hur många vänner har du som kan komma hem till dig när som helst och känna sig hemma?** De skulle inte bry sig om, om det var ostädat eller om du höll på att äta. *(Nära släktingar skall inte räknas med!)*

(1) I__I = ingen
(2) I__I = 1-2 personer
(3) I__I = 3-5 personer
(4) I__I = 6-10 personer
(5) I__I = 11-15 personer
(6) I__I = mer än 15 personer

104. **Hur många finns det, i din familj och bland dina vänner, som du kan tala öppet med utan att behöva tänka dig för?**

(1) I__I = ingen
(2) I__I = 1-2 personer
(3) I__I = 3-5 personer
(4) I__I = 6-10 personer
(5) I__I = 11-15 personer
(6) I__I = mer än 15 personer

105. **Finns det någon särskild person som du känner att du verkligen kan få stöd av?**

(1) I__I = ja
(2) I__I = ja, men jag behöver inte det.
(3) I__I = nej

106. **Finns det någon särskild person som känner sig stå väldigt nära dig?**

(1) I__I = ja
(2) I__I = är inte säker
(3) I__I = nej

107. **Har du någon särskild person som du kan dela dina känslor med när du känner dig lycklig?** Någon som själv skulle känner sig lycklig bara för att du är det?

(1) I__I = ja
(2) I__I = nej

108. **Har du någon du kan dela dina innersta känslor med och anförtro dig åt?**

(1) I__I = ja
(2) I__I = nej

109. **Händer det att någon håller om dig till tröst eller stöd?**

(1) I__I = ja
(2) I__I = nej

110. **Tror du att de där hemma eller några andra verkligen uppskattar vad du gör för dem?**

(1) I__I = ja
(2) I__I = inte tillräckligt
(3) I__I = nej, inte alls

111. **Finns det människor i din omgivning som du lätt kan be om saker, t.ex. människor som du känner så väl att du kan låna verktyg eller köksgrejor?**

(1) I__I = nej
(2) I__I = ja

112. **Bortsett från dem där hemma, finns det någon som du kan vända dig till om du är i svårigheter?** Någon som du lätt kan träffa och som du litar på och kan få verklig hjälp av när du har det besvärligt?

(1) I__I = nej
(2) I__I = ja

113. **Innehåller socker vitaminer?**

(1) I__I = ja, rätt mycket
(2) I__I = en del
(3) I__I = inte alls
(4) I__I = vet ej

114. **Vad är kolesterol?**

(1) I__I = hormon
(2) I__I = mineral
(3) I__I = blodfett
(4) I__I = vet ej

115. **Hur är "vanlig" mjölk (3% fett) jämfört med lättmjölk (0,5% fett) ur åderförkalkningssynpunkt för friska, vuxna individer?**

(1) I__I = mer hälsosam
(2) I__I = lika hälsosam
(3) I__I = mindre hälsosam
(4) I__I = vet ej

116. **Vanligt bordssalt består av?**

(1) I__I = natriumklorid
(2) I__I = kaliumklorid
(3) I__I = magnesiumklorid
(4) I__I = vet ej

117. **Vilken av följande ämnen ökar blodtrycket?**

(1) I__I = natrium
(2) I__I = kalium
(3) I__I = magnesium
(4) I__I = vet ej

118. **Varifrån får svenskarna det mesta av sitt saltintag?**

(1) I__I = de saltar på vid matbordet
(2) I__I = salttillsats vid framställning av maten
(3) I__I = från saltad fisk
(4) I__I = vet ej

119. **Om en medelålders man får intensiva smärtor i bröstet som räcker mer än en halvtimme bör denne?**

(1) I__I = ta en smärtstillande medicin
(2) I__I = beställa tid hos doktorn
(3) I__I = ta det lugnare i framtiden
(4) I__I = omedelbart söka läkare eller sjukhus
(5) I__I = vet ej

IFYLLES ENDAST AV KVINNOR

120. Har du fortfarande menstruation varje månad?

(1) I__I = ja, regelbundet varje månad ➡ gå till fråga **123**
(2) I__I = ibland, men inte så regelbundet som tidigare, eller att menstruationen har upphört de senaste fem månaderna, ➡ gå till fråga **122**
(3) I__I = nej, det är mer än sex månader sedan den upphörde
(8) I__I = jag är gravid, ➡ gå till fråga **123**

121. Hur gammal var du när menstruationerna fullständigt upphörde?
(Ifylles av kvinnor som inte längre har menstruation)

I__I__I

122. Har du tagit hormontabletter (östrogen) för dina klimakteriebesvär under den senaste månaden?
(Ifylles av kvinnor som inte längre har menstruation)

(1) I__I = ja
(2) I__I = nej

123. Har du tagit preventivmedel (p-piller) eller preventivinjektion (p-spruta) de senaste två månaderna?

(1) I__I = ja
(2) I__I = nej
