# Supplementary material for: Time trends of comparative self-rated health in adults aged 25-34 in the Northern Sweden MONICA study, 1990-2014
Source: PLoS One. 2017 Nov 20;12(11):e0187896. doi: 10.1371/journal.pone.0187896 (PMC5695772; doi:10.1371/journal.pone.0187896)
Supplement: S2 Text — (PDF) [file pone.0187896.s002.pdf]

WHO

MONICA - projektet

6732

1

VIKTIGT!

☆ Använd **endast blyertspenna**
☆ **Fyll hela** rutan
☆ **Sudda noga** *(kryssa ej över)* markeringar som du vill ändra
☆ **Vik ej** detta papper

Markera så:

Inte så:

OBS! Alla frågor skall besvaras med endast en markering!

— CIVILSTÅND och BOENDE —

1. Vilket är ditt nuvarande civilstånd?

- = ogift
- = gift eller sammanboende
- = skild eller separerad
- = änka/änkling
- = annat ....

2. Vilka personer sammanbor du med?

- = enbart en vuxen (make, maka, sambo)
- = enbart barn, ange antal |__|__|
- = vuxen och barn, ange antal |__|__|
- = annan/andra
- = bor helt ensam

3. Var bor du?

- = större tätort - Umeå, Skellefteå, Luleå, Boden, Piteå, Kiruna- orter med mer än 15 000 invånare
- = annan tätort med mer än 1 000 invånare
- = ort med mindre än 1 000 invånare

4. I vilket land är du född?

- = Sverige
- = Finland
- = annat land ....

5. Var är du född? *(Ange vilken kyrkoförsamling eller ort du tillhörde vid födseln)?*

....

församling/ort län

6. Vilken är den högsta utbildningsnivå som du har avslutat?

- = folkskola
- = grundskola
- = folkhögsskola motsvarande grundskolekompetens
- = yrkesskola
- = realskola
- = flickskola
- = fackskola
- = gymnasieskola
- = folkhögsskola motsvarande gymnasiekompetens
- = högskola eller universitet

7. Hur många år har du gått i skola eller ägnat dig åt studier på heltid? *(Ange antal år från första klass och framåt)*

|__|__|

WHO

MONICA - projektet

VIKTIGT!

☆ Använd **endast blyertspenna**
☆ **Fyll hela** rutan
☆ **Sudda noga** *(kryssa ej över)* markeringar som du vill ändra
☆ **Vik ej** detta papper

Markera så: 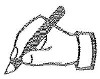

Inte så:

6732

2

TOBAKSVANOR

8. Röker du cigaretter för närvarande?

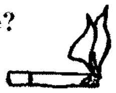

- = ja, regelbundet (1 cigarett eller mer per dag) => gå till fråga **9**
- = nej, => gå till fråga **12**
- = ibland (mindre än en cigarett/dag) => gå till fråga **10**

9. Ungefär hur många cigaretter röker du i genomsnitt per dag? *(Ange antal cigaretter/dag)*

|__|__|__| => gå till fråga **16**

10. Hur många dagar i veckan röker du cigaretter?

- = vanligtvis en dag eller mindre
- = vanligtvis 2 till 4 dagar
- = nästan varje dag

11. Ungefär hur många cigaretter röker du i genomsnitt per vecka

|__|__|__|

12. Har du någonsin rökt cigaretter regelbundet tidigare?

- = ja, regelbundet => gå till fråga **13**
- = nej => gå till fråga **18**

13. När slutade du att röka cigaretter regelbundet? *(Ange årtal)*

19 |__|__|

14. Om du slutade röka under det senaste året, när slutade du?

- = för mindre än en månad sedan
- = för 1-6 månader sedan
- = för 7-12 månader sedan

15. Vilken är den främsta orsaken till att du slutat röka? *(Ange endast ett alternativ)*

- = av hälsoskäl, på eget initiativ
- = på inrådan av läkare/sjukvårdspersonal
- = pga annan information/upplysning
- = pga tryck från kamrater/familjemedlemmar
- = av andra skäl

16. Vilket är det högsta antal cigaretter/dag, som du rökt under en så lång period som ett år.

|__|__|__|

17. Hur gammal var du när du började röka cigaretter regelbundet? *(Ange ålder i år)*

|__|__|

18. Har du någonsin rökt pipa?

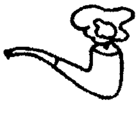

- = ja, för närvarande regelbundet => gå till fråga **19**
- = nej => gå till fråga **20**
- = jag röker pipa ibland (mindre än 1 gång/dag) => gå till fråga **19**
- = tidigare, men inte nu => gå till fråga **20**

19. Hur många gram piptobak röker du per vecka?
Ett paket piptobak väger som regel 50 gram
(Ange antal gram/vecka)

|__|__|__|

20. Har du någonsin rökt cigarrer eller cigariller?

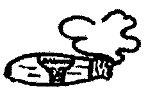

- = ja för närvarande regelbundet => gå till fråga **21**
- = nej => gå till fråga **22**
- = jag röker cigarrer/cigariller ibland (mindre än 1/dag) => gå till fråga **21**
- = tidigare, men inte nu => gå till fråga **22**

WHO

MONICA - projektet

VIKTIGT!

- ☆ Använd **endast blyertspenna**
- ☆ **Fyll hela** rutan
- ☆ **Sudda noga** *(kryssa ej över)* markeringar som du vill ändra
- ☆ **Vik ej** detta papper

Markera så:

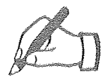

⊂⊃ ⊂⊃ ⊂⊃ ▬ ⊂⊃

Inte så:

6732

3

21. Hur många cigarrer/cigariller röker du per vecka? *(Ange antal cigarrer/cigariller per vecka)*

|__|__|__|

OBS! Fråga 22 skall endast fyllas i av icke-rökare och de som röker ibland

22. Hur många timmar i medeltal per dag, är du i närheten av andra människors tobaksrök? *(Ange antal timmar/dag du inandas eller känner lukten av tobaksrök)*

|__|__|

23. Har du någonsin använt snus?

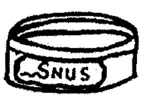

- = ja, snusade tidigare men inte nu
- = ja, snusar mindre än 2 dosor per vecka
- = ja, snusar 2 - 4 dosor per vecka
- = ja, snusar mer än 4 men mindre än 7 dosor per vecka
- = ja, snusar 7 dosor eller fler per vecka
- = nej => gå till fråga **26**

24. Hur många år har du använt snus? *(Ange antal år)*

|__|__|

25. Började du att snusa i samband med att du slutade röka?

- = ja
- = nej
- = jag både röker och snusar

26. Använder du dagligen andra nikotinprodukter? *(Gäller ej cigarett, cigarr, pipa eller snus)*

- = ja, tuggtobak
- = ja, nikotintuggummi
- = ja, nikotinplåster
- = annat..
- = nej

— HÄLSA —

27. Har du vid något tillfälle av läkare eller annan sjukvårdspersonal fått besked om att du har högt blodtryck?

- = ja
- = nej => gå tillfråga **29**

28. Har du under de senaste 2 veckorna tagit läkemedel mot förhöjt blodtryck?

- = ja
- = nej
- = osäker om den medicin jag äter är mot högt blodtryck

29. Har du fått ditt blodtryck mätt under det senaste året?

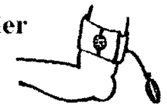

- = ja
- = nej

30. Har du vid något tillfälle av läkare eller annan sjukvårdspersonal fått besked om att du har förhöjda kolesterol-/blodfettvärden?

- = ja
- = nej => gå tillfråga **33**

31. Har läkare eller annan sjukvårdspersonal föreskrivit en särskild diet till dig för att sänka dina kolesterol-/blodfettnivåer?

- = ja
- = nej
- = jag håller en föreskriven diet, men vet ej om den är till för att sänka mina kolesterol-/blodfettnivåer

WHO **MONICA** - projektet

6732

4

VIKTIGT!

- ☆ Använd **endast blyertspenna**
- ☆ **Fyll hela** rutan
- ☆ **Sudda noga** *(kryssa ej över)* markeringar som du vill ändra
- ☆ **Vik ej** detta papper

Markera så:

Inte så:

32. Har du under de senaste två veckorna tagit medicin föreskriven av läkare, för att sänka kolesterol- /blodfettvärdet?
- = ja
- = nej
- = vet ej om medicinen jag äter är för att sänka kolesterolet

33. Har du fått ditt kolesterol/blodfett mätt under det senaste året?
- = ja
- = nej

34. Har du under de senaste två veckorna tagit acetylsalicylika t ex Albyl, Aspirin, Bamyl, Bamycor, Dispril, Trombyl, Magnecyl för att förebygga eller behandla hjärtsjukdom?
- = ja
- = nej
- = nej, men jag använder acetylsalicylika regelbundet för att behandla annan sjukdom än hjärtsjukdom

35. Har du under de senaste två veckorna tagit acetylsalicylika t ex Albyl, Aspirin, Bamyl, Bamycor, Dispril, Trombyl, Magnecyl för att förebygga eller behandla slaganfall (propp i hjärnan)?
- = ja
- = nej, använder inte acetylsalicylika regelbundet.
- = nej, men jag använder acetylsalicylika regelbundet för att behandla annan sjukdom än hjärtsjukdom

36. Har du under de senaste 14 dagarna använt något läkemedel som ordinerats av läkare?
- = ja
- = nej

37. Om du svarat "ja" i så fall vilken eller vilka mediciner?

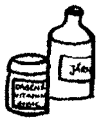

1__________ 2 __________

3__________ 4 __________

5__________ 6 __________

38. Har du under de senaste 14 dagarna använt någon annan medicin? *(T.ex. naturmedicin, vitamin- eller järnpreparat, förebyggande medicin).*
- = ja
- = nej

39. Om du svarat "ja" i så fall vilken eller vilka mediciner?

1__________ 2 __________

3__________ 4 __________

40. Får du smärtor - stickningar - ont i bröstet när du går uppför backar eller trappor, eller när du går fort på plan mark?
- = ja
- = nej

41. Får du smärtor - stickningar - ont i bröstet när du går i vanlig takt på plan mark?
- = ja
- = nej

Om du svarat nej på både fråga 40 och 41 => gå till fråga **46**

42. Om du får smärtor eller obehag i bröstet i samband med att du rör dig, brukar du då?...
- = stanna
- = sakta ner farten
- = fortsätta i samma takt

43. Om du stannar eller saktar ner, försvinner smärtorna då?
- = ja
- = nej

44. Om de försvinner, hur snart försvinner de?
- = efter mindre än 10 minuter
- = efter mer än 10 minuter

WHO

MONICA - projektet

VIKTIGT!

☆ Använd **endast blyertspenna**
☆ **Fyll hela** rutan
☆ **Sudda noga** *(kryssa ej över)* markeringar som du vill ändra
☆ **Vik ej** detta papper

Markera så:

Inte så:

6732

5

45. VAR BRUKAR DU KÄNNA DESSA SMÄRTOR ELLER OBEHAG? *(Markera med X på figuren där du känner smärta eller obehag. Om smärta eller obehag känns ut i armarna eller upp mot halsen, markera med X även där)!*

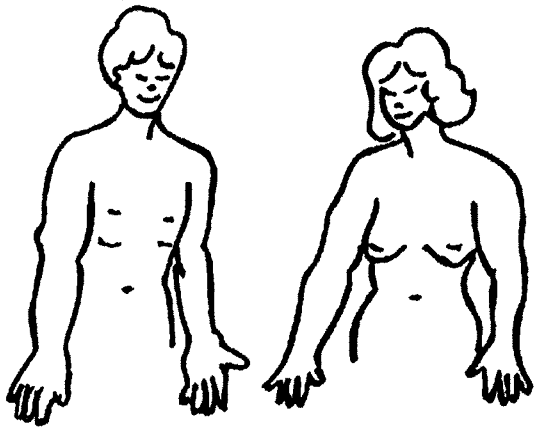

46. Har du någon gång haft svår smärta i bröstet som varat i en halvtimme eller mer?
- = ja
- = nej

47. Har du legat på sjukhus för säker hjärtinfarkt (propp i hjärtat)?
- = ja, ange årtal 19|__|__| och sjukhus............................
- = nej
- = vet ej om det var en säker hjärtinfarkt

48. Brukar du få smärtor i vaderna, när du går i uppförsbackar, trappor eller på plan mark?
- = ja
- = nej

49. Blir du andfådd av att gå två trappor upp eller motsvarande i samma takt som jämnåriga?
- = ja
- = nej

50. Har du haft slaganfall (hjärnblödning eller propp i hjärnan)?
- = ja, ange årtal 19 |__|__| och sjukhus..........................
- = nej

51. Har du diabetes/sockersjuka?
- = ja
- = nej => gå till fråga **54**

52. Hur behandlar du din diabetes/sockersjuka?
- = insulin
- = tabletter
- = enbart kost
- = ingetdera

53. Hur gammal var du när du fick diabetes/sockersjuka?
|__|__| år

54. Har du av läkare fått diagnosen glutenintolerans (celiaki)?
- = ja
- = nej

55. Mår du dåligt om du äter mycket mat med mjöl i?*(Tex. bröd, gröt, pannkaka, palt, pizza, pasta)*
- = ja
- = nej

56. Har du haft mångåriga magbesvär?
- = ja
- = nej

WHO

MONICA - projektet

6732 | **6**

VIKTIGT!

☆ Använd **endast blyertspenna**
☆ **Fyll hela** rutan
☆ **Sudda noga** *(kryssa ej över)* markeringar som du vill ändra
☆ **Vik ej** detta papper

Markera så: ⬭ ⬭ ⬭ ⬬ ⬭

Inte så:

57. Finns det någon i din släkt som avlidit i hjärtinfarkt före 65 års ålder?
Med släkt menas i detta fall: Föräldrar, syskon, fastrar, farbröder, mostrar och morbröder.

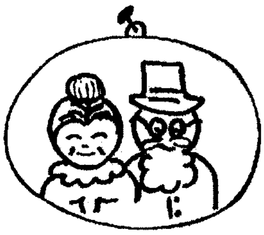

- ⬭ = ja, min mamma — **Hon var då............ år**
- ⬭ = ja, min pappa — **Han var då............ år**
- ⬭ = ja, något/några syskon — **Han/hon var då.........år**
- ⬭ = ja, någon/några av fastrar, farbröder,
- ⬭ mostrar och morbröder — **Han/hon var då.........år**
- ⬭ = nej
- ⬭ = vet ej

58. Finns det någon i din släkt som avlidit i slaganfall (propp eller blödning i hjärnan) före 65 års ålder? *Med släkt menas i detta fall: Föräldrar, syskon, fastrar, farbröder, mostrar och morbröder.*

- ⬭ = ja, min mamma — **Hon var då............ år**
- ⬭ = ja, min pappa — **Han var då............ år**
- ⬭ = ja, något/några syskon — **Han/hon var då.........år**
- ⬭ = ja, någon/några av fastrar, farbröder,
- ⬭ mostrar och morbröder — **Han/hon var då.........år**
- ⬭ = nej
- ⬭ = vet ej

Besvara följande frågor genom att ange det svarsalternativ som bäst stämmer överens med din personliga situation/uppfattning.

59. Hur bedömer du ditt allmänna hälsotillstånd?

- ⬭ = det är gott
- ⬭ = det är dåligt
- ⬭ = något däremellan

60. Hur bedömer du ditt allmänna hälsotillstånd jämfört med andra personer i din egen ålder?

- ⬭ = det är bättre
- ⬭ = det är sämre
- ⬭ = ungefär likadant

WHO

MONICA - projektet

VIKTIGT!

- ☆ Använd **endast blyertspenna**
- ☆ **Fyll hela** rutan
- ☆ **Sudda noga** *(kryssa ej över)* markeringar som du vill ändra
- ☆ **Vik ej** detta papper

Markera så: ⬭ ⬭ ⬭ ⬬ ⬭

Inte så:

6732

— ARBETE —

Med arbete avses din huvudsakliga sysselsättning.

61. Hur är dina nuvarande arbetsförhållanden?

- ⬭ = fast anställning, anställning tills vidare
- ⬭ = egen företagare
- ⬭ = tillfällig anställning, vikariat, beredskapsarbete
- ⬭ = arbetar i hemmet
- ⬭ = arbetslös => gå till fråga **84**
- ⬭ = studerande => gå till fråga **82**
- ⬭ = pensionerad => gå till fråga **83**

62. Hur många timmar förvärvsarbetar du i normala fall per vecka?

- ⬭ = jag förvärvsarbetar inte
- ⬭ = mindre än 15 timmar
- ⬭ = 15-35 timmar
- ⬭ = mer än 35 timmar

63. Vad har du för arbetstider i normala fall?

- ⬭ = jag förvärvsarbetar inte
- ⬭ = fast arbetstid (t ex 7-16 eller 8-17)
- ⬭ = skiftarbete (t ex 2-skift, 3-skift, 5-skift)
- ⬭ = varierande arbetstider (ibland dagar, ibland kvällar, ibland helger)

64. Är ditt arbete fysiskt (kroppsligt) tungt?

- ⬭ = ja, ofta
- ⬭ = ja, ibland
- ⬭ = nej, sällan
- ⬭ = nej, så gott som aldrig

65. Kräver ditt arbete att du arbetar mycket fort?

- ⬭ = ja, ofta
- ⬭ = ja, ibland
- ⬭ = nej, sällan
- ⬭ = nej, så gott som aldrig

66. Är ditt arbete psykiskt påfrestande?

- ⬭ = ja, ofta
- ⬭ = ja, ibland
- ⬭ = nej, sällan
- ⬭ = nej, så gott som aldrig

67. Har du tillräckligt med tid för att hinna med arbetsuppgifterna?

- ⬭ = ja, ofta
- ⬭ = ja, ibland
- ⬭ = nej, sällan
- ⬭ = nej, så gott som aldrig

68. Förekommer det ofta motstridiga krav i ditt arbete?

- ⬭ = ja, ofta
- ⬭ = ja, ibland
- ⬭ = nej, sällan
- ⬭ = nej, så gott som aldrig

69. Får du lära dig nya saker i ditt arbete?

- ⬭ = ja, ofta
- ⬭ = ja, ibland
- ⬭ = nej, sällan
- ⬭ = nej, så gott som aldrig

WHO

MONICA - projektet

VIKTIGT!

☆ Använd **endast blyertspenna**
☆ **Fyll hela** rutan
☆ **Sudda noga** *(kryssa ej över)* markeringar som du vill ändra
☆ **Vik ej** detta papper

Markera så:

Inte så:

6732

70. Kräver ditt arbete skicklighet?

- = ja, ofta
- = ja, ibland
- = nej, sällan
- = nej, så gott som aldrig

71. Kräver ditt arbete påhittighet?

- = ja, ofta
- = ja, ibland
- = nej, sällan
- = nej, så gott som aldrig

72. Innebär ditt arbete att man gör samma sak om och om igen?

- = ja, ofta
- = ja, ibland
- = nej, sällan
- = nej, så gott som aldrig

73. Har du frihet att bestämma hur ditt arbete skall utföras?

- = ja, ofta
- = ja, ibland
- = nej, sällan
- = nej, så gott som aldrig

74. Har du frihet att bestämma vad som skall utföras i ditt arbete?

- = ja, ofta
- = ja, ibland
- = nej, sällan
- = nej, så gott som aldrig

75. Har du i vanliga fall möjlighet att tala med dina arbets-kamrater under raster, om du skulle vilja det?

- = ja, alltid
- = ja, för det mesta
- = nej, jag har inga raster
- = nej, jag har inga raster tillsammans med arbetskamrarter

76. Är ditt arbete av den karaktären att du kan lämna det ett tag om du vill tala med en arbets-kamrat?

- = ja, för det mesta
- = ja, ibland
- = bara för brådskande ärenden
- = nej, det är helt omöjligt

77. Har du, som en del i ditt arbete, ofta kontakt med dina arbetskamrater?

- = ja, ständigt kontakt med arbetskamrater i arbetet
- = ja, ibland kontakt med arbetskamrater i arbetet
- = nej, jag arbetar för det mesta ensam
- = nej, jag arbetar ständigt ensam

78. Hur ofta är du vanligen tillsammans med en eller flera av dina arbetskamrater på fritid?

- = en eller flera ggr/vecka
- = en eller ett par ggr/månad
- = en eller ett par ggr/år
- = sällan eller aldrig

79. När besökte någon av dina arbetskamrater dig senast?

- = för en till fyra veckor sedan
- = för en till tolv månader sedan
- = för mer än ett år sedan
- = har aldrig haft besök av någon arbetskamrat

WHO

MONICA - projektet

VIKTIGT!

- ☆ Använd **endast blyertspenna**
- ☆ **Fyll hela** rutan
- ☆ **Sudda noga** *(kryssa ej över)* markeringar som du vill ändra
- ☆ **Vik ej** detta papper

Markera så:

Inte så:

6732

80. Tror du att det finns risk att du blir arbetslös?
- = ja, inom den närmaste tiden
- = ja, på längre sikt
- = nej

81. Antag att du har ett arbete och blir uppsagd. Hur stora chanser tror du att du har att få ett nytt arbete inom en månad?
- = mycket stora
- = ganska stora
- = små
- = inga chanser

82. På vilken nivå studerar du?
(Denna fråga besvaras endast av studerande)
- = grundskolenivå
- = gymnasial nivå
- = eftergymnasial nivå
- = annan, vilken...

83. Har du varit arbetslös någon gång?
- = ja, för mer än 12 månader sedan
- = ja, någon gång under de senaste 12 månaderna
- = nej, aldrig => gå till fråga **85**

84. Om du varit/eller är arbetslös, hur länge har du sammanlagt varit det?
- = mindre än 3 månader
- = 3-12 månader
- = mer än 12 månader

85. Har du tvingats flytta någon gång?
- = ja, för mer än 12 månader sedan
- = ja, de senaste 12 månaderna
- = nej aldrig => gå till fråga **87**

86. Vilken var anledningen till flyttningen?
- = familjeskäl
- = arbetsmarknadsskäl
- = annat

87. Har någon eller några personer som betytt eller betyder mycket för dig, flyttat så att du inte längre kan umgås med honom/henne eller dem?
- = ja
- = nej => gå till fråga **89**

88. Om du svarat "ja" på föregående fråga, vilken var anledningen till flyttningen?
- = familjeskäl
- = arbetsmarknadsskäl
- = annat skäl

89. Är du just nu sjukskriven, har ålderpension, förtidspension, sjukpension eller sjukbidrag?
- = ja, ålderpension/sjukpension på heltid
- = ja, ålderpension/sjukpension på deltid
- = ja, förtidspension/sjukbidrag
- = ja, sjukskriven
- = nej

VIKTIGT!

- ☆ Använd **endast blyertspenna**
- ☆ **Fyll hela** rutan
- ☆ **Sudda noga** *(kryssa ej över)* markeringar som du vill ändra
- ☆ **Vik ej** detta papper

Markera så:

Inte så:

6732

90. Vilket är ditt yrke/din befattning? Om du inte förvärvsarbetar nu ska du ange yrke/sysselsättning som du tidigare huvudsakligen haft.

(Undvik allmänna yrkesbeteckningar som lärare, tjänsteman, byggnadsarbetare etc. Skriv i stället högstadielärare, kontorist, byggnadssnickare etc så att det framgår mer exakt vad du arbetar/arbetede med).

....

....

91. Beskriv kortfattat dina arbetsuppgifter, samt ange arbetsplats eller typ av företag.

....

....

....

BESVARAS AV GIFT/SAMMANBOENDE

92. Vilket är din make/makas/samboendes yrke eller befattning? *(Om han/hon inte förvärvsarbetar nu ska du ange den yrke/sysselsättning som han/hon tidigare huvudsakligen haft).*

....

....

93. Beskriv kortfattat hans/hennes arbetsuppgifter.

....

....

....

BESVARAS AV DE SOM ÄR EGNA FÖRETAGARE

94. Hur många anställda har du i ditt företag?

Antal anställda......................

BESVARAS AV JORDBRUKARE/LANTBRUKARE

95. Är ditt jordbruk/lantbruk att betrakta som ett litet eller stort jordbruk. *(Kryssa för det alternativ som stämmer bäst med din situation, samt ange hur mycket skog och åkermark du har).*

litet jordbruk. Hektar skog........ hektar åkermark........

stort jordbruk. Hektar skog........ hektar åkermark.......

WHO

MONICA - projektet

VIKTIGT!

- ☆ Använd **endast blyertspenna**
- ☆ **Fyll hela** rutan
- ☆ **Sudda noga** *(kryssa ej över)* markeringar som du vill ändra
- ☆ **Vik ej** detta papper

Markera så: ⬭ ⬭ ⬭ ⬬ ⬭

Inte så:

6732

11

— FYSISK AKTIVITET —

96. Hur mycket har du rört dig eller ansträngt dig kroppsligt på din <u>fritid</u> under det senaste året? *(Märk bara för <u>ett</u> alternativ).*

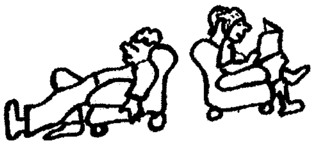

⬭ = knappast något alls

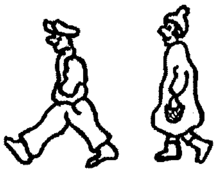

⬭ = mestadels stillasittande, ibland någon promenad eller liknande.

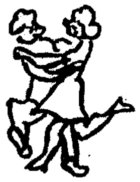

⬭ = lättare fysisk ansträngning <u>minst 2 timmar i veckan</u>, *t.ex gång och cykling (även till och från arbetet eller skolan) fiske, dans etc.*

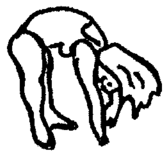

⬭ = mer ansträngande motion <u>1-2 timmar i veckan</u>, *t.ex motionslöpning, tennis, simning, badminton, motionsgymnastik.*

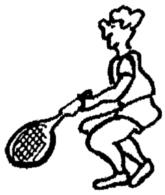

⬭ = mer ansträngande motion <u>minst 3 timmar i veckan</u>, *t.ex motionslöpning, tennis, simning, badminton, motionsgymnastik.*

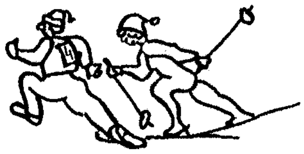

⬭ = <u>hård träning eller tävling</u> regelbundet och <u>flera gånger i veckan</u> där den fysiska ansträngningen är stor, *t.ex löpning, skidåkning, fotboll, simning*

WHO **MONICA** - projektet

VIKTIGT!

☆ Använd **endast blyertspenna**
☆ **Fyll hela** rutan
☆ **Sudda noga** *(kryssa ej över)* markeringar som du vill ändra
☆ **Vik ej** detta papper

Markera så:

Inte så:

6732

97. Hur mycket har du rört dig eller ansträngt dig kroppsligt i ditt <u>arbete</u> under det senaste året?
Denna fråga besvaras också om du arbetar i hemmet (Märk bara för <u>ett</u> alternativ)

- = jag har ålderspension/förtidspension

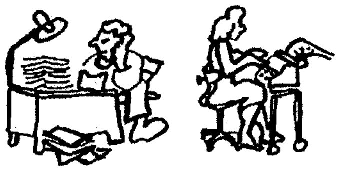

- = stillasittande arbete. *Du har övervägande stillasittande arbete och går inte mycket ut under tiden . Tex skrivbordsarbete, urmakeri och montering av lättare delar.*

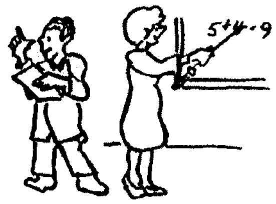

- = lätt, men något rörligt arbete. *Du har ett arbete där du går ganska mycket men inte bär eller lyfter tyngre saker. T.ex rörligt expeditionsarbete, lätt industriarbete, förmanssysslor, sådan undervisning där man går mycket, affärsbiträde, hushållsarbete.*

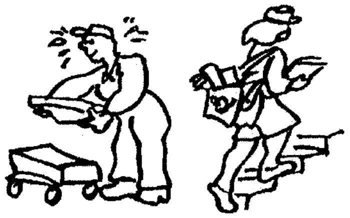

- = måttligt tungt arbete. *Du går mycket och lyfter dessutom ganska mycket eller går uppför trappor eller backar. T.ex brevbärare, arbete vid tyngre industri, byggnadssnickeri, rörläggningsarbete, vårdarbete*

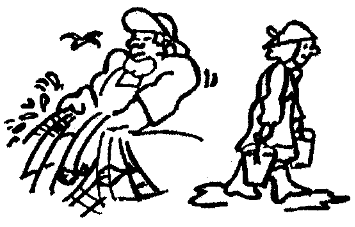

- = tungt arbete. *Du har ett tungt kroppsarbete, lyfter tunga föremål och anstränger dig mycket kroppsligt. T.ex skogsarbete, tungt lantbruksarbete, fiske med tunga redskap, byggnadsarbete.*

WHO

MONICA - projektet

VIKTIGT!

☆ Använd **endast blyertspenna**
☆ **Fyll hela** rutan
☆ **Sudda noga** *(kryssa ej över)* markeringar som du vill ändra
☆ **Vik ej** detta papper

Markera så:

Inte så:

6732

MAT och MATVANOR

98. Hur är det kaffe du dricker vanligtvis tillagat?

- = jag dricker mest kokkaffe
- = jag dricker mest bryggkaffe
- = annat (t ex snabbkaffe)
- = jag dricker mest kaffe tillagat i sk. perkulator
- = jag dricker inte kaffe => gå till fråga **100**

99. Hur många koppar kaffe dricker du i genomsnitt varje dag? *(Ange antal kaffekoppar)*

- = 1 kopp
- = 2 koppar
- = 3 koppar
- = 4 koppar
- = 5 koppar
- = 6 koppar
- = 7 koppar
- = 8 koppar
- = 9 koppar
- = 10 koppar eller mer

100. Hur ofta äter du stekt mat?

- = aldrig
- = någon gång per vecka
- = 1 gång per dag
- = 2 eller flera gånger per dag

101. Saltar du vid kokning av grönsaker, potatis mm?

- = ja
- = nej

102. Använder du extra saltat matfett?

- = ja
- = nej

103. Undviker du att äta mat med mjöl i?
(T.ex. bröd, gröt, pannkaka, palt, pizza, pasta)

- = ja
- = nej

WHO **MONICA** - projektet

VIKTIGT!

- ☆ Använd **endast blyertspenna**
- ☆ **Fyll hela** rutan
- ☆ **Sudda noga** *(kryssa ej över)* markeringar som du vill ändra
- ☆ **Vik ej** detta papper

Markera så:

Inte så:

6580

1

De följande frågorna handlar om dina personliga förhållanden och om människorna i din omgivning. Frågorna handlar både om människor som står dig nära, om vänner och om bekanta. Vissa frågor kan kanske kännas främmande, men försök att besvara dem ändå! Några frågor kan kännas väldigt lika, även om formuleringen är olika. Försök ändå att besvara dem så noggrant som möjligt.

104. Hur många människor känner du och har kontakt med, som har samma intressen som du? *Det gäller kontakter både i arbetet och på fritiden.*

- = ingen
- = 1-2 personer
- = 3-5 personer
- = 6-10 personer
- = 11-15 personer
- = mer än 15 personer

105. Hur många människor, som du känner, träffar du eller samtalar du med under en vanlig vecka? *Räkna ej med människor som du träffar tillfälligtvis och som du knappast kommer att återse, t ex kunder i en affär.*

- = ingen
- = 1-2 personer
- = 3-5 personer
- = 6-10 personer
- = 11-15 personer
- = mer än 15 personer

106. Är det ungefär lagom många människor du träffar under ditt dagliga liv? *Skulle du vilja träffa fler eller färre människor?*

- = färre
- = lagom många
- = fler

107. Hur många vänner har du som kan komma hem till dig när som helst och känna sig hemma? *De skulle inte bry sig om, om det var ostädat eller om du höll på att äta. (Nära släktingar skall inte räknas med!)*

- = ingen
- = 1-2 personer
- = 3-5 personer
- = 6-10 personer
- = 11-15 personer
- = mer än 15 personer

108. Hur många finns det, i din familj och bland dina vänner, som du kan tala öppet med utan att behöva tänka dig för?

- = ingen
- = 1-2 personer
- = 3-5 personer
- = 6-10 personer
- = 11-15 personer
- = mer än 15 personer

109. Finns det någon särskild person som du känner att du verkligen kan få stöd av?

- = nej
- = ja, men jag behöver inte det.
- = ja

110. Finns det någon särskild person som känner sig stå väldigt nära dig?

- = nej
- = är inte säker
- = ja

111. Har du någon särskild person som du kan dela dina känslor med när du känner dig lycklig? *Någon som själv skulle känner sig lycklig bara för att du är det?*

- = nej
- = ja

WHO

MONICA - projektet

VIKTIGT!

☆ Använd **endast blyertspenna**
☆ **Fyll hela** rutan
☆ **Sudda noga** *(kryssa ej över)* markeringar som du vill ändra
☆ **Vik ej** detta papper

Markera så:

Inte så:

6580

2

112. Har du någon du kan dela dina innersta känslor med och anförtro dig åt?
- = nej
- = ja

113. Händer det att någon håller om dig till tröst eller stöd?
- = nej
- = ja

114. Tror du att de där hemma eller några andra verkligen uppskattar vad du gör för dem?
- = ja
- = inte tillräckligt
- = nej, inte alls

115. Finns det människor i din omgivning som du lätt kan be om saker, t.ex. människor som du känner så väl att du kan låna verktyg eller köksgrejor?
- = nej
- = ja

116. Bortsett från dem där hemma, finns det någon som du kan vända dig till om du är i svårigheter? ***Någon som du lätt kan träffa och som du litar på och kan få verklig hjälp av när du har det besvärligt?***
- = nej — **Män** => gå till fråga **129**
- = ja — **Män** => gå till fråga **129**

Fråga **117** t.om. **128**

IFYLLES ENDAST AV KVINNOR

117. Hur många gånger har du varit gravid?
- = 0
- = 1
- = 2
- = 3
- = 4
- = 5 eller fler

118. Hur många barn har du fött?
- = 0
- = 1
- = 2
- = 3
- = 4
- = 5 eller fler

119. Har du fortfarande menstruation varje månad?
- = ja, regelbundet varje månad => gå till fråga **120**
- = ibland, men inte så regelbundet som tidigare, eller att menstruationen har upphört de senaste sex månaderna => gå till fråga **122**
- = nej, det är mer än sex månader sedan den upphörde => gå till fråga **121**
- = jag är gravid => gå till fråga **129**

120. Har du tagit preventivmedel (p-piller) eller preventivinjektion de senaste två månaderna?
- = ja => gå till fråga **129**
- = nej => gå till fråga **129**

WHO

MONICA - projektet

VIKTIGT!

- ☆ Använd **endast blyertspenna**
- ☆ **Fyll hela** rutan
- ☆ **Sudda noga** *(kryssa ej över)* markeringar som du vill ändra
- ☆ **Vik ej** detta papper

Markera så: ⬭ ⬭ ⬭ ⬬ ⬭

Inte så:

6580

3

IFYLLES ENDAST AV KVINNOR SOM ÄR I ELLER PASSERAT KLIMAKTERIET

121. Hur gammal var du när menstruationerna upphörde helt?

I__I__I

122. Har du tagit hormoner (östrogen) för dina klimakteriebesvär under den senaste månaden?

- = ja
- = nej => gå till fråga **125**

123. Vilket östrogenpreparat tar du för närvarande?

- = Cyklabil
- = Dienoestrol
- = Estraderm
- = Kliogest
- = Ovesterin
- = Progynon
- = Trisekvens
- = annat preparat...
- = jag tar östrogen men vet inte vad preparatet heter
- = vet ej

124. Hur många månader har du tagit hormoner efter klimakteriet?

I__I__I__I

125. Har du av läkare blivit avrådd från att ta hormoner efter klimakteriet

- = ja
- = nej => gå till fråga **127**
- = vet ej => gå till fråga **127**

126. Om ja, varför har du blivit avrådd?

- = tidigare hjärt-kärlsjukdom
- = har diabetes
- = har tidigare haft högt blodtryck
- = annan sjukdom eller riskfaktor
- = ingen speciell anledning
- = vet ej

127. Har du själv övervägt hormonell behandling?

- = ja => gå till fråga **129**
- = nej => gå till fråga **129**
- = vet ej

128. Om ja, varför har du avstått?

- = har hjärtsjukdom
- = har högt blodtryck
- = har diabetes
- = har eller har haft cancer
- = är rädd för hormoner
- = har inte haft anledning att ta hormoner- inga övergångsbesvär
- = ej kommit mej för eller hunnit uppsöka läkare angående detta
- = vet ej

WHO

MONICA - projektet

VIKTIGT!

- ☆ Använd **endast blyertspenna**
- ☆ **Fyll hela** rutan
- ☆ **Sudda noga** *(kryssa ej över)* markeringar som du vill ändra
- ☆ **Vik ej** detta papper

Markera så:

Inte så:

6848

1

— LIVSINSTÄLLNING —

Följande frågor avser att mäta en del av motståndskraften mot sjukdom. Frågorna är således inte inriktade på vad man har för risker att bli sjuk, utan i stället vilka tillgångar man har för att hantera påfrestningar.

Varje fråga eller påstående har sju svarsalternativ. Var snäll och markera i rutan under den siffra som närmast uttrycker vad du känner. Lämna endast ett svar på varje fråga. Din omedelbara reaktion på frågan eller påståendet är det vi söker snarare än det du kommer fram till efter lång tids funderande.

129. Har du känslan av att du inte riktigt bryr dig om vad som händer omkring dig?

mycket sällan eller aldrig — mycket ofta

1 2 3 4 5 6 7

130. Har det hänt att du blev överraskad av beteendet hos personer som som du trodde du kände väl?

har aldrig hänt — har hänt ofta

1 2 3 4 5 6 7

131. Har det hänt att du har blivit besviken på människor som du litat på?

har aldrig hänt — har hänt ofta

1 2 3 4 5 6 7

132. Hittils har ditt liv:.......

helt saknat mål och mening — genomgående haft mål och mening

1 2 3 4 5 6 7

133. Har du känslan av att du behandlas orättvist?

mycket ofta — mycket sällan eller aldrig

1 2 3 4 5 6 7

WHO

MONICA - projektet

6848	2

VIKTIGT!

- ☆ Använd **endast blyertspenna**
- ☆ **Fyll hela** rutan
- ☆ **Sudda noga** *(kryssa ej över)* markeringar som du vill ändra
- ☆ **Vik ej** detta papper

Markera så:

Inte så:

134. Har du känslan av att du hamnar i nya situationer utan att veta vad du skall göra?

mycket ofta — mycket sällan eller aldrig

1 2 3 4 5 6 7

135. Är dina dagliga sysslor en källa till.....

glädje och djup tillfredsställelse — smärta och leda

1 2 3 4 5 6 7

136. Har du mycket motstridiga känslor, tankar och idéer?

mycket ofta — mycket sällan eller aldrig

1 2 3 4 5 6 7

137. Händer det att du har känslor inom dig som du helst inte vill känna?

mycket ofta — mycket sällan eller aldrig

1 2 3 4 5 6 7

138. Många människor - även de med stark självkänsla - kan känna sig helt misslyckade i vissa situationer. Hur ofta har du känt det så?

aldrig — mycket ofta

1 2 3 4 5 6 7

139. När någonting har hänt, har du i allmänhet upptäckt att du...

över- eller undervärderar betydelsen av vad som hände — såg saker i dess rätta proportioner

1 2 3 4 5 6 7

140. Hur ofta känner du att det inte finns någon mening med dina dagliga sysslor?

mycket ofta — mycket sällan eller aldrig

1 2 3 4 5 6 7

141. Hur ofta har du känslor som du är osäker på att kunna behärska?

mycket ofta — mycket sällan eller aldrig

1 2 3 4 5 6 7
