# Supplementary material for: Time trends of comparative self-rated health in adults aged 25-34 in the Northern Sweden MONICA study, 1990-2014
Source: PLoS One. 2017 Nov 20;12(11):e0187896. doi: 10.1371/journal.pone.0187896 (PMC5695772; doi:10.1371/journal.pone.0187896)
Supplement: S3 Text — (PDF) [file pone.0187896.s003.pdf]

monica centre WHO/OMS northern sweden

MONICA-nummer:

Namn:

Adress:

Personnummer:

Ny Matenkät |__|__|__|__|__|__|

VÄRLDSHÄLSOORGANISATIONENS

KAMPANJ MOT

HJÄRT- och KÄRLSJUKDOMAR

1999

FRÅGEFORMULÄR DEL 1

INFORMATION

Det frågeformulär som vi nu ber dig att besvara, innehåller frågor rörande bl.a. hälsa, arbete, fysisk aktivitet, rök-, kost- och motionsvanor. Alla uppgifter som du lämnar kommer att behandlas konfidentiellt.
Resultaten kommer att sammanställas på sådant sätt att det inte går att identifiera enskilda individer.

Vi ber dig att noga läsa igenom frågorna och svarsalternativen och att du försöker besvara alla frågor.

Om det är något du funderar över angående frågeformulären eller undersökningen, kontakta gärna någon av oss. Du når oss säkrast på telefon på förmiddagarna.

Om vi skulle få problem med att tolka något av dina ifyllda svar, skulle vi vara mycket tacksamma om vi får ringa dig för att reda ut det som är oklart. Vill du ange det telefonnummer där vi lättast kan nå dig (helst dagtid).

Mitt telefonnummer är ....
riktnummer telefonnummer

Jag träffas säkrast ....

Gunborg Rönnberg

MONICA-projektet
Medicinska kliniken
Norrlands Universitetssjukhus
901 85 UMEÅ
Tel: 090 - 785 25 18

Vivan Lundberg /Jan-Ola Gustafsson

MONICA-projektet
Kalix sjukhus
952 82 KALIX
Tel: 0923 - 131 33 (kl 08.00-10.00)
Tel: 0923 - 762 41 (efter kl 10.00)

Endast ett X på varje fråga där inte annat anges

Frågor rörande CIVILSTÅND och BOENDE

1. **Vilket är ditt nuvarande civilstånd?**
   I__I = ogift
   I__I = gift eller sammanboende
   I__I = skild eller separerad
   I__I = änka/änkling
   I__I = annat____________________________

2. **Vilka personer sammanbor du med ?**
   I__I = vuxen (make, maka, sambo)
   I__I = barn, ange antal I__I__I
   I__I = vuxen och barn, ange antal I__I__I
   I__I = annan
   I__I = bor helt ensam

3. **Var bor du?**
   I__I = större tätort - Umeå, Skellefteå, Luleå, Boden, Piteå, Kiruna, orter med mer än 15 000 invånare
   I__I = annan tätort med mer än 1 000 invånare
   I__I = ort med mindre än 1 000 invånare

4. **I vilket land är du född?**
   I__I = Sverige ⟹ gå till fråga 5
   I__I = Finland ⟹ gå till fråga 6
   I__I = annat land ⟹ gå till fråga 6

5. **Var är du född?** *(Ange vilken kyrkoförsamling eller ort du tillhörde vid födseln)?*

....

församling/ort län

6. **Vilken är den högsta utbildningsnivå som du har avslutat?**

I__I = folkskola
I__I = grundskola
I__I = folkhögskola motsvarande grundskolekompetens
I__I = yrkesskola
I__I = realskola
I__I = flickskola
I__I = fackskola
I__I = gymnasieskola
I__I = folkhögskola motsvarande gymnasiekompetens
I__I = högskola eller universitet

7. **Hur många år har du gått i skola eller ägnat dig åt studier på heltid? (Ange antal år från första klass och framåt)**

I__I__I

Frågor rörande TOBAKSVANOR

8. **Röker du cigaretter för närvarande?**

I__I = ja, regelbundet (1 cigarett eller mer per dag) ⟹ gå till fråga **9**
I__I = nej ⟹ gå till fråga **12**
I__I = ibland (mindre än 1 cigarett per dag) ⟹ gå till fråga **10**

9. **Ungefär hur många cigaretter röker du i genomsnitt per dag?**
(Ange antal cigaretter/dag)

I__I__I__I ⟹ gå till fråga **16**

10. **Hur många dagar i veckan röker du cigaretter?**

I__I = vanligtvis en dag eller mindre
I__I = vanligtvis 2 till 4 dagar
I__I = nästan varje dag

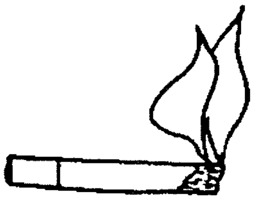

11. **Ungefär hur många cigaretter röker du i genomsnitt per vecka?**

I__I__I__I

12. **Har du någonsin rökt cigaretter regelbundet tidigare?**

I__I = ja, regelbundet ⟹ gå till fråga **13**
I__I = nej ⟹ gå till fråga **18**

13. **När slutade du att röka cigaretter regelbundet?** *(Ange årtal)*

    19 I__I__I

14. **Om du slutade röka under det senaste året, när slutade du?**

    I__I = för mindre än en månad sedan

    I__I = för 1-6 månader sedan

    I__I = för 7-12 månader sedan

15. **Vilken är den främsta orsaken till att du slutat röka?**
    *(Ange endast **ett** alternativ)*

    I__I = av hälsoskäl, på eget initiativ

    I__I = på inrådan av läkare/sjukvårdspersonal

    I__I = pga annan information/upplysning

    I__I = pga tryck från kamrater/familjemedlemmar

    I__I = av andra skäl

16. **Vilket är det högsta antal cigaretter/dag, som du rökt under en så lång period som ett år?**

    I__I__I__I

17. **Hur gammal var du när du började röka cigaretter?** ***(Ange ålder i år)***

    I__I__I år

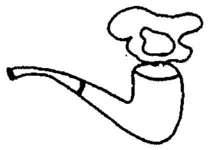

18. **Hur du någonsin rökt <u>pipa?</u>**

    I__I = ja, för närvarande regelbundet ⇒ gå till fråga **19**

    I__I = nej ⇒ gå till fråga **20**

    I__I = jag röker pipa ibland (mindre än 1 gång/dag) ⇒ gå till fråga **19**

    I__I = tidigare, men inte nu ⇒ gå till fråga **20**

19. **Hur många gram tobak röker du per vecka?**
    Ett paket piptobak väger som regel 50 gram *(Ange antal gram/vecka)*

    I__I__I__I

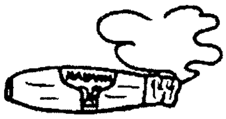

20. **Hur du någonsin rökt <u>cigarrer eller cigariller</u>**

    I__I = ja, för närvarande regelbundet ⇒ gå till fråga **21**

    I__I = nej ⇒ gå till fråga **22**

    I__I = jag röker cigarrer/cigariller ibland (mindre än 1/dag) ⇒ gå till fråga **21**

    I__I = tidigare, men inte nu ⇒ gå till fråga **22**

21. **Hur många cigarrer/cigariller röker du per vecka?**
(Ange antal cigarrer/cigariller per vecka)

I__I__I__I

OBS! Fråga 22 skall endast fyllas i av icke-rökare och de som röker ibland

22. **Hur många timmar i medeltal per dag, är du i närheten av andra människors tobaksrök?**
(Ange antal timmar/dag du inandas eller känner lukten av tobaksrök)

I__I__I

23. **Har du någonsin använt snus?**

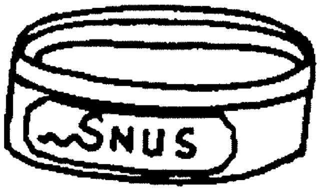

I__I = ja, snusade tidigare men inte nu
I__I = ja, snusar mindre än 2 dosor per vecka
I__I = ja, snusar 2 - 4 dosor per vecka
I__I = ja, snusar mer än 4 men mindre än 7 dosor per vecka
I__I = ja, snusar 7 dosor eller fler per vecka
I__I = nej ⟹ gå till fråga **26**

24. **Hur många år har du använt snus?** *(Ange antal år)*

I__I__I

25. **Började du att snusa i samband med att du slutade röka?**

I__I = ja
I__I = nej
I__I = jag både röker och snusar

26. **Använder du dagligen andra nikotin produkter?**
(Gäller ej cigarett, cigarr, pipa eller snus)

I__I = ja, tuggtobak
I__I = ja, nikotintuggummi
I__I = ja, nikotinplåster
I__I = annat ....
I__I = nej

Frågor rörande HÄLSA

27. **Har du vid något tillfälle av läkare eller annan sjukvårds-personal fått besked om att du har högt blodtryck?**

I__I = ja

I__I = nej ⟹ gå till fråga **29**

28. **Har du under de senaste 2 veckorna tagit läkemedel mot förhöjt blodtryck?**

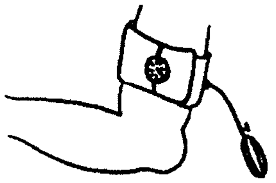

I__I = ja

I__I = nej

I__I = osäker på om den medicin jag äter är mot högt blodtryck

29. **Har du fått ditt blodtryck mätt under det senaste året?**

I__I = ja

I__I = nej

30. **Har eller hade någon av dina föräldrar behandling för högt blodtryck?**

I__I = ja, min pappa

I__I = ja, min mamma

I__I = ja, båda föräldrarna

I__I = nej, ingen av föräldrarna

I__I = vet ej

31. **Har du vid något tillfälle av läkare eller annan sjukvårdspersonal fått besked om att du har förhöjda kolesterol-/blodfettvärden?**

I__I = ja

I__I = nej ⟹ gå till fråga **34**

32. **Har läkare eller annan sjukvårdspersonal föreskrivet en särskild diet till dig för att sänka dina kolesterol-/blodfettnivåer?**

I__I = ja

I__I = nej

I__I = jag håller en föreskriven diet, men vet ej om den är till för att sänka mina kolesterol-/blodfettnivåer

33. **Har du under de senaste två veckorna tagit medicin, föreskriven av läkare, för att sänka kolesterol-/blodfettvärdet?**

I__I = ja

I__I = nej

I__I = vet ej om medicinen jag äter är för att sänka kolesterolet

34. **Har du fått ditt kolesterol/blodfett mätt under det senaste året?**

I__I = ja

I__I = nej

35. **Har du under de senaste två veckorna tagit acetylsalicylika t ex Albyl, Aspirin, Bamyl, Bamycor, Dispril, Trombyl, Magnecyl för att förebygga eller behandla hjärtsjukdom?**

I__I = ja

I__I = nej

I__I = nej, men jag använder acetylsalicylika regelbundet för att behandla annan sjukdom än hjärtsjukdom

36. **Har du under de senaste två veckorna tagit acetylsalicylika t ex Albyl, Aspirin, Bamyl, Bamycor, Dispril, Trombyl, Magnecyl för att förebygga eller behandla slaganfall (propp i hjärnan)?**

I__I = ja

I__I = nej, använder inte acetylsalicylika regelbundet.

I__I = nej, men jag använder acetylsalicylika regelbundet för att behandla annan sjukdom än slaganfall

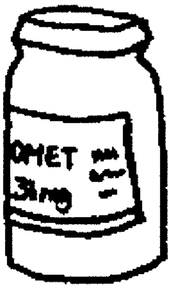

37. **Har du under de senaste 14 dagarna använt något läkemedel som ordinerats av läkare?**

I__I = ja

I__I = nej

38. **Om du svarat "ja" i så fall vilken eller vilka mediciner?**

1______________________ 4______________________

2______________________ 5______________________

3______________________ 6______________________

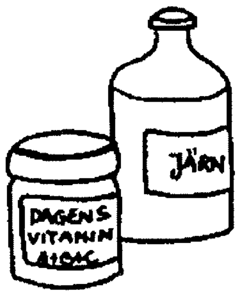

39. **Har du under de senaste 14 dagarna använt någon annan medicin?** (T.ex. naturmedicin, vitamin- eller järnpreparat, förebyggande medicin).

I__I = ja

I__I = nej

40. **Om du svarat "ja" i så fall vilken eller vilka mediciner?**

1______________________ 2______________________

3______________________ 4______________________

41. **Får du smärtor - stickningar - ont i bröstet när du går uppför backar eller trappor, eller när du går fort på plan mark?**

I__I = ja

I__I = nej

42. **Får du smärtor - stickningar - ont i bröstet när du går i vanlig takt på plan mark?**

I__I = ja

I__I = nej

Om** du svarat **nej** på både fråga 41 och 42* ⟹ *gå till fråga **47

43. **Om du får smärtor eller obehag i bröstet i samband med att du rör dig, brukar du då?......**

I__I = stanna

I__I = sakta ner farten

I__I = fortsätta i samma takt

44. **Om du stannar eller saktar ner, försvinner smärtorna då?**

I__I = ja

I__I = nej

45. **Om de försvinner, hur snart försvinner de?**

I__I = efter mindre än 10 minuter

I__I = efter mer än 10 minuter

46. **VAR BRUKAR DU KÄNNA DESSA SMÄRTOR ELLER OBEHAG?**

(*Markera med X på figuren där du känner smärta eller obehag. Om smärta eller obehag känns ut i armarna eller upp mot halsen, markera med X även där!)*

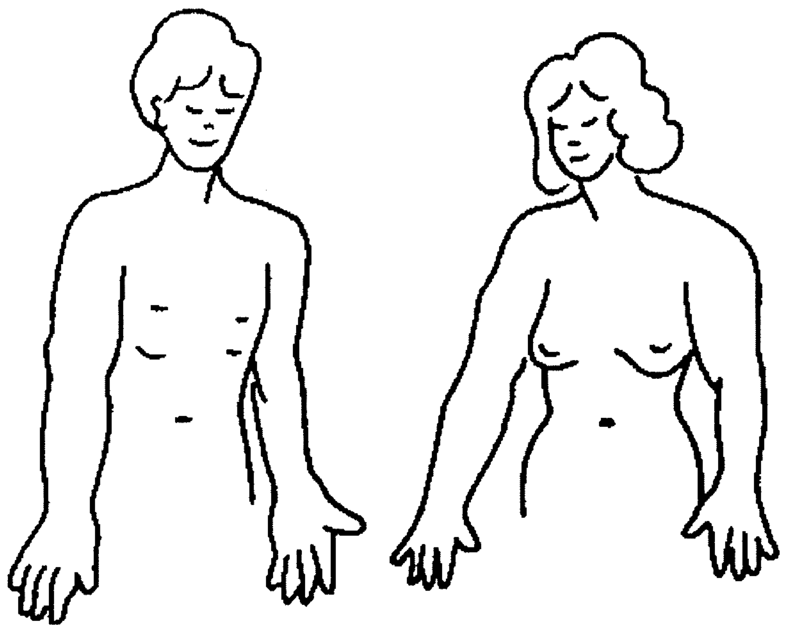

47. **Har du någon gång haft svår smärta i bröstet som varat i en halvtimme eller mer?**

I__I = ja

I__I = nej

48. **Har du legat på sjukhus för säker hjärtinfarkt (propp i hjärtat)?**

I__I = ja, ange årtal 19 I__I__I och sjukhus________________________

I__I = nej

I__I = vet ej om det var en säker hjärtinfarkt

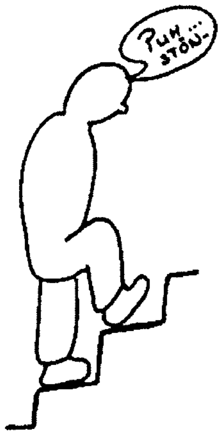

49. **Brukar du få smärtor i vaderna, när du går i uppförsbackar, trappor eller på plan mark?**

I__I = ja

I__I = nej

50. **Blir du andfådd av att gå två trappor upp eller motsvarande i samma takt som jämnåriga?**

I__I = ja

I__I = nej

51. **Har du haft slaganfall (hjärnblödning eller propp i hjärnan)?**

I__I = ja, ange årtal 19 I__I__I och sjukhus ________________________

I__I = nej

52. **Har du diabetes /sockersjuka?**

I__I = ja

I__I = nej ⟹ gå till fråga **55**

53. **Hur behandlar du din diabetes/sockersjuka?**

I__I = insulin

I__I = tabletter

I__I = enbart kost

I__I = ingetdera

54. **Hur gammal var du när du fick diabetes/sockersjuka?**

I__I__I år

55. **Har eller hade någon av dina föräldrar diabetes/sockersjuka?**

I__I= ja , min pappa

I__I= ja, min mamma

I__I= båda mina föräldrar

I__I= nej, ingen av mina föräldrar

I__I= vet ej

56. Har du mångåriga magbesvär?

I__I = ja

I__I = nej

Flera alternativ är möjliga!

57. Finns det någon i din släkt som avlidit i hjärtinfarkt före 65 års ålder? Med släkt menas i detta fall: Föräldrar, syskon, fastrar, farbröder, mostrar och morbröder.

I__I = ja, min mamma Hon var då........... år

I__I = ja, min pappa Han var då........... år

I__I = ja, något/några syskon Han/hon var då........... år

I__I = ja, någon/några av fastrar, farbröder, mostrar och morbröder. Han/hon var då........... år

I__I = nej

I__I = vet ej

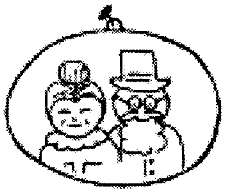

58. Finns det någon i din släkt som avlidit i slaganfall (propp eller blödning i hjärnan) före 65 års ålder? Med släkt menas i detta fall: Föräldrar, syskon, fastrar, farbröder, mostrar och morbröder.

I__I = ja, min mamma Hon var då........... år

I__I = ja, min pappa Han var då........... år

I__I = ja, något/några syskon Han/hon var då........... år

I__I = ja, någon/några av fastrar, farbröder, mostrar och morbröder. Han/hon var då........... år

I__I = nej

I__I = vet ej

Endast ett X på varje fråga där inte annat anges

59. Snarkar du när du sover?

I__I = ja, alltid

I__I = ja, nästan alltid

I__I = ja, ibland

I__I = nej, nästan aldrig

I__I = nej, aldrig

I__I = vet inte om jag snarkar

60. **Besväras du av trötthet under dagen?**

I__I = ja, alltid

I__I = ja, nästan alltid

I__I = ja, ibland

I__I = nej, nästan aldrig

I__I = nej, aldrig

61. **Har din make/maka/sambo märkt att du har andningsuppehåll under sömn?**

I__I = ja, i stort sett varje natt

I__I = ja, ofta

I__I = ja, ibland

I__I = nej, nästan aldrig

I__I = nej, aldrig

I__I = vet ej

62. **Hur många mil kör du bil per år?**

Ange antal mil per år: I__I__I__I__I

I__I = jag har inget körkort

I__I = jag kör aldrig bil

63. **Har du varit med om någon olycka som föranlett läkarbesök på grund av nackskada eller skallskada?** *Flera alternativ är möjliga*

I__I = ja, whiplash- (pisksnärts-) skada

I__I = ja, annan nackskada

I__I = ja, skallskada

I__I = nej

64. **Kontrolleras eller behandlas du av läkare för reumatiska led- eller muskelbesvär i form av smärtor/värk, stelhet?** *Flera alternativ är möjliga*

I__I = ja, muskelbesvär

I__I = ja, ledbesvär

I__I = nej

65. **Har du några besvär i form av smärta/värk, stelhet i *nacke* och/eller *ländrygg*?**

I__I = ja, men **inte** orsakad av olycka (bil, fall etc)

I__I = ja, orsakad av olycka (bil, fall etc)

I__I = nej ⟹ gå till fråga **70**

66. **Om du har nacksmärtor och /eller värk, stelhet, hur länge har du haft besvär?**

I__I = senaste veckan

I__I = senaste 6 månaderna

I__I = mer än 6 månader

67. **Om du har nacksmärtor och /eller värk, stelhet, hur ofta har du besvär?**

I__I = ständiga besvär

I__I = några gånger i månaden

I__I = enstaka gånger per år

68. **Om du har ländryggssmärta och/eller värk, stelhet, hur länge har du haft besvär?**

I__I = senaste veckan

I__I = senaste 6 månaderna

I__I = mer än 6 månader

69. **Om du har ländryggssmärta och/eller värk, stelhet, hur ofta har du besvär?**

I__I = ständiga besvär

I__I = några gånger i månaden

I__I = enstaka gånger per år

70. **Hur är det kaffe du dricker vanligtvis tillagat?**

I__I = jag dricker mest kokkaffe

I__I = jag dricker mest bryggkaffe

I__I = annat (t ex snabbkaffe)

I__I = jag dricker mest kaffe tillagat i s.k. perkulator

I__I = jag dricker inte kaffe ⟹ gå till fråga **72**

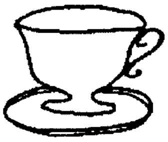

71. **Hur många koppar kaffe dricker du i genomsnitt varje dag?**
(Ange antal kaffekoppar)

I__I__I

Frågor om ARBETE

Med arbete avses din huvudsakliga sysselsättning.
OBS: Ange endast ett alternativ!

72. **Hur är dina nuvarande arbetsförhållanden?**

I__I = fast anställning, anställning tills vidare
I__I = egen företagare
I__I = tillfällig anställning, vikariat, beredskapsarbete
I__I = arbetar i hemmet
I__I = arbetslös ⟹ gå till fråga **95**
I__I = studerande ⟹ gå till fråga **93**
I__I = pensionerad ⟹ gå till fråga **94**

73. **Hur många timmar förvärvsarbetar du i normala fall per vecka?**

I__I = jag förvärvsarbetar inte
I__I = mindre än 15 timmar
I__I = 15-35 timmar
I__I = mer än 35 timmar

74. **Vad har du för arbetstider i normala fall?**

I__I = jag förvärvsarbetar inte
I__I = fast arbetstid (t ex 7-16 eller 8-17)
I__I = skiftarbete (t ex 2-skift, 3-skift, 5-skift)
I__I = varierande arbetstider (ibland dagar, ibland kvällar, ibland helger)

75. **Är ditt arbete fysiskt (kroppsligt) tungt?**

I__I = ja, ofta
I__I = ja, ibland
I__I = nej, sällan
I__I = nej, så gott som aldrig

76. **Kräver ditt arbete att du arbetar mycket fort?**

I__I = ja, ofta
I__I = ja, ibland
I__I = nej, sällan
I__I = nej, så gott som aldrig

77. **Är ditt arbete psykiskt påfrestande?**
I__I = ja, ofta
I__I = ja, ibland
I__I = nej, sällan
I__I = nej, så gott som aldrig

78. **Har du tillräckligt med tid för att hinna med arbetsuppgifterna?**
I__I = ja, ofta
I__I = ja, ibland
I__I = nej, sällan
I__I = nej, så gott som aldrig

79. **Förekommer det ofta motstridiga krav i ditt arbete?**
I__I = ja, ofta
I__I = ja, ibland
I__I = nej, sällan
I__I = nej, så gott som aldrig

80. **Får du lära dig nya saker i ditt arbete?**
I__I = ja, ofta
I__I = ja, ibland
I__I = nej, sällan
I__I = nej, så gott som aldrig

81. **Kräver ditt arbete skicklighet?**
I__I = ja, ofta
I__I = ja, ibland
I__I = nej, sällan
I__I = nej, så gott som aldrig

82. **Kräver ditt arbete påhittighet?**
I__I = ja, ofta
I__I = ja, ibland
I__I = nej, sällan
I__I = nej, så gott som aldrig

83. **Innebär ditt arbete att man gör samma sak om och om igen?**
I__I = ja, ofta
I__I = ja, ibland
I__I = nej, sällan
I__I = nej, så gott som aldrig

84. Har du frihet att bestämma hur ditt arbete skall utföras?

I__I = ja, ofta
I__I = ja, ibland
I__I = nej, sällan
I__I = nej, så gott som aldrig

85. Har du frihet att bestämma vad som skall utföras i ditt arbete?

I__I = ja, ofta
I__I = ja, ibland
I__I = nej, sällan
I__I = nej, så gott som aldrig

86. Har du i vanliga fall möjlighet att tala med dina arbetskamrater under raster, om du skulle vilja det?

I__I = ja, alltid
I__I = ja, för det mesta
I__I = nej, jag har inga raster
I__I = nej, jag har inga raster tillsammans med arbetskamrater

87. Är ditt arbete av den karaktären att du kan lämna det ett tag om du vill tala med en arbetskamrat?

I__I = ja, för det mesta
I__I = ja, ibland
I__I = bara för brådskande ärenden
I__I = nej, det är helt omöjligt

88. Har du, som en del i ditt arbete, ofta kontakt med dina arbetskamrater?

I__I = ja, ständigt kontakt med arbetskamrater i arbetet
I__I = ja, ibland kontakt med arbetskamrater i arbetet
I__I = nej, jag arbetar för det mesta ensam
I__I = nej, jag arbetar ständigt ensam

89. Hur ofta är du vanligen tillsammans med en eller flera av dina arbets kamrater på fritiden?

I__I = en eller flera ggr/vecka
I__I = en eller ett par ggr/månad
I__I = en eller ett par ggr/år
I__I = sällan eller aldrig

90. När besökte någon av dina arbetskamrater dig senast?

I__I = för en till fyra veckor sedan

I__I = för en till tolv månader sedan

I__I = för mer än ett år sedan

I__I = har aldrig haft besök av någon arbetskamrat

91. Tror du att det finns risk att du blir arbetslös?

I__I = ja, inom den närmaste tiden

I__I = ja, på längre sikt

I__I = nej

92. Antag att du har ett arbete och blir uppsagd. Hur stora chanser tror du att du har att få ett nytt arbete inom en månad?

I__I = mycket stora

I__I = ganska stora

I__I = små

I__I = inga chanser

93. På vilken nivå studerar du? *(Denna fråga besvaras endast av studerande)*

I__I = grundskolenivå

I__I = gymnasial nivå

I__I = eftergymnasial nivå

I__I = annan, vilken ______________________________

94. Har du varit arbetslös någon gång?

I__I = ja, för mer än 12 månader sedan

I__I = ja, någon gång under de senaste 12 månaderna

I__I = nej, aldrig ⇨ gå till fråga **96**

95. Om du varit/eller är arbetslös, hur länge har du sammanlagt varit det?

I__I = mindre än 3 månader

I__I = 3-12 månader

I__I = mer än 12 månader

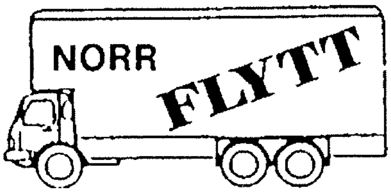

96. Har du tvingats flytta någon gång?

I__I = ja, för mer än 12 månader sedan

I__I = ja, de senaste 12 månaderna

I__I = nej aldrig ⇨ gå till fråga **98**

97. **Vilken var anledningen till flyttningen?**

I__I = familjeskäl

I__I = arbetsmarknadsskäl

I__I = annat

98. **Har någon eller några personer som betytt eller betyder mycket för dig, flyttat så att du inte längre kan umgås med honom/henne eller dem?**

I__I = ja

I__I = nej ⟹ gå till fråga **100**

99. **Om du svarat "ja" på föregående fråga, vilken var anledningen till flyttningen?**

I__I = familjeskäl

I__I = arbetsmarknadsskäl

I__I = annat skäl

100. **Är du just nu sjukskriven, har ålderspension, har förtidspension, har sjukpension eller sjukbidrag?**

I__I = ja, ålderspension/sjukpension på heltid

I__I = ja, ålderspension/sjukpension på deltid

I__I = ja, förtidspension/sjukbidrag

I__I = ja, sjukskriven

I__I = nej ⟹ gå till fråga **103**

101. **Om du just nu är sjukskriven, har förtidspension, har sjukpension eller sjukbidrag, är då orsaken till detta:** ***(Flera alternativ möjliga)***

I__I = Ja, nacksmärtor –värk, stelhet

I__I = Ja, bröstryggsmärtor

I__I = Ja, ländryggssmärta med stelhet

I__I =Ja, ländryggssmärta med smärtutstrålning och domning i underben eller fot

I__I = annan orsak

102. **Om du är sjukskriven, har förtidspension, har sjukpension eller sjukbidrag på grund *av nack-* eller *ländryggsbesvär* är då dessa orsakade av:** ***(Flera alternativ möjliga)***

I__I = Ja, trafikolycka med bil

I__I = Ja, annan olycka

I__I = Ja, arbetsskada

I__I = Ja, annan orsak

103. Vilket är ditt yrke/din befattning? Om du inte förvärvsarbetar nu ska du ange yrke/sysselsättning som du tidigare huvudsakligen haft.

(Undvik allmänna yrkesbeteckningar som lärare, tjänsteman, byggnads-arbetare etc. Skriv i stället högstadielärare, kontorist, byggnadssnickare etc så att det framgår mer exakt vad du arbetar/arbetade med).

____

____

104. Beskriv kortfattat dina arbetsuppgifter, samt ange arbetsplats eller typ av företag.

____

____

Besvaras av gifta/samboende

105. Vilket är din make/makas/samboendes yrke eller befattning?
Om han/hon inte förvärvsarbetar nu ska du ange den yrke/sysselsättning som han/hon tidigare huvudsakligen haft).

____

____

106. Beskriv kortfattat hans/hennes arbetsuppgifter.

____

____

Besvaras av de som är egna företagare

107. Hur många anställda har du i ditt företag?

Antal anställda ________________

Besvaras av jordbrukare/lantbrukare

108. Är ditt jordbruk/lantbruk att betrakta som ett litet eller stort jordbruk.
(Kryssa för det alternativ som stämmer bäst med din situation, samt ange hur mycket skog och åkermark du har).

I__I litet jordbruk. Hektar skog __________ och hektar åkermark__________

I__I stort jordbruk. Hektar skog __________ och hektar åkermark__________

Frågor om FYSISK AKTIVITET

Sätt bara X i ett av alternativen

109. Hur mycket har du rört dig eller ansträngt dig kroppsligt på din fritid under det senaste året?

I__I = **knappast något alls.**

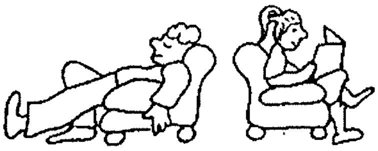

I__I = **mestadels stillasittande, ibland någon promenad eller liknande.**

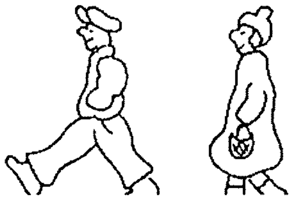

I__I = **lättare fysisk ansträngning minst 2 timmar i veckan,**

t.ex. gång och cykling (även till och från arbetet eller skolan) fiske, dans etc.

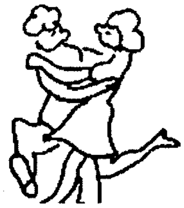

I__I = **mer ansträngande motion 1-2 timmar i veckan,**

t.ex. motionslöpning, tennis, simning, badminton, motionsgymnastik.

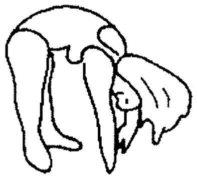

I__I = **mer ansträngande motion minst 3 timmar i veckan,**

t.ex. motionslöpning, tennis, simning, badminton, motionsgymnastik

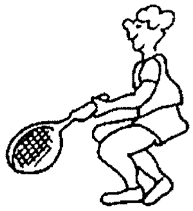

I__I = **hård träning eller tävling regelbundet och flera gånger I veckan** där den fysiska ansträngningen är stor,

t.ex. löpning, skidåkning, fotboll,simning

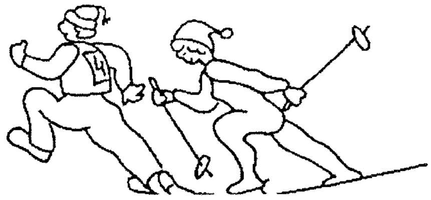

Sätt bara X i ett av alternativen

110. Hur mycket har du rört dig eller ansträngt dig kroppsligt i ditt <u>arbete</u> under det senaste året? *Denna fråga besvaras också om du arbetar I hemmet.*

I__I = **jag har ålderspension/förtidspension**

I__I = **stillasittande arbete** *Du har övervägande stillasittande arbete och går inte mycket ut under tiden .*

T.ex. skrivbordsarbete, urmakeri och montering av lättare delar.

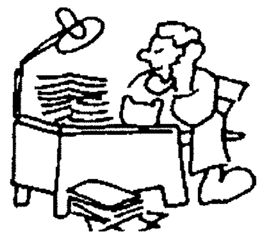
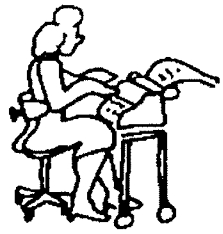

I__I = **lätt, men något rörligt arbete.** *Du har ett arbete där du går ganska mycket men inte bär eller lyfter yngre saker.*

T.ex. rörligt expeditionsarbete, lätt industriarbete, förmanssysslor, sådan undervisning där man går mycket, affärsbiträde, hushållsarbete.

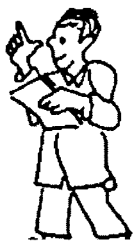
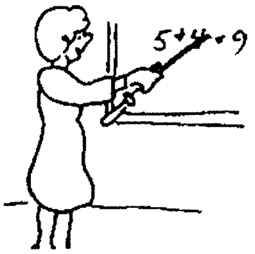

I__I = **måttligt tungt arbete.** *Du går mycket och lyfter dessutom ganska mycket eller går uppför trappor eller backar.*

T.ex. brevbärare, arbete vid tyngre industri, byggnadssnickeri, rörläggningsarbete, vårdarbete

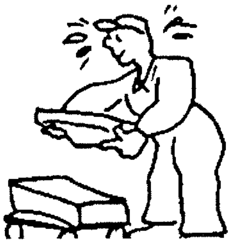
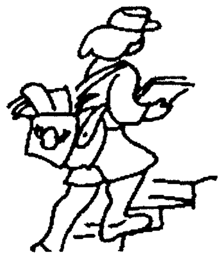

I__I = **tungt arbete.** *Du har ett tungt kroppsarbete, lyfter tunga föremål och anstränger dig mycket kroppsligt.*

T.ex. skogsarbete, tungt lantbruksarbete, fiske med tunga redskap, byggnadsarbete.

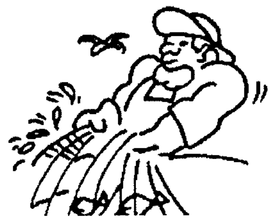
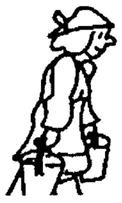

Ifylles endast av kvinnor
Männen kan sluta att fylla i formuläret här.

111. Hur många gånger har du varit gravid

I__I__I gånger

112. Hur många barn har du fött?

I__I__I barn

113. Om du fött barn, hade du då graviditetsdiabetes (dvs socker i urinen eller förhöjt blodsocker) under graviditeten?

I__I = ja

I__I = nej

I__I = har inga barn

I__I = vet ej

Om **ja** ange födelsevikt på barnet/barnen

I__I__I__I__I g I__I__I__I__I g I__I__I__I__I g

I__I__I__I__I g I__I__I__I__I g I__I__I__I__I g

114. Har du fortfarande menstruation varje månad?

I__I = ja, regelbundet varje månad ⇨ gå till fråga **115**

I__I = ibland, men inte så regelbundet som tidigare, eller att menstruationen har upphört de senaste sex månaderna ⇨ gå till fråga **117**

I__I = nej, det är mer än sex månader sedan den upphörde ⇨ gå till fråga **116**

I__I = jag är gravid ⇨ gå till fråga **115**

115. Har du tagit preventivmedel (p-piller) eller preventivinjektion de senaste två månaderna

I__I = ja

I__I = nej

Menstruerar du fortfarande eller om du är gravid så kan du sluta fylla i enkäten här. Övriga kvinnor går till nästa fråga.

Ifylles endast av kvinnor som är i eller har passerat klimakteriet
Övriga kvinnor slutar här.

116. Hur gammal var du när menstruationerna upphörde helt?

I__I__I

117. Har du tagit hormontabletter (östrogen) för dina klimakteriebesvär under den senaste månaden?

I__I = ja

I__I = nej ⟹ gå till fråga **120**

118. Vilket östrogenpreparat tar du för närvarande?

I__I = Cyklabil

I__I = Divegel

I__I = Estracomb

I__I = Kliogest

I__I = Trivina

I__I = Progynon

I__I = Trisekvens

I__I = Annat preparat..

I__I = jag tar östrogen men vet inte vad preparatet heter

I__I = vet ej

119. Hur många månader har du tagit hormoner efter klimakteriet?

I__I__I__I

120. Har du av läkare blivit avrådd från att ta hormoner efter klimakteriet?

I__I = ja

I__I = nej

I__I = vet ej

121. Om ja, varför har du blivit avrådd?

I__I = tidigare hjärt-kärlsjukdom

I__I = har diabetes

I__I = har tidigare haft högt blodtryck

I__I = annan sjukdom eller riskfaktor

I__I = ingen speciell anledning

I__I = vet ej

122. Har du själv övervägt hormonell behandling?

I__I= ja

I__I = nej

123. Om ja, varför har du avstått?

I__I = har hjärtsjukdom

I__I = har högt blodtryck

I__I = har diabetes

I__I = har eller har haft cancer

I__I = är rädd för hormoner

I__I = har inte haft anledning att ta hormoner- inga övergångsbesvär

I__I = ej kommit mig för eller hunnit uppsöka läkare angående detta

I__I = vet ej

TACK FÖR DIN MEDVERKAN !

Var vänlig tag med detta formulär när du går till undersökningen

VÄRLDSHÄLSOORGANISATIONENS

KAMPANJ MOT

HJÄRT- och KÄRLSJUKDOMAR

1999

FRÅGEFORMULÄR DEL 2

Frågor rörande LIVSKVALITET OCH SOCIALT STÖD

Besvara följande frågor genom att ange det svarsalternativ som bäst stämmer överens med din personliga situation/uppfattning

124. Hur tycker du ditt allmänna hälsotillstånd varit det senaste året?

I__I = mycket gott

I__I = ganska gott

I__I = någorlunda

I__I = tämligen dåligt

I__I = dåligt

125. Hur bedömer du ditt allmänna hälsotillstånd jämfört med andra personer i din egen ålder?

I__I = det är bättre

I__I = det är sämre

I__I = ungefär likadant

De följande frågorna handlar om dina personliga förhållanden och om människorna i din omgivning. Frågorna handlar både om människor som står dig mycket nära, om vänner och om bekanta. Vissa frågor kan kanske kännas främmande, men försök att besvara dem ändå! Några frågor kan kännas väldigt lika, även om formuleringen är olika. Försök ändå att besvara dem så noggrant som möjligt.

Markera hur tillfredsställd du är med din situation i olika avseenden. Sätt ett kryss i rutan för den siffra som bäst anger hur du känner dig nu.

126. Hem- och familjesituation

mycket dåligt	I__I	I__I	I__I	I__I	I__I	I__I	I__I	alldeles utmärkt kunde
	1	2	3	4	5	6	7	inte vara bättre

127. Bostad

mycket dåligt	I__I	I__I	I__I	I__I	I__I	I__I	I__I	alldeles utmärkt kunde
	1	2	3	4	5	6	7	inte vara bättre

128. Arbetssituation

mycket dåligt	I__I	I__I	I__I	I__I	I__I	I__I	I__I	alldeles utmärkt kunde
	1	2	3	4	5	6	7	inte vara bättre

129. Ekonomi

mycket dåligt	I__I	I__I	I__I	I__I	I__I	I__I	I__I	alldeles utmärkt kunde
	1	2	3	4	5	6	7	inte vara bättre

130. Hälsa

	1	2	3	4	5	6	7	
mycket dåligt	I__I	I__I	I__I	I__I	I__I	I__I	I__I	alldeles utmärkt kunde inte vara bättre

131. Fritid

	1	2	3	4	5	6	7	
mycket dåligt	I__I	I__I	I__I	I__I	I__I	I__I	I__I	alldeles utmärkt kunde inte vara bättre

Man kan uppleva en del förändringar inom sig själv under årens lopp. Försök nedan markera (genom att kryssa i den rutan vars siffra bäst anger) hur du känner dig nu.

132. Hörseln

	1	2	3	4	5	6	7	
mycket dålig	I__I	I__I	I__I	I__I	I__I	I__I	I__I	alldeles utmärkt kunde inte vara bättre

133. Synen

	1	2	3	4	5	6	7	
mycket dålig	I__I	I__I	I__I	I__I	I__I	I__I	I__I	alldeles utmärkt kunde inte vara bättre

134. Minnet

	1	2	3	4	5	6	7	
mycket dåligt	I__I	I__I	I__I	I__I	I__I	I__I	I__I	alldeles utmärkt kunde inte vara bättre

135. Konditionen

	1	2	3	4	5	6	7	
mycket dålig	I__I	I__I	I__I	I__I	I__I	I__I	I__I	alldeles utmärkt kunde inte vara bättre

136. Aptiten

	1	2	3	4	5	6	7	
mycket dålig	I__I	I__I	I__I	I__I	I__I	I__I	I__I	alldeles utmärkt kunde inte vara bättre

137. Humöret

	1	2	3	4	5	6	7	
mycket dåligt	I__I	I__I	I__I	I__I	I__I	I__I	I__I	alldeles utmärkt kunde inte vara bättre

138. Energin

	1	2	3	4	5	6	7	
mycket dålig	I__I	I__I	I__I	I__I	I__I	I__I	I__I	alldeles utmärkt kunde inte vara bättre

139. Tålamodet

	1	2	3	4	5	6	7	
mycket dåligt	I__I	I__I	I__I	I__I	I__I	I__I	I__I	alldeles utmärkt kunde inte vara bättre

140. Självförtroendet

mycket dåligt	I__I 1	I__I 2	I__I 3	I__I 4	I__I 5	I__I 6	I__I 7	alldeles utmärkt kunde inte vara bättre

141. Sömnen

mycket dålig	I__I 1	I__I 2	I__I 3	I__I 4	I__I 5	I__I 6	I__I 7	alldeles utmärkt kunde inte vara bättre

142. Känner du dig betydelsefull och uppskattad utanför hemmet?

inte alls	I__I 1	I__I 2	I__I 3	I__I 4	I__I 5	I__I 6	I__I 7	mycket

143. Känner du dig betydelsefull och uppskattad i hemmet?

inte alls	I__I 1	I__I 2	I__I 3	I__I 4	I__I 5	I__I 6	I__I 7	mycket

Har du under den senaste månaden haft....

	Ja	Nej
**144.** Minskad lust att göra saker som du vanligen tycker om	I__I	I__I
**145.** Ofta känt dig illa till mods, deprimerad eller känt att framtiden ser hopplös ut	I__I	I__I
**146.** Nervositet, ångest eller oro	I__I	I__I
**147.** Ängslan över många olika saker	I__I	I__I
**148.** Har du haft en plötslig känsla av ångest eller panik	I__I	I__I

149. Hur många människor känner du och har kontakt med, som har samma intressen som du? Det gäller kontakter både i arbetet och på fritiden.

I__I = ingen

I__I = 1-2 personer

I__I = 3-5 personer

I__I = 6-10 personer

I__I = 11-15 personer

I__I = mer än 15 personer

150. **Hur många människor, som du känner, träffar du eller samtalar du med under en vanlig vecka?** *(Räkna ej med människor som du träffar tillfälligtvis och som du knappast kommer att återse, t ex kunder i en affär)*

I__I = ingen

I__I = 1-2 personer

I__I = 3-5 personer

I__I = 6-10 personer

I__I = 11-15 personer

I__I = mer än 15 personer

151. **Är det ungefär lagom många människor du träffar under ditt dagliga liv?** Skulle du vilja träffa fler eller färre människor?

I__I = färre

I__I = lagom många

I__I = fler

152. **Hur många vänner har du som kan komma hem till dig när som helst och känna sig hemma?** De skulle inte bry sig om, om det var ostädat eller om du höll på att äta. *(Nära släktingar skall inte räknas med!)*

I__I = ingen

I__I = 1-2 personer

I__I = 3-5 personer

I__I = 6-10 personer

I__I = 11-15 personer

I__I = mer än 15 personer

153. **Hur många finns det, i din familj och bland dina vänner, som du kan tala öppet med utan att behöva tänka dig för?**

I__I = ingen

I__I = 1-2 personer

I__I = 3-5 personer

I__I = 6-10 personer

I__I = 11-15 personer

I__I = mer än 15 personer

154. **Finns det någon särskild person som du känner att du verkligen kan få stöd av?**

I__I = ja

I__I = ja, men jag behöver inte det.

I__I = nej

155. **Finns det någon särskild person som känner sig stå väldigt nära dig?**

I__I = ja

I__I = är inte säker

I__I = nej

156. **Har du någon särskild person som du kan dela dina känslor med när du känner dig lycklig?** *Någon som själv skulle känna sig lycklig bara för att du är det?*

I__I = ja

I__I = nej

157. **Har du någon du kan dela dina innersta känslor med och anförtro dig åt?**

I__I = ja

I__I = nej

158. **Händer det att någon håller om dig till tröst eller stöd?**

I__I = ja

I__I = nej

159. **Tror du att de där hemma eller några andra verkligen uppskattar vad du gör för dem?**

I__I = ja

I__I = inte tillräckligt

I__I = nej, inte alls

160. **Finns det människor i din omgivning som du lätt kan be om saker, t.ex. människor som du känner så väl att du kan låna verktyg eller köksgrejor av?**

I__I = nej

I__I = ja

161. **Bortsett från dem där hemma, finns det någon som du kan vända dig till om du är i svårigheter?** *Någon som du lätt kan träffa och som du litar på och kan få verklig hjälp av när du har det besvärligt?*

I__I = nej

I__I = ja

162. **Har du under det senaste året deltagit i någon föreningsaktivitet, frivillig organisation etc. tillsammans med andra?**
(t ex sport, studiecirkel, teatergrupp, sångkör, politisk förening)?

I__I = nej ⟹ gå till fråga **164**

I__I = ja

163. **Hur ofta ägnar du dig åt föreningsaktivitet, klubbverksamhet studiecirkel etc. tillsammans med andra?**

I__I = 1-2 gånger per år

I__I = 1-2 gånger per månad

I__I = 1-2 gånger per vecka

I__I = varje dag

I__I = vet ej

Frågor rörande LIVSINSTÄLLNING

Följande frågor avser att mäta en del av motståndskraften mot sjukdom. Frågorna är således inte inriktade på vad man har för risker att bli sjuk, utan istället vilka tillgångar man har för att hantera påfrestningar.
Varje fråga eller påstående har sju svarsalternativ. Var snäll och sätt kryss i den ruta som närmast uttrycker vad du känner. Lämna endast ett svar på varje fråga. Din omedelbara reaktion på frågan eller påståendet är det vi söker snarare än det du kommer fram till efter lång tids funderande.

164. **Har du känslan av att du inte riktigt bryr dig om vad som händer omkring dig?**

mycket sällan eller aldrig	I__I<br>1	I__I<br>2	I__I<br>3	I__I<br>4	I__I<br>5	I__I<br>6	I__I<br>7	mycket ofta

165. **Har det hänt att du blivit överraskad av beteendet hos personer som du trodde du kände väl?**

har aldrig hänt	I__I<br>1	I__I<br>2	I__I<br>3	I__I<br>4	I__I<br>5	I__I<br>6	I__I<br>7	händer alltid

166. **Har det hänt att du blivit besviken på människor som du litat på?**

har aldrig hänt	I__I<br>1	I__I<br>2	I__I<br>3	I__I<br>4	I__I<br>5	I__I<br>6	I__I<br>7	har hänt ofta

167. **Hittills har ditt liv...**

saknat tydligt mål och mening	I__I<br>1	I__I<br>2	I__I<br>3	I__I<br>4	I__I<br>5	I__I<br>6	I__I<br>7	haft klara mål och mening

168. **Har du känslan av att du behandlas orättvist?**

mycket ofta	I__I<br>1	I__I<br>2	I__I<br>3	I__I<br>4	I__I<br>5	I__I<br>6	I__I<br>7	mycket sällan eller aldrig

169. **Har du känslan av att du är i en ny situation utan att veta vad du skall göra?**

mycket ofta	I__I<br>1	I__I<br>2	I__I<br>3	I__I<br>4	I__I<br>5	I__I<br>6	I__I<br>7	mycket sällan eller aldrig

170. Är dina dagliga sysslor..

en källa till djup glädje eller tillfreds-ställelse	I__I 1	I__I 2	I__I 3	I__I 4	I__I 5	I__I 6	I__I 7	en källa till smärta och leda

171. Har du mycket motstridiga känslor och idéer?

mycket ofta	I__I 1	I__I 2	I__I 3	I__I 4	I__I 5	I__I 6	I__I 7	mycket sällan eller aldrig

172. Händer det att du har känslor inom dig som du helst inte vill känna?

mycket ofta	I__I 1	I__I 2	I__I 3	I__I 4	I__I 5	I__I 6	I__I 7	mycket sällan eller aldrig

173. Många människor - även de med stark självkänsla - kan känna sig helt misslyckade i vissa situationer. Hur ofta har du känt så?

aldrig	I__I 1	I__I 2	I__I 3	I__I 4	I__I 5	I__I 6	I__I 7	mycket ofta

174. När någonting har hänt, har du i allmänhet upptäckt att du ..

över- eller under-värderar betydelsen av vad som hände	I__I 1	I__I 2	I__I 3	I__I 4	I__I 5	I__I 6	I__I 7	såg saker i dess rätta proportioner

175. Hur ofta känner du att det inte finns någon mening med dina dagliga sysslor?

mycket ofta	I__I 1	I__I 2	I__I 3	I__I 4	I__I 5	I__I 6	I__I 7	mycket sällan eller aldrig

176. Hur ofta har du känslor som du är osäker på att kunna behärska?

mycket ofta	I__I 1	I__I 2	I__I 3	I__I 4	I__I 5	I__I 6	I__I 7	mycket sällan eller aldrig

TACK FÖR DIN MEDVERKAN !
