# Supplementary material for: Time trends of comparative self-rated health in adults aged 25-34 in the Northern Sweden MONICA study, 1990-2014
Source: PLoS One. 2017 Nov 20;12(11):e0187896. doi: 10.1371/journal.pone.0187896 (PMC5695772; doi:10.1371/journal.pone.0187896)
Supplement: S4 Text — (PDF) [file pone.0187896.s004.pdf]

monica centre
WHO/OMS
northern sweden

Personnummer:
Namn:
Adress:
MONICA-nummer:

VÄRLDSHÄLSOORGANISATIONENS

KAMPANJ MOT

HJÄRT- och KÄRLSJUKDOMAR

och

DIABETES

2004

FRÅGEFORMULÄR DEL 1

INFORMATION

Det frågeformulär som vi nu ber dig att besvara, innehåller frågor rörande bl.a. hälsa, arbete, fysisk aktivitet, rök-, kost- och motionsvanor.
Alla uppgifter som du lämnar kommer att behandlas konfidentiellt. Resultaten kommer att sammanställas på sådant sätt att det inte går att identifiera enskilda individer.
Om det är något du funderar över angående frågeformulären eller undersökningen, kontakta gärna någon av oss. Du når oss säkrast på telefon på förmiddagarna.
Om vi skulle få problem med att tolka något av dina ifyllda svar, skulle vi vara mycket tacksamma om vi får ringa dig eller skicka e-post för att reda ut det som är oklart. Därför är det bra om du anger det telefonnummer där vi lättast kan nå dig (helst dagtid) och eventuell e-post-adress.

Så här fyller du i enkäten

Att besvara enkäten tar ca 20 minuter. Enkätsvaren registreras maskinellt, därför är det viktigt att de är ifyllda på rätt sätt.

- Använd en bra penna med **svart eller mörkblå färg**. Undvik blyertspenna.

- Sätt ett kryss i de rutor du tycker stämmer bäst för dig. Markera om möjligt inom rutorna.

Så här: ☒

- Om du råkar kryssa fel fyller du i hela den rutan med färg.

Så här: ■

Då kan inte maskinen läsa den. Kryssa sedan i den rätta rutan.

Vi ber dig att noga läsa igenom frågorna och svarsalternativen innan du besvarar alla frågorna.

Mitt telefonnummer dagtid är: [ ]

Jag träffas säkrast:	Efter klockan	Fram till klockan

E-postadress: ______________________

Britt-Inger Eklund
Forskningssjuksköterska
MONICA-projektet
Medicinska kliniken
Norrlands Universitetssjukhus
901 85 UMEÅ
Tel: 090 - 785 25 18
Epost:brittinger.eklund@vll.se

Karin Ruikka
Forskningssjuksköterska
MONICA-projektet
Björkskatans vårdcentral

976 27 LULEÅ
Tel: 0920 - 71 991
Epost: karin.ruikka@nll.se

Endast ett X på varje fråga där inte annat anges

Frågor rörande CIVILSTÅND och BOENDE

1. Vilket är ditt nuvarande civilstånd?

- ☐ ogift
- ☐ gift eller sammanboende
- ☐ skild eller separerad
- ☐ änka/änkling
- ☐ annat____________________

2. Vilka personer sammanbor du med ?

- ☐ vuxen (make, maka, sambo)
- ☐ barn, ange antal [ ]
- ☐ vuxen och barn, ange antal [ ]
- ☐ annan
- ☐ bor helt ensam

3. Var bor du?

- ☐ större tätort - Umeå, Skellefteå, Luleå, Boden, Piteå, Kiruna, orter med än 15 000 invånare
- ☐ annan tätort med mer än 1 000 invånare
- ☐ ort med mindre än 1 000 invånare

4. I vilket land är du född?

- ☐ Sverige ▶ gå till fråga **5**
- ☐ Finland ▶ gå till fråga **6**
- ☐ annat Skandinaviskt land ▶ gå till fråga **6**
- ☐ övriga Europa ▶ gå till fråga **6**
- ☐ annat utomeuropeiskt land ▶ gå till fråga **6**

5. Var är du född? *(Ange vilken kyrkoförsamling eller ort du tillhörde vid födseln)?*

Församling	Ort	län

6. **Vilken är den högsta utbildningsnivå som du har avslutat?**

- ☐ folkskola
- ☐ grundskola
- ☐ folkhögskola motsvarande grundskolekompetens
- ☐ yrkesskola
- ☐ realskola
- ☐ flickskola
- ☐ fackskola
- ☐ gymnasieskola
- ☐ folkhögskola motsvarande gymnasiekompetens
- ☐ högskola eller universitet

7. **Hur många år har du gått i skola eller ägnat dig åt studier på heltid?** ☐ **år**
(Ange antal år från första klass och framåt)

Frågor rörande TOBAKSVANOR

8. **Röker du cigaretter för närvarande?**

- ☐ ja, regelbundet (1 cigarett eller mer per dag) ► gå till fråga **9**
- ☐ nej ► gå till fråga **12**
- ☐ ibland (mindre än 1 cigarett per dag) ► gå till fråga **10**

9. **Ungefär hur många cigaretter röker du i genomsnitt per dag?**

(Ange antal cigaretter/dag) ☐ ► gå till fråga **16**

10. **Hur många dagar i veckan röker du cigaretter?**

- ☐ vanligtvis en dag eller mindre
- ☐ vanligtvis 2 till 4 dagar
- ☐ nästan varje dag

11. **Ungefär hur många cigaretter röker du i genomsnitt per vecka?**

(Ange antal cigaretter/vecka) ☐

12. **Har du någonsin rökt cigaretter regelbundet tidigare?**

- ☐ ja, regelbundet (1 cigarett eller mer per dag) ► gå till fråga **13**
- ☐ nej ► gå till fråga **18**

13. När slutade du att röka cigaretter regelbundet?

(Ange årtal) ☐

14. Om du slutade röka under det senaste året, när slutade du?

☐ för mindre än en månad sedan
☐ för 1-6 månader sedan
☐ för 7-12 månader sedan

15 Vilken är den främsta orsaken till att du slutat röka? *(Ange endast **ett** alternativ)*

☐ av hälsoskäl, på eget initiativ
☐ på inrådan av läkare/sjukvårdspersonal
☐ pga. annan information/upplysning
☐ pga. tryck från kamrater/familjemedlemmar
☐ av andra skäl

16. Vilket är det högsta antal cigaretter/dag, som du rökt under en så lång period som ett år?

(Ange *antal cigaretter/dag)* ☐

17. Hur gammal var du när du började röka cigaretter?

(Ange ålder i år) ☐

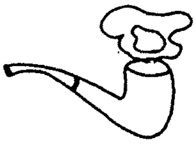

18. Hur du någonsin rökt <u>pipa?</u>

☐ ja, för närvarande regelbundet ► gå till fråga **19**
☐ nej ► gå till fråga **20**
☐ jag röker pipa ibland (mindre än 1 gång/dag) ► gå till fråga **19**
☐ tidigare, men inte nu ► gå till fråga **20**

19. Hur många gram tobak röker du per vecka?

Ett paket piptobak väger som regel 50 gram *(Ange antal gram/vecka)* ☐

20. Hur du någonsin rökt <u>cigarrer eller cigariller</u>

☐ ja, för närvarande regelbundet ► gå till fråga **21**
☐ nej ► gå till fråga **22**
☐ jag röker ibland (mindre än 1 /dag) ► gå till fråga **21**
☐ tidigare, men inte nu ► gå till fråga **22**

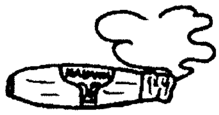

21. **Ungefär hur många cigarrer/cigariller röker du i genomsnitt per vecka?**

(Ange antal cigarrer/cigariller/vecka) ☐

OBS! Fråga 22 skall endast fyllas i av icke-rökare och de som röker ibland

22. **Hur många timmar i medeltal per dag, är du i närheten av andra människors tobaksrök?**

(Ange antal timmar/dag du inandas eller känner lukten av tobaksrök) ☐

23. **Har du någonsin använt snus?**

- ☐ ja, snusade tidigare men inte nu
- ☐ ja, snusar mindre än 2 dosor per vecka
- ☐ ja, snusar 2 - 4 dosor per vecka
- ☐ ja, snusar mer än 4 men mindre än 7 dosor per vecka
- ☐ ja, snusar 7 dosor eller fler per vecka
- ☐ nej ► gå till fråga **26**

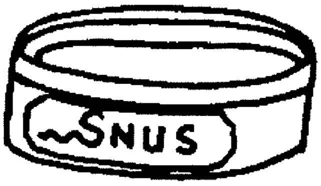

24. **Hur många år har du använt snus?**

(Ange antal år) ☐

25. **Började du att snusa i samband med att du slutade röka?**

- ☐ ja
- ☐ nej
- ☐ jag både röker och snusar

26. **Använder du dagligen andra nikotinprodukter?**

- ☐ ja, tuggtobak
- ☐ ja, nikotinersättningsmedel (nikotintuggummi, nikotinplåster, nikotinnässpray, nikotinsugtabletter)
- ☐ annat______________________________
- ☐ nej

Frågor rörande HÄLSA

27. **Har du vid något tillfälle av läkare eller annan sjukvårds personal fått besked om att du har högt blodtryck?**

- ☐ Ja
- ☐ nej ► gå till fråga **29**

28. **Har du under de senaste 2 veckorna tagit läkemedel mot förhöjt blodtryck?**

☐ ja

☐ nej

☐ osäker på om den medicin jag äter är mot högt blodtryck

29. **Har du fått ditt blodtryck mätt under det senaste året?**

☐ ja

☐ nej

30. **Har eller hade någon av dina föräldrar behandling för högt blodtryck?**

☐ ja, min pappa

☐ ja, min mamma

☐ ja, båda föräldrarna

☐ nej, ingen av föräldrarna

☐ vet ej

31. **Har du vid något tillfälle av läkare eller annan sjukvårdspersonal fått besked om att du har förhöjda kolesterol-/blodfettvärden?**

☐ ja

☐ nej

32. **Har läkare eller annan sjukvårdspersonal föreskrivet en särskild diet till dig för att sänka dina kolesterol-/blodfettnivåer?**

☐ ja

☐ nej

☐ håller en föreskriven diet, men vet ej om den är till för att sänka mina kolesterol-/blodfettnivåer

33. **Har du under de senaste två veckorna tagit medicin, föreskriven av läkare för att sänka kolesterol-/blodfettvärdet?**

☐ ja

☐ nej

☐ vet ej om medicinen jag äter är för att sänka kolesterolet

34. **Har du fått ditt kolesterol/blodfett mätt under det senaste året?**

☐ ja

☐ nej

35. **Har du under de senaste två veckorna tagit acetylsalicylika t ex Albyl, Aspirin, Bamyl, Bamycor, Trombyl, Magnecyl, Asasantin Retard Seloken ZOC/ASA för att förebygga eller behandla hjärtsjukdom?**

☐ ja

☐ nej

☐ nej, men jag använder acetylsalicylika regelbundet för att behandla annan sjukdom än hjärtsjukdom

36. **Har du under de senaste två veckorna tagit acetylsalicylika t ex Albyl, Aspirin, Bamyl, Bamycor, Trombyl, Magnecyl, Asasantin Retard, Seloken ZOC/ASA för att förebygga eller behandla slaganfall (propp i hjärnan)?**

☐ ja

☐ nej

☐ nej, men jag använder acetylsalicylika regelbundet för att behandla annan sjukdom än slaganfall

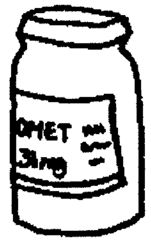

37. **Har du under de senaste 14 dagarna använt något läkemedel som ordinerats av läkare?**

☐ ja

☐ nej

38. **Om du svarat "ja" i så fall vilken eller vilka mediciner?**

1 ____________________ 4 ____________________

2 ____________________ 5 ____________________

3 ____________________ 6 ____________________

39. **Har du under de senaste 14 dagarna använt någon annan medicin?**
(T.ex. naturmedicin, vitamin- eller järnpreparat, förebyggande medicin)

☐ ja

☐ nej

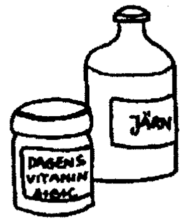

40. **Om du svarat "ja" i så fall vilken eller vilka mediciner?**

1 ____________________ 4 ____________________

2 ____________________ 5 ____________________

3 ____________________ 6 ____________________

41. **Får du smärtor - stickningar - ont i bröstet när du går uppför backar eller trappor, eller när du går fort på plan mark?**

- ☐ ja
- ☐ nej

42. **Får du smärtor - stickningar - ont i bröstet när du går i vanlig takt på plan mark?**

- ☐ ja
- ☐ nej

Om** du svarat **nej** på både fråga 41 och 42 gå till fråga **47

43. **Om du får smärtor eller obehag i bröstet i samband med att du rör dig, brukar du då?**

- ☐ stanna
- ☐ sakta ner farten
- ☐ fortsätta i samma takt

44. **Om du stannar eller saktar ner, försvinner smärtorna då?**

- ☐ ja
- ☐ nej

45. **Om de försvinner, hur snart försvinner de?**

- ☐ efter mindre än 10 minuter
- ☐ efter mer än 10 minuter

46. **VAR BRUKAR DU KÄNNA DESSA SMÄRTOR ELLER OBEHAG?**

(Markera med X på figuren där du känner smärta eller obehag. Om smärta eller obehag känns ut i armarna eller upp mot halsen, markera med X även där!)

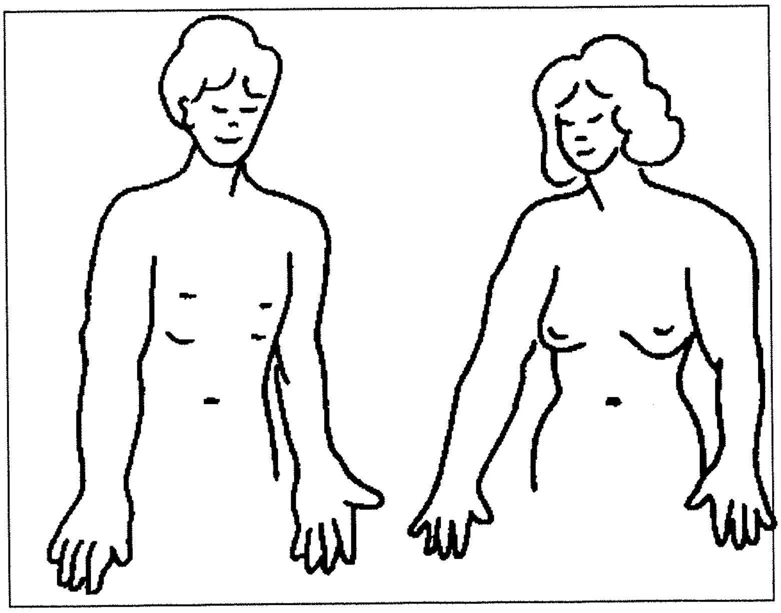

47. **Har du någon gång haft svår smärta i bröstet som varat i en halvtimme eller mer?**

☐ ja

☐ nej

48. **Har du legat på sjukhus för säker hjärtinfarkt** (propp i hjärtat)?

☐ ja ange årtal [ ] och sjukhus ______________________

☐ nej

☐ vet ej om det var en säker hjärtinfarkt

49. **Brukar du få smärtor i vaderna, när du går i uppförsbackar, trappor eller på plan mark?**

☐ ja

☐ nej ▶ Gå till fråga **51**

50. **Om du får smärtor i vaderna, när du går i uppförsbackar, trappor eller på plan mark försvinner smärtorna om du stannar?**

☐ ja

☐ nej

51. **Blir du andfådd av att gå två trappor upp eller motsvarande i samma takt som jämnåriga?**

☐ ja

☐ nej

52. **Har du haft slaganfall (hjärnblödning eller propp i hjärnan)?**

☐ ja ange årtal [ ] och sjukhus ______________________

☐ nej

53. **Har du diabetes/sockersjuka?**

☐ ja

☐ nej ▶ gå till fråga **57**

54. **Hur behandlar du din diabetes/sockersjuka?**

☐ insulin

☐ tabletter

☐ både insulin och tabletter

☐ enbart kost

55. **Hur gammal var du när du fick diabetes/sockersjuka?**

[ ] år

56. Om du behandlas med insulin, vilket år började du behandlas med insulin ?

☐

57. Finns det någon i din släkt som har/har haft diabetes/sockersjuka (*Flera alternativ är möjliga).* Med släkt menas i detta fall: Föräldrar, syskon och barn

- ☐ ja, min mamma
- ☐ ja, min pappa
- ☐ ja, båda föräldrarna
- ☐ ja, något/några syskon
- ☐ ja, något/några barn
- ☐ nej
- ☐ vet ej

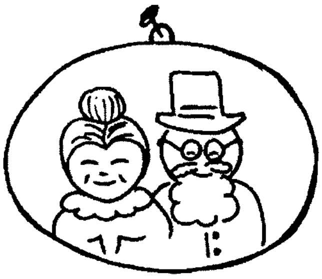

58. Har du mångåriga magbesvär?

- ☐ ja
- ☐ nej

Flera alternativ är möjliga!

59. Finns det någon i din släkt som <u>avlidit</u> i hjärtinfarkt <u>före</u> 65 års ålder?
Med släkt menas i detta fall: Föräldrar, syskon, fastrar, farbröder, mostrar och morbröder.

☐ ja, min mamma	Hon var då		år
☐ ja, min pappa	Han var då		år
☐ ja, något/några syskon			år
☐ ja, någon/några av fastrar, farbröder, mostrar och morbröder.			år
☐ nej.			år
☐ vet ej			år

60. Finns det någon i din släkt som <u>avlidit</u> i slaganfall (propp eller blödning i hjärnan) <u>före</u> 65 års ålder? Med släkt menas i detta fall: Föräldrar, syskon, fastrar, farbröder, mostrar och morbröder

☐ ja, min mamma	Hon var då		år
☐ ja, min pappa	Han var då		år
☐ ja, något/några syskon			år
☐ ja, någon/några av fastrar, farbröder, mostrar och morbröder.			år
☐ nej.			år
☐ vet ej			år

61. **Finns det någon i din släkt som har/har haft kroppspulsåderbråck i buken?**
Med släkt menas i detta fall föräldrar, syskon, fastrar, farbröder, mostrar och morbröder.

- ☐ ja, min mamma
- ☐ ja, min pappa
- ☐ ja, något/några syskon
- ☐ ja, någon/några av fastrar, farbröder, mostrar och morbröder
- ☐ nej
- ☐ vet ej

Endast ett X på varje fråga där inte annat anges

62. **Har du vid något tillfälle av läkare eller annan sjukvårdsperson fått besked om att du har lungsjukdomen KOL?**

- ☐ ja
- ☐ nej

63. **Vaknar du nattetid pga. andfåddhet?**

- ☐ ja, varje natt
- ☐ ja, en gång per vecka eller mer
- ☐ ja, mindre än en gång per vecka (1-3 ggr/mån)
- ☐ nej

64. **Har du genomgått ballongvidgning av hjärtats blodkärl eller kranskärlsoperation?**

- ☐ ja
- ☐ nej
- ☐ vet ej

65. **Är du opererad för klaffel i hjärtat?**

- ☐ ja
- ☐ nej
- ☐ vet ej

66. **Kontrolleras eller behandlas du av läkare för reumatiska led- eller muskelbesvär i form av smärtor/värk, stelhet?** *Flera alternativ är möjliga*

- ☐ ja muskelbesvär
- ☐ ja, ledbesvär
- ☐ nej

67. **Har du varit med om någon olycka som föranlett läkarbesök på grund av nackskada eller skallskada?** *Flera alternativ är möjliga*

- ☐ ja, whiplash- (pisksnärts-) skada
- ☐ ja, annan nackskada
- ☐ ja, skallskada
- ☐ nej ▶ gå till fråga **70**

68. **Har du varit besvärsfri efter nackskadan under en period på mer än 3 månader efter olyckan?**

- ☐ ja, och jag är fortfarande besvärsfri
- ☐ ja, men jag har sedan haft perioder med nacksmärtor
- ☐ ja, men jag har sedan haft ihållande dagliga nacksmärtor
- ☐ nej, jag har aldrig varit besvärsfri efter nackskadan

69. **Hade du innan nackskadan en eller flera perioder med nacksmärtor?**

- ☐ ja, ständiga besvär
- ☐ ja, några gånger i veckan
- ☐ ja, några gånger i månaden
- ☐ ja, enstaka gånger
- ☐ nej, aldrig

70 **Har du under den senaste veckan fått nacksmärtor för första gången i ditt liv?** *Besvaras endast om du svarat nej på fråga **67***

- ☐ ja
- ☐ nej

71. **Om du tidigare besvärats av periodvisa nacksmärtor har du under senaste veckan haft en sådan period av nacksmärtor?** *Besvaras endast om Du svarat nej på fråga **67***

- ☐ ja
- ☐ nej

72. **Har du någon gång under ditt liv behandlats för fraktur (benbrott) på någon del av kroppen?**

- ☐ ja
- ☐ nej ▶ gå till fråga **75**

73. **Har du under de senaste 5 åren behandlats för fraktur (benbrott) som orsakats av lindrigt våld t ex fall på grund av ishalka, snubbling på matta, fall från stol?**

- ☐ ja
- ☐ nej

74. **Markera vilken eller vilka frakturer (benbrott) du har behandlats för under de senaste 5 åren!** *Flera alternativ är möjliga.*

- ☐ handledsfraktur
- ☐ höftfraktur (lårbenshalsbrott)
- ☐ överarmsfraktur eller axelfraktur
- ☐ fraktur på ryggkota
- ☐ knäfraktur
- ☐ fotledsfaktur
- ☐ fraktur på annat ställe

75. **Hur ofta borstar du dina tänder?**

- ☐ mindre än en gång per dag
- ☐ en gång per dag
- ☐ två gånger per dag eller oftare
- ☐ jag har inga egna tänder utan använder helprotes

76. **Brukar ditt tandkött blöda vid tandborstning?**

- ☐ ja, alltid
- ☐ ja, ibland
- ☐ nej, aldrig

77. **Använder du tandtråd, mellanrumsborste eller trekantig tandsticka?**

- ☐ nej, jag använder inte tandtråd, mellanrumsborste eller trekantig tandsticka
- ☐ ja, minst en gång per månad
- ☐ ja, minst en gång per vecka
- ☐ ja, minst en gång per dagen

78. **Snarkar du när du sover?**

- ☐ ja, alltid
- ☐ ja, nästan alltid
- ☐ ja, ibland
- ☐ nej, nästan aldrig
- ☐ nej, aldrig
- ☐ vet inte om jag snarkar

79. **Besväras du av trötthet under dagen?**

- ☐ ja, alltid
- ☐ ja, nästan alltid
- ☐ ja, ibland
- ☐ nej, nästan aldrig
- ☐ nej, aldrig

80. **Har din make/maka/sambo märkt att du har andningsuppehåll under sömn?**

- ☐ ja, i stort sett varje natt
- ☐ ja, ofta
- ☐ ja, ibland
- ☐ nej, nästan aldrig
- ☐ nej, aldrig
- ☐ vet ej

81. **Hur många mil kör du bil per år?**

Ange antal mil per år ☐

- ☐ jag har inget körkort
- ☐ jag kör aldrig bil

82. **Hur är det kaffe du dricker vanligtvis tillagat?**

- ☐ jag dricker mest kokkaffe
- ☐ jag dricker mest bryggkaffe
- ☐ annat (t ex snabbkaffe)
- ☐ jag dricker mest kaffe tillagat i s.k. perkulator
- ☐ jag dricker inte kaffe ▶ gå till fråga **84**

83. **Hur många koppar kaffe dricker du i genomsnitt varje dag?**
(Ange antal kaffekoppar) ☐

84. **Vilket sorts dricksvatten använder du huvudsakligen?**

- ☐ kommunalt vatten
- ☐ egen brunn
- ☐ lokal vattenförening eller liknande

85. **Hur ofta äter du lokalt fiskad fisk (från insjöar, älvar, kustvattenområden)?**

- ☐ aldrig
- ☐ någon gång per år
- ☐ 1 gång varannan månad
- ☐ 1 gång per månad
- ☐ 2 ggr per månad
- ☐ 1 gång per vecka
- ☐ 2 - 3 ggr per vecka
- ☐ 1 gång per dag eller mer

Frågor om ARBETE

Med arbete avses din huvudsakliga sysselsättning.
OBS: Ange endast ett alternativ!

86. **Hur är dina nuvarande arbetsförhållanden?**

- ☐ fast anställning, anställning tills vidare
- ☐ egen företagare
- ☐ tillfällig anställning, vikariat, beredskapsarbete
- ☐ arbetar i hemmet
- ☐ arbetslös ► gå till fråga **109**
- ☐ studerande ► gå till fråga **107**
- ☐ pensionerad ► gå till fråga **108**

87. **Hur många timmar förvärvsarbetar du i normala fall per vecka?**

- ☐ jag förvärvsarbetar inte
- ☐ mindre än 15 timmar
- ☐ 15-35 timmar
- ☐ mer än 35 timmar

88. **Vad har du för arbetstider i normala fall?**

- ☐ jag förvärvsarbetar inte
- ☐ fast arbetstid (t ex 7-16 eller 8-17)
- ☐ skiftarbete (t ex 2-skift, 3-skift, 5-skift)
- ☐ varierande arbetstider (ibland dagtid, ibland kvällstid, ibland helger)

89. **Är ditt arbete fysiskt (kroppsligt) tungt?**

- ☐ ja, ofta
- ☐ ja, ibland
- ☐ nej, sällan
- ☐ nej, så gott som aldrig

90. **Kräver ditt arbete att du arbetar mycket fort?**

- ☐ ja, ofta
- ☐ ja, ibland
- ☐ nej, sällan
- ☐ nej, så gott som aldrig

91. **Är ditt arbete psykiskt påfrestande?**

- ☐ ja, ofta
- ☐ ja, ibland
- ☐ nej, sällan
- ☐ nej, så gott som aldrig

92. **Har du tillräckligt med tid för att hinna med arbetsuppgifterna?**

- ☐ ja, ofta
- ☐ ja, ibland
- ☐ nej, sällan
- ☐ nej, så gott som aldrig

93. **Förekommer det ofta motstridiga krav i ditt arbete?**

- ☐ ja, ofta
- ☐ ja, ibland
- ☐ nej, sällan
- ☐ nej, så gott som aldrig

94. **Får du lära dig nya saker i ditt arbete?**

- ☐ ja, ofta
- ☐ ja, ibland
- ☐ nej, sällan
- ☐ nej, så gott som aldrig

95. **Kräver ditt arbete skicklighet?**

- ☐ ja, ofta
- ☐ ja, ibland
- ☐ nej, sällan
- ☐ nej, så gott som aldrig

96. **Kräver ditt arbete påhittighet?**

- ☐ ja, ofta
- ☐ ja, ibland
- ☐ nej, sällan
- ☐ nej, så gott som aldrig

97. **Innebär ditt arbete att man gör samma sak om och om igen?**

- ☐ ja, ofta
- ☐ ja, ibland
- ☐ nej, sällan
- ☐ nej, så gott som aldrig

98. **Har du frihet att bestämma hur ditt arbete skall utföras?**

- ☐ ja, ofta
- ☐ ja, ibland
- ☐ nej, sällan
- ☐ nej, så gott som aldrig

99. **Har du frihet att bestämma vad som skall utföras i ditt arbete?**

- ☐ ja, ofta
- ☐ ja, ibland
- ☐ nej, sällan
- ☐ nej, så gott som aldrig

100. **Har du i vanliga fall möjlighet att tala med dina arbetskamrater under raster, om du skulle vilja det?**
- ☐ ja, alltid
- ☐ ja, för det mesta
- ☐ nej, jag har inga raster
- ☐ nej, jag har inga raster tillsammans med arbetskamrater

101. **Är ditt arbete av den karaktären att du kan lämna det ett tag om du vill tala med en arbetskamrat?**
- ☐ ja, för det mesta
- ☐ ja, ibland
- ☐ bara för brådskande ärenden
- ☐ nej, det är helt omöjlig

102. **Har du, som en del i ditt arbete, ofta kontakt med dina arbetskamrater?**
- ☐ ja, ständigt kontakt med arbetskamrater i arbetet
- ☐ ja, ibland kontakt med arbetskamrater i arbetet
- ☐ nej, jag arbetar för det mesta ensam
- ☐ nej, jag arbetar ständigt ensam

103. **Hur ofta är du vanligen tillsammans med en eller flera av dina arbetskamrater på fritiden?**
- ☐ en eller flera ggr/vecka
- ☐ en eller ett par ggr/månad
- ☐ en eller ett par ggr/år
- ☐ sällan eller aldrig

104. **När besökte någon av dina arbetskamrater dig senast?**
- ☐ för en till fyra veckor sedan
- ☐ för en till tolv månader sedan
- ☐ för mer än ett år sedan
- ☐ har aldrig haft besök av någon arbetskamrat

105. **Tror du att det finns risk att du blir arbetslös?**
- ☐ ja, inom den närmaste tiden
- ☐ nej

106. **Antag att du har ett arbete och blir uppsagd. Hur stora chanser tror du att du har att få ett nytt arbete inom en månad?**
- ☐ mycket stora
- ☐ ganska stora
- ☐ små
- ☐ inga chanser

107. **På vilken nivå studerar du?** *(Denna fråga besvaras endast av studerande)*

- ☐ grundskolenivå
- ☐ gymnasial nivå
- ☐ eftergymnasial nivå
- ☐ annan, vilken ____________________

108. **Har du varit arbetslös någon gång?**

- ☐ ja, för mer än 12 månader sedan
- ☐ ja, någon gång under de senaste 12 månaderna
- ☐ nej, aldrig ► gå till fråga **110**

109. **Om du varit/eller är arbetslös, hur länge har du sammanlagt varit det?**

- ☐ mindre än 3 månader
- ☐ 3-12 månader
- ☐ mer än 12 månader

110. **Har du tvingats flytta någon gång?**

- ☐ ja, för mer än 12 månader sedan
- ☐ ja, de senaste 12 månaderna
- ☐ nej aldrig ► gå till fråga **112**

111. **Vilken var anledningen till flyttningen?**

- ☐ familjeskäl
- ☐ arbetsmarknadsskäl
- ☐ annat

112. **Har någon eller några personer som betytt eller betyder mycket för dig, flyttat så att du inte längre kan umgås med honom/henne eller dem?**

- ☐ ja
- ☐ nej ► gå till fråga **114**

113. **Om du svarat "ja" på föregående fråga, vilken var anledningen till flyttningen?**

- ☐ familjeskäl
- ☐ arbetsmarknadsskäl
- ☐ annat

114. Är du just nu sjukskriven, har ålderspension, har förtidspension, har sjukpension eller sjukbidrag?

☐ ja, ålderspension/sjukpension på heltid

☐ ja, ålderspension/sjukpension på deltid

☐ ja, förtidspension/sjukbidrag

☐ ja, sjukskriven

☐ nej

115. Vilket är ditt yrke/din befattning? Om du inte förvärvsarbetar nu ska du ange yrke/sysselsättning som du tidigare huvudsakligen haft
(Undvik allmänna yrkesbeteckningar som lärare, tjänsteman, byggnadsarbetare etc. Skriv i stället högstadielärare, kontorist, byggnadssnickare etc. så att det framgår mer exakt vad du arbetar/arbetade med).

____

____

116. Beskriv kortfattat dina arbetsuppgifter, samt ange arbetsplats eller typ av företag.

____

____

Besvaras av gifta/samboende

117. Vilket är din make/makas/samboendes yrke eller befattning?
Om han/hon inte förvärvsarbetar nu ska du ange den yrke/sysselsättning som han/hon tidigare huvudsakligen haft)

____

118. Beskriv kortfattat hans/hennes arbetsuppgifter

____

Besvaras av de som är egna företagare

119. Hur många anställda har du i ditt företag?

Antal anställda [ ]

120. Är ditt jordbruk/lantbruk att betrakta som ett litet eller stort jordbruk.
Besvaras endast av jordbrukare *(Kryssa för det alternativ som stämmer bäst med din situation, samt ange hur mycket skog och åkermark du har)*

☐ litet jordbruk. Hektar skog [ ] och hektar åkermark [ ]

☐ stort jordbruk. Hektar skog [ ] och hektar åkermark [ ]

Frågor om FYSISK AKTIVITET

Sätt bara X i ett av alternativen

121. Hur mycket har du rört dig eller ansträngt dig kroppsligt på din <u>fritid</u> under det senaste året?

☐ **knappast något alls**

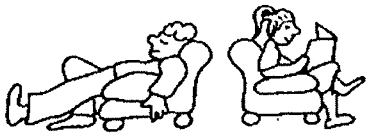

☐ **mestadels stillasittande, ibland någon promenad eller liknande.**

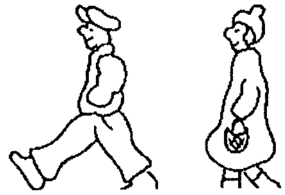

☐ **lättare fysisk ansträngning <u>minst 2 timmar i veckan</u>,**
t.ex. gång och cykling (även till och från arbetet eller skolan) fiske, dans etc.

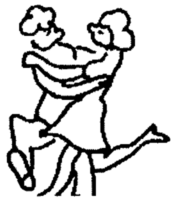

☐ **mer ansträngande motion <u>1-2 timmar i veckan</u>,**
t.ex. motionslöpning, tennis, simning, badminton, motionsgymnastik.

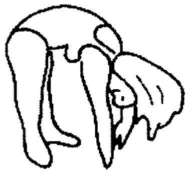

☐ **mer ansträngande motion <u>minst 3 timmar i veckan</u>,**
t.ex. motionslöpning, tennis, simning, badminton, motionsgymnastik

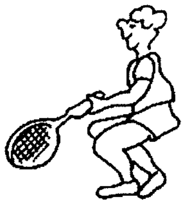

☐ **<u>hård träning eller tävling</u> regelbundet och <u>flera gånger i veckan</u>** där den fysiska ansträngningen är stor
t.ex. löpning, skidåkning, fotboll, simning

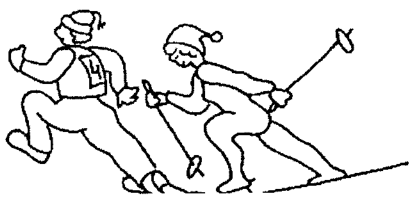

Sätt bara X i ett av alternativen

122. **Hur mycket har du rört dig eller ansträngt dig kroppsligt i ditt <u>arbete</u> under det senaste året?**
Denna fråga besvaras också om du arbetar i hemmet.

☐ **jag har ålderspension/förtidspension**

☐ **stillasittande arbete**
Du har övervägande stillasittande arbete och går inte mycket ut under tiden .T.ex. skrivbordsarbete, urmakeri och montering av lättare delar.

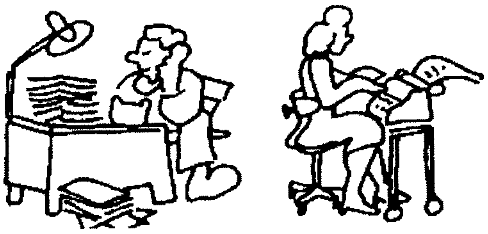

☐ **lätt, men något rörligt arbete.**
Du har ett arbete där du går ganska mycket men inte bär eller lyfter tyngre saker. T.ex. rörligt expeditionsarbete, lätt industriarbete, förmanssysslor, sådan undervisning där man går mycket, affärsbiträde, hushållsarbete

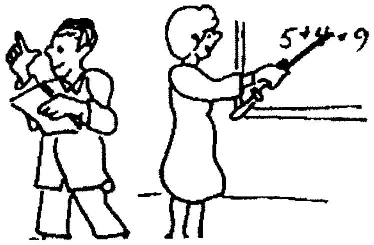

☐ **måttligt tungt arbete.**
Du går mycket och lyfter dessutom ganska mycket eller går uppför trappor eller backar. T.ex. brevbärare, arbete vid tyngre industri, byggnadssnickeri, rörläggningsarbete, vårdarbete

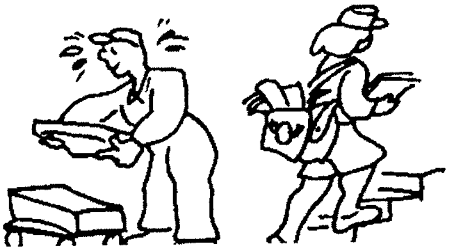

☐ **tungt arbete.**
Du har ett tungt kroppsarbete, lyfter tunga föremål och anstränger dig mycket kroppsligt. T.ex. skogsarbete, tungt lantbruksarbete, fiske med tunga redskap, byggnadsarbete

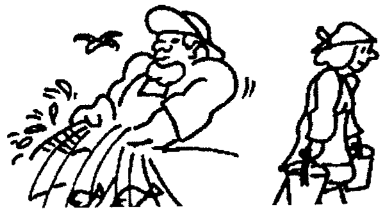

Ifylles endast av kvinnor
Männen kan sluta att fylla i formuläret här.

123. **Hur många gånger har du varit gravid**

[ ] gånger

124. **Hur många barn har du fött?**

[ ] barn

125. **Om du fött barn, hade du då graviditetsdiabetes (dvs. socker i urinen eller förhöjt blodsocker) under graviditeten?**

- ☐ ja
- ☐ nej
- ☐ har inga barn
- ☐ vet ej

Om **ja** ange födelsevikt på barnet/barnen

[ ] g	[ ] g	[ ] g
[ ] g	[ ] g	[ ] g

126. **Har du fortfarande menstruation varje månad?**

- ☐ ja, regelbundet varje månad ► gå till fråga **127**
- ☐ ibland, men inte så regelbundet som tidigare, eller att menstruationen har upphört de senaste sex månaderna ► gå till fråga **127**
- ☐ nej, det är mer än sex månader sedan den upphörde ► gå till fråga **132**
- ☐ jag är gravid ► gå till fråga **127**

127. **Har du tagit preventivmedel (p-piller) eller preventivinjektion de senaste två månaderna**

- ☐ ja
- ☐ nej

128. **Om du använder p-piller just nu, hur var dina menstruationer året innan du började med p-piller?**

- ☐ regelbundna
- ☐ oregelbundna
- ☐ hade ingen menstruation

129. **Vilket/vilka preventivmedel har du använt under senaste året?**

- ☐ använder inte preventivmedel
- ☐ p-piller
- ☐ minipiller
- ☐ hormonspiral
- ☐ p-stavar
- ☐ p-spruta
- ☐ annan metod

130. **Har du någon gång under de senaste 2 åren vid något tillfälle haft mer än 35 dagar mellan menstruationerna?**

- ☐ ja
- ☐ nej

131. **Har du ökad behåring på kroppen i jämförelse med jämnåriga kvinnor?**

- ☐ ja
- ☐ nej

Om du menstruerar eller är gravid så kan du sluta fylla i enkäten här. Övriga kvinnor går till nästa fråga.

Ifylles endast av kvinnor som är i eller har passerat klimakteriet.

132. **Hur gammal var du när menstruationerna upphörde helt?**

☐ år

133 **Har du använt något östrogenpreparat (ex tabletter eller plåster) för dina klimakteriebesvär under den senaste månaden?**

- ☐ ja
- ☐ nej

TACK FÖR DIN MEDVERKAN !

Var vänlig tag med detta formulär när du går till undersökningen

monica centre
WHO/OMS
northern sweden

Personnummer:

Namn:

Adress:

MONICA-nummer:

VÄRLDSHÄLSOORGANISATIONENS KAMPANJ MOT HJÄRT- och KÄRLSJUKDOMAR och DIABETES

2004

FRÅGEFORMULÄR DEL 2

____ *Frågor rörande LIVSKVALITET OCH SOCIALT STÖD* ____

Besvara följande frågor genom att ange det svarsalternativ som bäst stämmer överens med din personliga situation/uppfattning

134. Hur tycker du ditt allmänna hälsotillstånd varit det senaste året?

- ☐ mycket gott
- ☐ ganska gott
- ☐ någorlunda
- ☐ tämligen dåligt
- ☐ dåligt

135. Hur bedömer du ditt allmänna hälsotillstånd jämfört med andra personer i din egen ålder?

- ☐ det är bättre
- ☐ det är sämre
- ☐ ungefär likadant

136. Jämfört med andra personer av samma ålder och kön som du själv, känner du dig "yngre" eller "äldre" än dessa rent kroppsligen?

- ☐ ja, jag känner mig kroppsligen "yngre"
- ☐ jag känner mig kroppsligen i samma ålder som jämnåriga
- ☐ ja, jag känner mig kroppsligen "äldre"
- ☐ vet inte

137. Brukar andra personer uppfatta att du förefaller "yngre" eller "äldre" än jämnåriga av samma kön?

- ☐ ja, jag uppfattas "yngre"
- ☐ jag uppfattas som jämnårig
- ☐ ja, jag uppfattas "äldre"
- ☐ vet inte

Följande frågor gäller din fysiska aktivitet, dvs. hur mycket du rör på dig. Frågorna är till för alla, oavsett om du rör dig mycket eller litet. Tänk på all form av fysisk aktivitet som du utför. Det kan vara på ditt arbete, under din fritid, i hemmet och utomhus. Tänk även på hur du förflyttar dig från en plats till en annan.

Med fysisk aktivitet menas all typ av rörelse som ger ökad energiomsättning. Fysisk aktivitet omfattar all typ av muskelaktivitet t.ex. städning, snöskottning, fysisk belastning i arbetet hobbyverksamheter som golf, dans, motion och träning.

138. Hur många gånger har du de senaste 7 dagarna utfört mycket ansträngande fysisk aktivitet som du upplevde som mycket arbetsam och fick dig att svettas och/eller andas mycket kraftigare än normalt?
Exempel på sådana aktiviteter är löpning, skidåkning, fotboll, innebandy och andra bollsporter, aerobics eller ansträngande motionsgymnastik och cykling i högre tempo. Svara endast för de aktiviteter som pågick minst 10 minuter i sträck

☐ ingen gång ▶ gå till fråga **140**

[ ] gånger under den senaste 7 dagarna

139. Hur mycket tid använde du i genomsnitt per tillfälle på mycket ansträngande fysisk aktivitet?

[ ] timmar [ ] minuter

☐ vet inte/osäker

140. Hur många gånger har du de senaste 7 dagarna utfört måttligt ansträngande fysisk aktivitet som du upplevde som måttligt arbetsam och fick dig att bli varm och/eller andas kraftigare än normalt?
Exempel på sådana aktiviteter är gräsklippning och annat trädgårdsarbete, tyngre hushållsarbete, promenad i rask takt, cykling, simning eller andra motionsaktiviteter i måttligt tempo. Svara endast för de aktiviteter som pågick minst 10 minuter i sträck

☐ ingen gång ▶ gå till fråga **142**

[ ] gånger under den senaste 7 dagarna

141. Hur mycket tid använde du i genomsnitt per tillfälle på måttligt ansträngande fysisk aktivitet?

[-] timmar [ ] minuter

☐ vet inte/osäker

142. Hur många gånger har du de senaste 7 dagarna *gått eller promenerat* i en takt som inte fick dig att svettas eller andas kraftigare än normalt?
Detta gäller då du gått/promenerat som en del av arbete, i hemmet, för att göra ärenden och på din fritid. Svara endast när du gick eller promenerade minst 10 min.i sträck

☐ ingen gång ▶ gå till fråga **144**

[ ] gånger under den senaste 7 dagarna

143. **Hur mycket tid använde du i genomsnitt per tillfälle på att gå eller promenera?**

a) På vardagar

☐ timmar ☐ minuter

☐ vet inte/osäker

b) På helgen

☐ timmar ☐ minuter

☐ vet inte/osäker

144. **Hur lång tid har du per dag under de senaste 7 dagarna tillbringat sittande i samband med arbete, studier, i hemmet och på din fritid?**
Försök uppskatta hur många timmar i genomsnitt. Exempel på detta är tid vid skrivbordet, hemma hos vänner, att sitta och äta eller prata, och att sitta och se på film eller TV

a) På vardagar

☐ timmar ☐ minuter

☐ vet inte/osäker

b) På helgen

☐ timmar ☐ minuter

☐ vet inte/osäker

De följande frågorna handlar om dina personliga förhållanden och om människorna i din omgivning. Frågorna handlar både om människor som står dig mycket nära, om vänner och om bekanta. Vissa frågor kan kanske kännas främmande, men försök att besvara dem ändå! Några frågor kan kännas väldigt lika, även om formuleringen är olika. Försök ändå att besvara dem så noggrant som möjligt.

Markera hur tillfredsställd du är med din situation i olika avseenden. Sätt ett kryss i rutan för den siffra som bäst anger hur du känner dig nu.

145. Hem- och familjesituation

mycket dåligt	☐ 1	☐ 2	☐ 3	☐ 4	☐ 5	☐ 6	☐ 7	alldeles utmärkt kunde inte vara bättre

146. Bostad

mycket dåligt	☐ 1	☐ 2	☐ 3	☐ 4	☐ 5	☐ 6	☐ 7	alldeles utmärkt kunde inte vara bättre

147. Arbetssituation

mycket dåligt	☐ 1	☐ 2	☐ 3	☐ 4	☐ 5	☐ 6	☐ 7	alldeles utmärkt kunde inte vara bättre

148. Ekonomi

mycket dåligt ☐ 1 ☐ 2 ☐ 3 ☐ 4 ☐ 5 ☐ 6 ☐ 7 alldeles utmärkt kunde inte vara bättre

149. Hälsa

mycket dåligt ☐ 1 ☐ 2 ☐ 3 ☐ 4 ☐ 5 ☐ 6 ☐ 7 alldeles utmärkt kunde inte vara bättre

150. Fritid

mycket dåligt ☐ 1 ☐ 2 ☐ 3 ☐ 4 ☐ 5 ☐ 6 ☐ 7 alldeles utmärkt kunde inte vara bättre

Man kan uppleva en del förändringar inom sig själv under årens lopp. Försök nedan markera (genom att kryssa i den rutan vars siffra bäst anger) hur du känner dig nu.

151. Hörseln

mycket dåligt ☐ 1 ☐ 2 ☐ 3 ☐ 4 ☐ 5 ☐ 6 ☐ 7 alldeles utmärkt kunde inte vara bättre

152. Synen

mycket dåligt ☐ 1 ☐ 2 ☐ 3 ☐ 4 ☐ 5 ☐ 6 ☐ 7 alldeles utmärkt kunde inte vara bättre

153. Minnet

mycket dåligt ☐ 1 ☐ 2 ☐ 3 ☐ 4 ☐ 5 ☐ 6 ☐ 7 alldeles utmärkt kunde inte vara bättre

154. Konditionen

mycket dåligt ☐ 1 ☐ 2 ☐ 3 ☐ 4 ☐ 5 ☐ 6 ☐ 7 alldeles utmärkt kunde inte vara bättre

155. Aptiten

mycket dåligt ☐ 1 ☐ 2 ☐ 3 ☐ 4 ☐ 5 ☐ 6 ☐ 7 alldeles utmärkt kunde inte vara bättre

156. Humöret

mycket dåligt ☐ 1 ☐ 2 ☐ 3 ☐ 4 ☐ 5 ☐ 6 ☐ 7 alldeles utmärkt kunde inte vara bättre

157. Energin

mycket dåligt ☐ 1 ☐ 2 ☐ 3 ☐ 4 ☐ 5 ☐ 6 ☐ 7 alldeles utmärkt kunde inte vara bättre

158. Tålamodet

mycket dåligt ☐ 1 ☐ 2 ☐ 3 ☐ 4 ☐ 5 ☐ 6 ☐ 7 alldeles utmärkt kunde inte vara bättre

159. Självförtroendet

mycket dåligt ☐ 1 ☐ 2 ☐ 3 ☐ 4 ☐ 5 ☐ 6 ☐ 7 alldeles utmärkt kunde inte vara bättre

160. Sömnen

mycket dålig	☐ 1	☐ 2	☐ 3	☐ 4	☐ 5	☐ 6	☐ 7	alldeles utmärkt kunde inte vara bättre

161. Känner du dig betydelsefull och uppskattad utanför hemmet?

inte alls	☐ 1	☐ 2	☐ 3	☐ 4	☐ 5	☐ 6	☐ 7	mycket

162. Känner du dig betydelsefull och uppskattad i hemmet?

inte alls	☐ 1	☐ 2	☐ 3	☐ 4	☐ 5	☐ 6	☐ 7	mycket

Har du under den senaste månaden ....

		ja	nej
163.	..haft minskad lust att göra saker som du vanligen tycker om	☐	☐
164.	..ofta känt dig illa till mods, deprimerad eller känt att framtiden ser hopplös ut	☐	☐
165.	..känt nervositet, ångest eller oro	☐	☐
166.	..känt ängslan över många olika saker	☐	☐
167.	.. haft en plötslig känsla av ångest eller panik	☐	☐

168. Hur många människor känner du och har kontakt med, som har samma intressen som du?
Det gäller kontakter både i arbetet och på fritiden

- ☐ ingen
- ☐ 1-2 personer
- ☐ 3-5 personer
- ☐ 6-10 personer
- ☐ 11-15 personer
- ☐ mer än 15 personer

169. Hur många människor, som du känner, träffar du eller samtalar du med, som du känner, träffar du eller samtalar du med under en vanlig vecka?
(Räkna ej med människor som du träffar tillfälligtvis och som du knappast kommer att återse, t.ex kunder i en affär)

- ☐ ingen
- ☐ 1-2 personer
- ☐ 3-5 personer
- ☐ 6-10 personer
- ☐ 11-15 personer
- ☐ mer än 15 personer

170. **Är det ungefär lagom många människor du träffar under ditt dagliga liv?**
Skulle du vilja träffa fler eller färre människor?

- ☐ färre
- ☐ lagom många
- ☐ fler

171. **Hur många vänner har du som kan komma hem till dig när som helst och känna sig hemma?** De skulle inte bry sig om, om det var ostädat eller om du höll på att äta. *(Nära släktingar skall inte räknas med!*

- ☐ ingen
- ☐ 1-2 personer
- ☐ 3-5 personer
- ☐ 6-10 personer
- ☐ 11-15 personer
- ☐ mer än 15 personer

172. **Hur många finns det, i din familj och bland dina vänner, som du kan tala öppet med utan att behöva tänka dig för?**

- ☐ ingen
- ☐ 1-2 personer
- ☐ 3-5 personer
- ☐ 6-10 personer
- ☐ 11-15 personer
- ☐ mer än 15 personer

173. **Finns det någon särskild person som du känner att du verkligen kan få stöd av?**

- ☐ ja
- ☐ ja, men jag behöver inte det.
- ☐ nej

174. **Finns det någon särskild person som känner sig stå väldigt nära dig?**

- ☐ ja
- ☐ är inte säker
- ☐ nej

175. **Har du någon särskild person som du kan dela dina känslor med när du känner dig lycklig?** *Någon som själv skulle känna sig lycklig bara för att du är det*

- ☐ ja
- ☐ nej

176. **Har du någon du kan dela dina innersta känslor med och anförtro dig åt?**

- ☐ ja
- ☐ nej

177. Händer det att någon håller om dig till tröst eller stöd?

- ☐ ja
- ☐ nej

178. Tror du att de där hemma eller några andra verkligen uppskattar vad du gör för dem?

- ☐ ja
- ☐ inte tillräckligt
- ☐ nej, inte alls

179. Finns det människor i din omgivning som du lätt kan be om saker, t.ex. människor som du känner så väl att du kan låna verktyg eller köksgrejor av?

- ☐ ja
- ☐ nej

180. Bortsett från dem där hemma, finns det någon som du kan vända dig till om du är i svårigheter? *Någon som du lätt kan träffa och som du litar på och kan få verklig hjälp av när du har det besvärligt?*

- ☐ ja
- ☐ nej

181. Har du under det senaste året deltagit i någon föreningsaktivitet, frivillig organisation etc. tillsammans med andra?(t ex sport, studiecirkel, teatergrupp, sångkör, politisk förening)?

- ☐ ja
- ☐ nej ► gå till fråga **184**

182. Hur ofta ägnar du dig åt föreningsaktivitet, klubbverksamhet studiecirkel etc. tillsammans med andra?

- ☐ 1-2 gånger per år
- ☐ 1-2 gånger per månad
- ☐ 1-2 gånger per vecka
- ☐ varje dag
- ☐ vet ej

183. Vilken/vilka föreningsaktiviteter deltar du i?

- ☐ sport och motion
- ☐ studiecirkel
- ☐ teatergrupp
- ☐ sångkör
- ☐ annan förening

184. **Har du under de senaste 12 månaderna gått på bio?**

- ☐ nej, ingen gång
- ☐ någon gång i kvartalet eller mer sällan (1-5 ggr/år)
- ☐ någon gång i månaden (6-20 ggr/år)
- ☐ någon gång i veckan eller oftare

185. **Har du under de senaste 12 månaderna gått på teater?**

- ☐ nej, ingen gång
- ☐ någon gång i kvartalet eller mer sällan (1-5 ggr/år)
- ☐ någon gång i månaden (6-20 ggr/år)
- ☐ någon gång i veckan eller oftare

186. **Har du under de senaste 12 månaderna gått på konserter och lyssnat på "levande" musik?**

- ☐ nej, ingen gång
- ☐ någon gång i kvartalet eller mer sällan (1-5 ggr/år)
- ☐ någon gång i månaden (6-20 ggr/år)
- ☐ någon gång i veckan eller oftare

187. **Har du under de senaste 12 månaderna gått på konstutställning/konstmuseum?**

- ☐ nej, ingen gång
- ☐ någon gång i kvartalet eller mer sällan (1-5 ggr/år)
- ☐ någon gång i månaden (6-20 ggr/år)
- ☐ någon gång i veckan eller oftare

188. **Har du under de senaste 12 månaderna gått på annat museum?**

- ☐ nej, ingen gång
- ☐ någon gång i kvartalet eller mer sällan (1-5 ggr/år)
- ☐ någon gång i månaden (6-20 ggr/år)
- ☐ någon gång i veckan eller oftare

189. **Har du under de senaste 12 månaderna gått på bibliotek?**

- ☐ nej, ingen gång
- ☐ någon gång i kvartalet eller mer sällan (1-5 ggr/år)
- ☐ någon gång i månaden (6-20 ggr/år)
- ☐ någon gång i veckan eller oftare

190. Har du under de senaste 12 månaderna gått på föreläsning/föredrag?

- ☐ nej, ingen gång
- ☐ någon gång i kvartalet eller mer sällan (1-5 ggr/år)
- ☐ någon gång i månaden (6-20 ggr/år)
- ☐ någon gång i veckan eller oftare

191. Har du under de senaste 12 månaderna spelat bingo (ej bingolotto)?

- ☐ nej, ingen gång
- ☐ någon gång i kvartalet eller mer sällan (1-5 ggr/år)
- ☐ någon gång i månaden (6-20 ggr/år)
- ☐ någon gång i veckan eller oftare

192. Har du under de senaste 12 månaderna gått på restaurang (pub, café) för nöjes skull?

- ☐ nej, ingen gång
- ☐ någon gång i kvartalet eller mer sällan (1-5 ggr/år)
- ☐ någon gång i månaden (6-20 ggr/år)
- ☐ någon gång i veckan eller oftare

Frågor rörande LIVSINSTÄLLNING

Följande frågor avser att mäta en del av motståndskraften mot sjukdom. Frågorna är således inte inriktade på vad man har för risker att bli sjuk, utan istället vilka tillgångar man har för att hantera påfrestningar. Varje fråga eller påstående har sju svarsalternativ. Var snäll och sätt kryss i den ruta som närmast uttrycker vad du känner. Lämna endast ett svar på varje fråga. Din omedelbara reaktion på frågan eller påståendet är det vi söker snarare än det du kommer fram till efter lång tids funderande.

193. Har du känslan av att du inte riktigt bryr dig om vad som händer omkring dig?

mycket sällan eller aldrig	☐ 1	☐ 2	☐ 3	☐ 4	☐ 5	☐ 6	☐ 7	mycket ofta

194. Har det hänt att du blivit överraskad av beteendet hos personer som du trodde du kände väl?

har aldrig hänt	☐ 1	☐ 2	☐ 3	☐ 4	☐ 5	☐ 6	☐ 7	händer alltid

195. Har det hänt att du blivit besviken på människor som du litat på?

har aldrig hänt	☐ 1	☐ 2	☐ 3	☐ 4	☐ 5	☐ 6	☐ 7	har hänt ofta

196. Hittills har ditt liv...

saknat tydligt mål och mening	☐ 1	☐ 2	☐ 3	☐ 4	☐ 5	☐ 6	☐ 7	haft klara mål och mening

197. Har du känslan av att du behandlas orättvist?

mycket ofta	☐	☐	☐	☐	☐	☐	☐	mycket sällan eller aldrig
	1	2	3	4	5	6	7	

198. Har du känslan av att du är i en ny situation utan att veta vad du skall göra?

mycket ofta	☐	☐	☐	☐	☐	☐	☐	mycket sällan eller aldrig
	1	2	3	4	5	6	7	

199. Är dina dagliga sysslor..

en källa till djup glädje eller tillfredsställelse	☐	☐	☐	☐	☐	☐	☐	en källa till smärta och leda
	1	2	3	4	5	6	7	

200. Har du mycket motstridiga känslor och idéer?

mycket ofta	☐	☐	☐	☐	☐	☐	☐	mycket sällan eller aldrig
	1	2	3	4	5	6	7	

201. Händer det att du har känslor inom dig som du helst inte vill känna?

mycket ofta	☐	☐	☐	☐	☐	☐	☐	mycket sällan eller aldrig
	1	2	3	4	5	6	7	

202. Många människor - även de med stark självkänsla - kan känna sig helt misslyckade i vissa situationer. Hur ofta har du känt så?

aldrig	☐	☐	☐	☐	☐	☐	☐	mycket ofta
	1	2	3	4	5	6	7	

203. När någonting har hänt, har du i allmänhet upptäckt att du ..

över- eller undervärderar betydelsen av vad som hände	☐	☐	☐	☐	☐	☐	☐	såg saker i dess rätta proportioner
	1	2	3	4	5	6	7	

204. Hur ofta känner du att det inte finns någon mening med dina dagliga sysslor?

mycket ofta	☐	☐	☐	☐	☐	☐	☐	mycket sällan eller aldrig
	1	2	3	4	5	6	7	

205. Hur ofta har du känslor som du är osäker på att kunna behärska?

mycket ofta	☐	☐	☐	☐	☐	☐	☐	mycket sällan eller aldrig
	1	2	3	4	5	6	7	

Nedan beskriver vi ett antal känslor som alla människor kan uppleva då och då. Var vänlig och ange i vilken mån dessa känslor vanligtvis förekommer hos dig.

206. Jag känner mig trött

nästan aldrig	☐	☐	☐	☐	☐	☐	☐	nästan alltid
	1	2	3	4	5	6	7	

207. Jag känner mig pigg

nästan aldrig ☐ 1 ☐ 2 ☐ 3 ☐ 4 ☐ 5 ☐ 6 ☐ 7 nästan alltid

208. Jag känner mig fysiskt utmattad

nästan aldrig ☐ 1 ☐ 2 ☐ 3 ☐ 4 ☐ 5 ☐ 6 ☐ 7 nästan alltid

209. Jag känner att jag har fått nog

nästan aldrig ☐ 1 ☐ 2 ☐ 3 ☐ 4 ☐ 5 ☐ 6 ☐ 7 nästan alltid

210. Jag känner mig full av energi

nästan aldrig ☐ 1 ☐ 2 ☐ 3 ☐ 4 ☐ 5 ☐ 6 ☐ 7 nästan alltid

211. Mina "batterier" är uttömda

nästan aldrig ☐ 1 ☐ 2 ☐ 3 ☐ 4 ☐ 5 ☐ 6 ☐ 7 nästan alltid

212. Jag känner mig alert

nästan aldrig ☐ 1 ☐ 2 ☐ 3 ☐ 4 ☐ 5 ☐ 6 ☐ 7 nästan alltid

213. Jag känner mig utbränd

nästan aldrig ☐ 1 ☐ 2 ☐ 3 ☐ 4 ☐ 5 ☐ 6 ☐ 7 nästan alltid

214. Jag känner mig mentalt trött

nästan aldrig ☐ 1 ☐ 2 ☐ 3 ☐ 4 ☐ 5 ☐ 6 ☐ 7 nästan alltid

215. Jag känner att jag inte orkar gå till arbetet på morgonen

nästan aldrig ☐ 1 ☐ 2 ☐ 3 ☐ 4 ☐ 5 ☐ 6 ☐ 7 nästan alltid

216. Jag känner mig aktiv

nästan aldrig ☐ 1 ☐ 2 ☐ 3 ☐ 4 ☐ 5 ☐ 6 ☐ 7 nästan alltid

217. Jag känner mig dåsig

nästan aldrig ☐ 1 ☐ 2 ☐ 3 ☐ 4 ☐ 5 ☐ 6 ☐ 7 nästan alltid

218. Jag känner mig spänd

nästan aldrig ☐ 1 ☐ 2 ☐ 3 ☐ 4 ☐ 5 ☐ 6 ☐ 7 nästan alltid

219. Jag känner mig avspänd

nästan aldrig ☐ 1 ☐ 2 ☐ 3 ☐ 4 ☐ 5 ☐ 6 ☐ 7 nästan alltid

220. Jag känner mig rastlös

nästan aldrig ☐ 1 ☐ 2 ☐ 3 ☐ 4 ☐ 5 ☐ 6 ☐ 7 nästan alltid

221. Jag känner stark inre spänning

nästan aldrig ☐ 1 ☐ 2 ☐ 3 ☐ 4 ☐ 5 ☐ 6 ☐ 7 nästan alltid

222. Jag känner mig för trött för att tänka

nästan aldrig ☐ 1 ☐ 2 ☐ 3 ☐ 4 ☐ 5 ☐ 6 ☐ 7 nästan alltid

223. Jag har svårt att koncentrera mig

nästan aldrig ☐ 1 ☐ 2 ☐ 3 ☐ 4 ☐ 5 ☐ 6 ☐ 7 nästan alltid

224. Jag känner mig trögtänkt

nästan aldrig ☐ 1 ☐ 2 ☐ 3 ☐ 4 ☐ 5 ☐ 6 ☐ 7 nästan alltid

225. Jag kan inte tänka klart

nästan aldrig ☐ 1 ☐ 2 ☐ 3 ☐ 4 ☐ 5 ☐ 6 ☐ 7 nästan alltid

226. Det känns svårt att tänka på komplicerade saker

nästan aldrig ☐ 1 ☐ 2 ☐ 3 ☐ 4 ☐ 5 ☐ 6 ☐ 7 nästan alltid

227. Jag känner mig splittrad i tankarna

nästan aldrig ☐ 1 ☐ 2 ☐ 3 ☐ 4 ☐ 5 ☐ 6 ☐ 7 nästan alltid

HJÄRTLIGT TACK FÖR DIN MEDVERKAN !
