# Supplementary material for: Time trends of comparative self-rated health in adults aged 25-34 in the Northern Sweden MONICA study, 1990-2014
Source: PLoS One. 2017 Nov 20;12(11):e0187896. doi: 10.1371/journal.pone.0187896 (PMC5695772; doi:10.1371/journal.pone.0187896)
Supplement: S5 Text — (PDF) [file pone.0187896.s005.pdf]

Personnummer:

Namn:

MONICA-nummer:

Norra Sveriges MONICA-undersökning

En kampanj mot

hjärt-kärlsjukdom och diabetes

2009

FRÅGEFORMULÄR DEL 1

INFORMATION

Det frågeformulär som vi nu ber dig att besvara, innehåller frågor rörande bl.a. hälsa, arbete, fysisk aktivitet, rök-, kost- och motionsvanor.
Alla uppgifter som du lämnar kommer att behandlas konfidentiellt. Resultaten kommer att sammanställas på sådant sätt att det inte går att identifiera enskilda individer.
Om det är något du funderar över angående frågeformulären eller undersökningen, kontakta gärna någon av oss. Du når oss säkrast på telefon på förmiddagarna.
Om vi skulle få problem med att tolka något av dina ifyllda svar, skulle vi vara mycket tacksamma om vi får ringa dig eller skicka e-post för att reda ut det som är oklart. Därför är det bra om du anger det telefonnummer där vi lättast kan nå dig (helst dagtid) och eventuell e-post-adress.

Så här fyller du i enkäten

Att besvara enkäten tar ungefär en halv timme. Enkätsvaren registreras maskinellt, därför är det viktigt att de är ifyllda på rätt sätt.

- Använd en bra penna med **svart eller mörkblå färg**. Undvik blyertspenna.
- Sätt ett kryss i de rutor du tycker stämmer bäst för dig. Markera om möjligt inom rutorna.

Så här: ☒

- Om du råkar kryssa fel fyller du i hela den rutan med färg.

Så här: ■

Då kan inte maskinen läsa den. Kryssa sedan i den rätta rutan.

Vi ber dig att noga läsa igenom frågorna och svarsalternativen innan du besvarar alla frågorna.

Mitt telefonnummer dagtid är: ____________

Jag träffas säkrast:

Efter klockan	Fram till klockan

E-postadress: ____________

Britt-Inger Eklund
Forskningssjuksköterska
MONICA-projektet
Medicinkliniken
Norrlands Universitetssjukhus
901 85 UMEÅ
Tel: 090 - 785 87 82
Epost:brittinger.eklund@vll.se

Karin Ruikka
Forskningssjuksköterska
MONICA-projektet
Björkskatans vårdcentral
Höstvägen 7
976 27 LULEÅ
Tel: 0920 - 719 47
Epost: karin.ruikka@nll.se

Endast ett X på varje fråga där inte annat anges

Frågor rörande CIVILSTÅND och BOENDE

1. Vilket är ditt nuvarande civilstånd?

- ☐ ogift
- ☐ gift eller sammanboende
- ☐ skild eller separerad
- ☐ änka/änkling
- ☐ annat____

2. Vilka personer sammanbor du med?

- ☐ vuxen (make, maka, sambo)
- ☐ barn, ange antal [ ]
- ☐ vuxen och barn, ange antal [ ]
- ☐ annan
- ☐ bor helt ensam

3. Var bor du?

- ☐ större tätort - Umeå, Skellefteå, Luleå, Boden, Piteå, Kiruna, orter med än 15 000 invånare
- ☐ annan tätort med mer än 1 000 invånare
- ☐ ort med mindre än 1 000 invånare

4. I vilket land är du född?

- ☐ Sverige ➔ gå till fråga **5**
- ☐ Finland ➔ gå till fråga **6**
- ☐ annat Skandinaviskt land ➔ gå till fråga **6**
- ☐ övriga Europa ➔ gå till fråga **6**
- ☐ annat utomeuropeiskt land ➔ gå till fråga **6**

5. Var är du född? *(Ange vilken kyrkoförsamling eller ort du tillhörde vid födseln)?*

Församling	Ort	län

6. **Vilken är den högsta utbildningsnivå som du har avslutat?**

- ☐ folkskola
- ☐ grundskola
- ☐ folkhögskola motsvarande grundskolekompetens
- ☐ yrkesskola
- ☐ realskola
- ☐ flickskola
- ☐ fackskola
- ☐ gymnasieskola
- ☐ folkhögskola motsvarande gymnasiekompetens
- ☐ högskola eller universitet

7. **Hur många år har du gått i skola eller ägnat dig åt studier på heltid?**
(Ange antal år från första klass och framåt) ☐ **år**

*Frågor rörande TOBAKSVANOR*

8. **Röker du <u>cigaretter</u> för närvarande?**

- ☐ ja, regelbundet (1 cigarett eller mer per dag) ➔ gå till fråga **9**
- ☐ nej ➔ gå till fråga **12**
- ☐ ibland (mindre än 1 cigarett per dag) ➔ gå till fråga **10**

9. **Ungefär hur många cigaretter röker du i genomsnitt per dag?**

(Ange antal cigaretter/dag) ☐ ➔ gå till fråga **16**

10. **Hur många dagar i veckan röker du cigaretter?**

- ☐ vanligtvis en dag eller mindre
- ☐ vanligtvis 2 till 4 dagar
- ☐ nästan varje dag

11. **Ungefär hur många cigaretter röker du i genomsnitt per vecka?**

(Ange antal cigaretter/vecka) ☐

12. **Har du någonsin rökt cigaretter regelbundet tidigare?**

- ☐ ja, regelbundet (1 cigarett eller mer per dag) ➔ gå till fråga **13**
- ☐ nej ➔ gå till fråga **18**

13. När slutade du att röka cigaretter regelbundet?

(Ange årtal) ☐

14. Om du slutade röka under det senaste året, när slutade du?

☐ för mindre än en månad sedan

☐ för 1-6 månader sedan

☐ för 7-12 månader sedan

15 Vilken är den främsta orsaken till att du slutat röka? *(Ange endast **ett** alternativ)*

☐ av hälsoskäl, på eget initiativ

☐ på inrådan av läkare/sjukvårdspersonal

☐ pga. annan information/upplysning

☐ pga. tryck från kamrater/familjemedlemmar

☐ av andra skäl

16. Vilket är det högsta antal cigaretter/dag, som du rökt under en så lång period som ett år?

(Ange *antal cigaretter/dag)* ☐

17. Hur gammal var du när du började röka cigaretter?

(Ange ålder i år) ☐

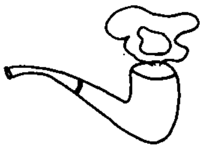

18. Hur du någonsin rökt <u>pipa?</u>

☐ ja, för närvarande regelbundet ➔ gå till fråga **19**

☐ nej ➔ gå till fråga **20**

☐ jag röker pipa ibland (mindre än 1 gång/dag) ➔ gå till fråga **19**

☐ tidigare, men inte nu ➔ gå till fråga **20**

19. Hur många gram tobak röker du per vecka?

Ett paket piptobak väger som regel 50 gram *(Ange antal gram/vecka)* ☐

20. Hur du någonsin rökt <u>cigarrer eller cigariller</u>

☐ ja, för närvarande regelbundet ➔ gå till fråga **21**

☐ nej ➔ gå till fråga **22**

☐ jag röker ibland (mindre än 1 /dag) ➔ gå till fråga **21**

☐ tidigare, men inte nu ➔ gå till fråga **22**

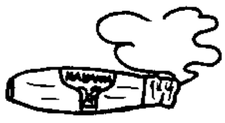

21. **Ungefär hur många cigarrer/cigariller röker du i genomsnitt per vecka?**

(Ange antal cigarrer/cigariller/vecka) ☐

OBS! Fråga 22 skall endast fyllas i av icke-rökare och de som röker ibland

22. **Hur många timmar i medeltal per dag, är du i närheten av andra människors tobaksrök?**

(Ange antal timmar/dag du inandas eller känner lukten av tobaksrök) ☐

23. **Har du någonsin använt snus?**

☐ ja, snusade tidigare men inte nu

☐ ja, snusar mindre än 2 dosor per vecka

☐ ja, snusar 2 - 4 dosor per vecka

☐ ja, snusar mer än 4 men mindre än 7 dosor per vecka

☐ ja, snusar 7 dosor eller fler per vecka

☐ nej ➔ gå till fråga **26**

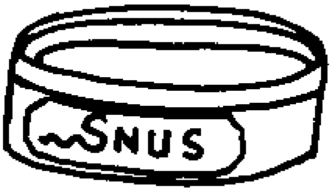

24. **Hur många år har du använt snus?**

(Ange antal år) ☐

25. **Började du att snusa i samband med att du slutade röka?**

☐ ja

☐ nej

☐ jag både röker och snusar

26. **Använder du dagligen <u>andra</u> nikotinprodukter?**

☐ ja, tuggtobak

☐ ja, nikotinersättningsmedel (nikotintuggummi, nikotinplåster, nikotinnässpray, nikotinsugtabletter)

☐ annat____

☐ nej

___________Frågor rörande HÄLSA ___________

27. **Har du vid något tillfälle av läkare eller annan sjukvårds personal fått besked om att du har högt blodtryck?**

☐ ja

☐ nej ➔ gå till fråga **29**

28. **Har du under de senaste 2 veckorna tagit läkemedel mot förhöjt blodtryck?**

- ☐ ja
- ☐ nej
- ☐ osäker på om den medicin jag äter är mot högt blodtryck

29. **Har du fått ditt blodtryck mätt under det senaste året?**

- ☐ ja
- ☐ nej

30. **Har eller hade någon av dina föräldrar behandling för högt blodtryck?**

- ☐ ja, min pappa
- ☐ ja, min mamma
- ☐ ja, båda föräldrarna
- ☐ nej, ingen av föräldrarna
- ☐ vet ej

31. **Har du vid något tillfälle av läkare eller annan sjukvårdspersonal fått besked om att du har förhöjda kolesterol-/blodfettvärden?**

- ☐ ja
- ☐ nej

32. **Har läkare eller annan sjukvårdspersonal föreskrivet en särskild diet till dig för att sänka dina kolesterol-/blodfettnivåer?**

- ☐ ja
- ☐ nej
- ☐ håller en föreskriven diet, men vet ej om den är till för att sänka mina kolesterol-/blodfettnivåer

33. **Har du under de senaste två veckorna tagit medicin, föreskriven av läkare för att sänka kolesterol-/blodfettvärdet?**

- ☐ ja
- ☐ nej
- ☐ vet ej om medicinen jag äter är för att sänka kolesterolet

34. **Har du fått ditt kolesterol/blodfett mätt under det senaste året?**

- ☐ ja
- ☐ nej

35. **Har du under de senaste två veckorna tagit acetylsalicylika t ex Albyl, Aspirin, Bamyl, Bamycor, Trombyl, Magnecyl, Asasantin Retard för att förebygga eller behandla hjärtsjukdom?**

☐ ja

☐ nej

☐ nej, men jag använder acetylsalicylika regelbundet för att behandla annan sjukdom än hjärtsjukdom

36. **Har du under de senaste två veckorna tagit acetylsalicylika t ex Albyl, Aspirin, Bamyl, Bamycor, Trombyl, Magnecyl, Asasantin Retard för att förebygga eller behandla slaganfall (propp i hjärnan)?**

☐ ja

☐ nej

☐ nej, men jag använder acetylsalicylika regelbundet för att behandla annan sjukdom än slaganfall

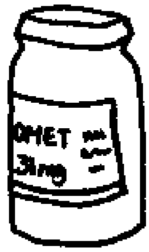

37. **Har du under de senaste 14 dagarna använt något läkemedel som ordinerats av läkare?**

☐ ja

☐ nej

38. **Om du svarat "ja" i så fall vilken eller vilka mediciner?**

1 ______________________ 4 ______________________

2 ______________________ 5 ______________________

3 ______________________ 6 ______________________

39. **Har du under de senaste 14 dagarna använt någon annan medicin?**
(T.ex. naturmedicin, vitamin- eller järnpreparat, förebyggande medicin)

☐ ja

☐ nej

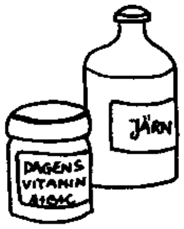

40. **Om du svarat "ja" i så fall vilken eller vilka mediciner?**

1 ______________________ 4 ______________________

2 ______________________ 5 ______________________

3 ______________________ 6 ______________________

41. **Får du smärtor - stickningar - ont i bröstet när du går uppför backar eller trappor, eller när du går fort på plan mark?**

☐ ja

☐ nej

42. **Får du smärtor - stickningar - ont i bröstet när du går i vanlig takt på plan mark?**

☐ ja

☐ nej

Om** du svarat **nej** på både fråga 41 och 42 gå till fråga **47

43. **Om du får smärtor eller obehag i bröstet i samband med att du rör dig, brukar du då?**

☐ stanna

☐ sakta ner farten

☐ fortsätta i samma takt

44. **Om du stannar eller saktar ner, försvinner smärtorna då?**

☐ ja

☐ nej

45. **Om de försvinner, hur snart försvinner de?**

☐ efter mindre än 10 minuter

☐ efter mer än 10 minuter

46. **VAR BRUKAR DU KÄNNA DESSA SMÄRTOR ELLER OBEHAG?**

(*Markera med X på figuren där du känner smärta eller obehag. Om smärta eller obehag känns ut i armarna eller upp mot halsen, markera med X även där!)*

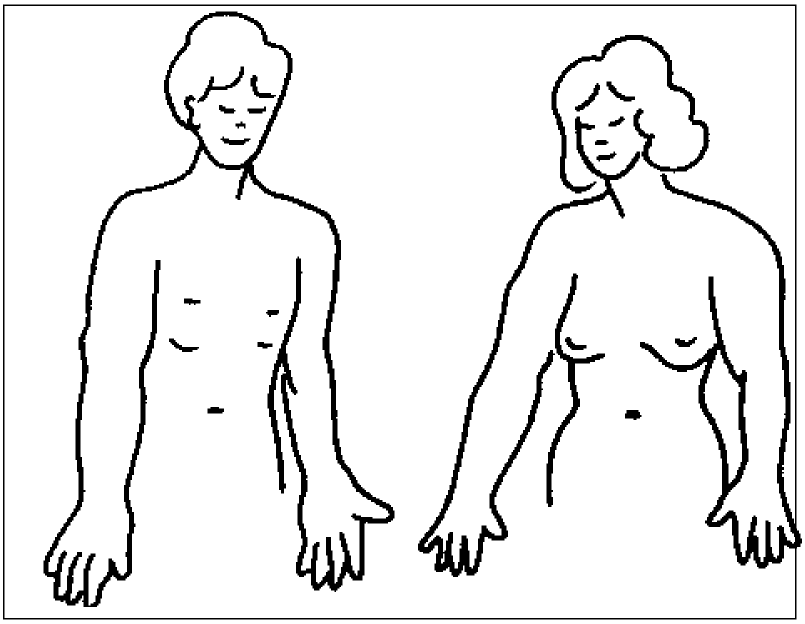

47. Har du någon gång haft svår smärta i bröstet som varat i en halvtimme eller mer?

- ☐ ja
- ☐ nej

48. Har du legat på sjukhus för säker hjärtinfarkt (propp i hjärtat)**?**

- ☐ ja, ange årtal [ ] och sjukhus ____________________
- ☐ nej
- ☐ vet ej om det var en säker hjärtinfarkt

49. Brukar du få smärtor i vaderna, när du går i uppförsbackar, trappor eller på plan mark?

- ☐ ja
- ☐ nej

50. Blir du andfådd av att gå två trappor upp eller motsvarande i samma takt som jämnåriga?

- ☐ ja
- ☐ nej

51. Har du haft slaganfall (hjärnblödning eller propp i hjärnan)?

- ☐ ja, ange årtal [ ] och sjukhus ____________________
- ☐ nej

52. Har du diabetes/sockersjuka?

- ☐ ja
- ☐ nej ➔ gå till fråga **56**

53. Hur behandlar du din diabetes/sockersjuka?

- ☐ insulin
- ☐ tabletter
- ☐ både insulin och tabletter
- ☐ enbart kost

54. Hur gammal var du när du fick diabetes/sockersjuka?

[ ] år

55. Om du behandlas med insulin, vilket år började du behandlas med insulin ?

[ ]

56. **Finns det någon i din släkt som har/har haft diabetes/sockersjuka (***Flera alternativ är möjliga).* Med släkt menas i detta fall: Föräldrar, syskon och barn

☐ ja, min mamma

☐ ja, min pappa

☐ ja, båda föräldrarna

☐ ja, något/några syskon

☐ ja, något/några barn

☐ nej

☐ vet ej

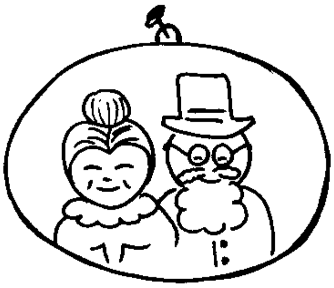

57. **Har du mångåriga magbesvär?**

☐ ja

☐ nej

Flera alternativ är möjliga!

58. **Finns det någon i din släkt som avlidit i hjärtinfarkt före 65 års ålder?** Med släkt menas i detta fall: Föräldrar, syskon, fastrar, farbröder, mostrar och morbröder.

☐ ja, min mamma	Hon var då		år
☐ ja, min pappa	Han var då		år
☐ ja, något/några syskon			år
☐ ja, någon/några av fastrar, farbröder, mostrar och morbröder.			år
☐ nej.			år
☐ vet ej			år

59. **Finns det någon i din släkt som avlidit i slaganfall (propp eller blödning i hjärnan) före 65 års ålder?** Med släkt menas i detta fall: Föräldrar, syskon, fastrar, farbröder, mostrar och morbröder

☐ ja, min mamma	Hon var då		år
☐ ja, min pappa	Han var då		år
☐ ja, något/några syskon			år
☐ ja, någon/några av fastrar, farbröder, mostrar och morbröder.			år
☐ nej.			år
☐ vet ej			år

Endast ett X på varje fråga där inte annat anges

60. **Har du vid något tillfälle av läkare eller annan sjukvårdsperson fått besked om att du har lungsjukdomen KOL?**

☐ ja

☐ nej

61. **Har du haft pip eller har det väst i bröstet vid något tillfälle under de senaste 12 månaderna?**

☐ ja

☐ nej ➔ Gå till fråga **64**

62. **Har du överhuvudtaget varit det minsta andfådd när du haft detta pip eller väsande ljud i bröstet?**

☐ ja

☐ nej

63. **Har du haft detta pip eller väsande i bröstet när du <u>inte</u> samtidigt varit förkyld?**

☐ ja

☐ nej

64. **Har du av läkare fått diagnosen astma?**

☐ ja

☐ nej

65. **Hostar eller harklar du upp slem (eller har slem som det är svårt att få upp, trots hosta) de flesta dagar i perioder om minst 3 månader per år?**

☐ ja, ange under hur många år [ ] år

☐ nej

66. **Vaknar du nattetid på grund av andfåddhet?**

☐ ja, varje natt

☐ ja, en gång per vecka eller mer

☐ ja, mindre än en gång per vecka (1-3 ggr/mån)

☐ nej

67. **Har du genomgått ballongvidgning av hjärtats blodkärl eller kranskärlsoperation?**

☐ ja

☐ nej

☐ vet ej

68. Är du opererad för klaffel i hjärtat?

- ☐ ja
- ☐ nej
- ☐ vet ej

69. Kontrolleras eller behandlas du av läkare för reumatiska led- eller muskelbesvär i form av smärtor/värk, stelhet? *Flera alternativ är möjliga*

- ☐ ja muskelbesvär
- ☐ ja, ledbesvär
- ☐ nej

70. Har du under de sista 10 åren använt cortisonpreparat i tablettform mer än 3 månader?

- ☐ ja
- ☐ nej

71. Har någon av dina föräldrar, efter 50 års ålder, drabbats av höftfraktur (lårbenshalsbrott)?

- ☐ ja, mor
- ☐ ja, far
- ☐ ja, både mor och far
- ☐ nej

72. Har du vid/efter 40 års ålder behandlats för fraktur (benbrott) på någon del av kroppen??

- ☐ ja
- ☐ nej ➔ gå till fråga **74**
- ☐ nej, ej aktuellt, är inte 40 år ➔ gå till fråga **74**

73. Markera vilken eller vilka frakturer (benbrott) du har behandlats för vid/efter 40 års ålder. *Flera alternativ är möjliga.*

- ☐ handledsfraktur
- ☐ höftfraktur (lårbenshalsbrott)
- ☐ överarmsfraktur eller axelfraktur
- ☐ fraktur på ryggkota
- ☐ knäfraktur
- ☐ fotledsfaktur
- ☐ fraktur på annat ställe

74. **Äter du eller har du ätit något eller några av följande kosttillskott?** *Båda alternativen är möjliga.*

☐ kalk/kalcium

☐ vitamin D

75. **Har du någon gång av läkare fått diagnosen sorkfeber?**

☐ ja

☐ nej ➔ Gå till fråga **77**

76. **Vilket år fick du diagnosen sorkfeber?**

[ ]

77. **Snarkar du när du sover?**

☐ ja, alltid

☐ ja, nästan alltid

☐ ja, ibland

☐ nej, nästan aldrig

☐ nej, aldrig

☐ vet inte om jag snarkar

78. **Har din make/maka/sambo märkt att du har andningsuppehåll under sömn?**

☐ ja, i stort sett varje natt

☐ ja, ofta

☐ ja, ibland

☐ nej, nästan aldrig

☐ nej, aldrig

☐ vet inte

79. **Besväras du av trötthet under dagen?**

☐ ja, alltid

☐ ja, nästan alltid

☐ ja, ibland

☐ nej, nästan aldrig

☐ nej, aldrig

80. **Hur är det kaffe du dricker vanligtvis tillagat?**

- ☐ jag dricker mest kokkaffe
- ☐ jag dricker mest bryggkaffe
- ☐ annat (t ex snabbkaffe)
- ☐ jag dricker mest kaffe tillagat i s.k. perkulator
- ☐ jag dricker inte kaffe ➔ gå till fråga **82**

81. **Hur många koppar kaffe dricker du i genomsnitt varje dag?**
(Ange antal kaffekoppar) ☐

82. **Vilket sorts dricksvatten använder du huvudsakligen?**

- ☐ kommunalt vatten
- ☐ egen brunn ➔ Hur många år har ni använt vatten från denna brunn?
  - ☐ mindre än 5 år
  - ☐ 5-10 år
  - ☐ mer än 10år
- ☐ lokal vattenförening eller liknande

83. **Hur ofta äter du lokalt fiskad fisk (från insjöar, älvar, kustvattenområden)?**

- ☐ aldrig
- ☐ någon gång per år
- ☐ 1 gång varannan månad
- ☐ 1 gång per månad
- ☐ 2 ggr per månad
- ☐ 1 gång per vecka
- ☐ 2 - 3 ggr per vecka
- ☐ 1 gång per dag eller mer

Frågor om ARBETE

Med arbete avses din huvudsakliga sysselsättning.
OBS: Ange endast ett alternativ!

84. Hur är dina nuvarande arbetsförhållanden?

- ☐ fast anställning, anställning tills vidare
- ☐ egen företagare
- ☐ tillfällig anställning, vikariat, beredskapsarbete
- ☐ arbetar i hemmet
- ☐ arbetslös ➔ gå till fråga **107**
- ☐ studerande ➔ gå till fråga **105**
- ☐ pensionerad ➔ gå till fråga **106**

85. Hur många timmar förvärvsarbetar du i normala fall per vecka?

- ☐ jag förvärvsarbetar inte
- ☐ mindre än 15 timmar
- ☐ 15-35 timmar
- ☐ mer än 35 timmar

86. Vad har du för arbetstider i normala fall?

- ☐ jag förvärvsarbetar inte
- ☐ fast arbetstid (t ex 7-16 eller 8-17)
- ☐ skiftarbete (t ex 2-skift, 3-skift, 5-skift)
- ☐ varierande arbetstider (ibland dagtid, ibland kvällstid, ibland helger)

87. Är ditt arbete fysiskt (kroppsligt) tungt?

- ☐ ja, ofta
- ☐ ja, ibland
- ☐ nej, sällan
- ☐ nej, så gott som aldrig

88. Kräver ditt arbete att du arbetar mycket fort?

- ☐ ja, ofta
- ☐ ja, ibland
- ☐ nej, sällan
- ☐ nej, så gott som aldrig

89. Är ditt arbete psykiskt påfrestande?

- ☐ ja, ofta
- ☐ ja, ibland
- ☐ nej, sällan
- ☐ nej, så gott som aldrig

90. Har du tillräckligt med tid för att hinna med arbetsuppgifterna?

- ☐ ja, ofta
- ☐ ja, ibland
- ☐ nej, sällan
- ☐ nej, så gott som aldrig

91. Förekommer det ofta motstridiga krav i ditt arbete?

- ☐ ja, ofta
- ☐ ja, ibland
- ☐ nej, sällan
- ☐ nej, så gott som aldrig

92. Får du lära dig nya saker i ditt arbete?

- ☐ ja, ofta
- ☐ ja, ibland
- ☐ nej, sällan
- ☐ nej, så gott som aldrig

93. Kräver ditt arbete skicklighet?

- ☐ ja, ofta
- ☐ ja, ibland
- ☐ nej, sällan
- ☐ nej, så gott som aldrig

94. Kräver ditt arbete påhittighet?

- ☐ ja, ofta
- ☐ ja, ibland
- ☐ nej, sällan
- ☐ nej, så gott som aldrig

95. Innebär ditt arbete att man gör samma sak om och om igen?

- ☐ ja, ofta
- ☐ ja, ibland
- ☐ nej, sällan
- ☐ nej, så gott som aldrig

96. Har du frihet att bestämma hur ditt arbete skall utföras?

- ☐ ja, ofta
- ☐ ja, ibland
- ☐ nej, sällan
- ☐ nej, så gott som aldrig

97. Har du frihet att bestämma vad som skall utföras i ditt arbete?

- ☐ ja, ofta
- ☐ ja, ibland
- ☐ nej, sällan
- ☐ nej, så gott som aldrig

98. Har du i vanliga fall möjlighet att tala med dina arbetskamrater under raster, om du skulle vilja det?

- ☐ ja, alltid
- ☐ ja, för det mesta
- ☐ nej, jag har inga raster
- ☐ nej, jag har inga raster tillsammans med arbetskamrater

99. Är ditt arbete av den karaktären att du kan lämna det ett tag om du vill tala med en arbetskamrat?

- ☐ ja, för det mesta
- ☐ ja, ibland
- ☐ bara för brådskande ärenden
- ☐ nej, det är helt omöjlig

100. Har du, som en del i ditt arbete, ofta kontakt med dina arbetskamrater?

- ☐ ja, ständigt kontakt med arbetskamrater i arbetet
- ☐ ja, ibland kontakt med arbetskamrater i arbetet
- ☐ nej, jag arbetar för det mesta ensam
- ☐ nej, jag arbetar ständigt ensam

101. **Hur ofta är du vanligen tillsammans med en eller flera av dina arbetskamrater på fritiden?**

- ☐ en eller flera ggr/vecka
- ☐ en eller ett par ggr/månad
- ☐ en eller ett par ggr/år
- ☐ sällan eller aldrig

102. **När besökte någon av dina arbetskamrater dig senast?**

- ☐ för en till fyra veckor sedan
- ☐ för en till tolv månader sedan
- ☐ för mer än ett år sedan
- ☐ har aldrig haft besök av någon arbetskamrat

103. **Tror du att det finns risk att du blir arbetslös?**

- ☐ ja, inom den närmaste tiden
- ☐ nej

104. **Antag att du har ett arbete och blir uppsagd. Hur stora chanser tror du att du har att få ett nytt arbete inom en månad?**

- ☐ mycket stora
- ☐ ganska stora
- ☐ små
- ☐ inga chanser

105. **På vilken nivå studerar du?** *(Denna fråga besvaras endast av studerande)*

- ☐ grundskolenivå
- ☐ gymnasial nivå
- ☐ eftergymnasial nivå
- ☐ annan, vilken ____________________

106. **Har du varit arbetslös någon gång?**

- ☐ ja, för mer än 12 månader sedan
- ☐ ja, någon gång under de senaste 12 månaderna
- ☐ nej, aldrig ➔ gå till fråga **108**

107. Om du varit/eller är arbetslös, hur länge har du sammanlagt varit det?

- ☐ mindre än 3 månader
- ☐ 3-12 månader
- ☐ mer än 12 månader

108. Har du tvingats flytta någon gång?

- ☐ ja, för mer än 12 månader sedan
- ☐ ja, de senaste 12 månaderna
- ☐ nej aldrig ➔ gå till fråga **110**

109. Vilken var anledningen till flyttningen?

- ☐ familjeskäl
- ☐ arbetsmarknadsskäl
- ☐ annat

110. Har någon eller några personer som betytt eller betyder mycket för dig, flyttat så att du inte längre kan umgås med honom/henne eller dem?

- ☐ ja
- ☐ nej ➔ gå till fråga **112**

111. Om du svarat "ja" på föregående fråga, vilken var anledningen till flyttningen?

- ☐ familjeskäl
- ☐ arbetsmarknadsskäl
- ☐ annat

112. Är du just nu sjukskriven, har ålderspension, har förtidspension, har sjukpension eller sjukbidrag?

- ☐ ja, ålderspension/sjukpension på heltid
- ☐ ja, ålderspension/sjukpension på deltid
- ☐ ja, förtidspension/sjukbidrag
- ☐ ja, sjukskriven
- ☐ nej

113. **Vilket är ditt yrke/din befattning?** Om du inte förvärvsarbetar nu ska du ange yrke/sysselsättning som du tidigare huvudsakligen haft
(Undvik allmänna yrkesbeteckningar som lärare, tjänsteman, byggnadsarbetare etc. Skriv i stället högstadielärare, kontorist, byggnadssnickare etc. så att det framgår mer exakt vad du arbetar/arbetade med).

114. **Beskriv kortfattat dina arbetsuppgifter, samt ange arbetsplats eller typ av företag.**

Besvaras av gifta/samboende

115. **Vilket är din make/makas/samboendes yrke eller befattning?**
Om han/hon inte förvärvsarbetar nu ska du ange den yrke/sysselsättning som han/hon tidigare huvudsakligen haft)

116. **Beskriv kortfattat hans/hennes arbetsuppgifter**

Besvaras av de som är egna företagare

117. **Hur många anställda har du i ditt företag?**

Antal anställda [ ]

118. **Är ditt jordbruk/lantbruk att betrakta som ett litet eller stort jordbruk.**
Besvaras endast av jordbrukare *(Kryssa för det alternativ som stämmer bäst med din situation, samt ange hur mycket skog och åkermark du har)*

- ☐ litet jordbruk. hektar skog [ ] och hektar åkermark [ ]
- ☐ stort jordbruk. hektar skog [ ] och hektar åkermark [ ]

Frågor om FYSISK AKTIVITET

Sätt bara* X *i ett av alternativen

119. Hur mycket har du rört dig eller ansträngt dig kroppsligt på din <u>fritid</u> under det senaste året?

☐ **knappast något alls**

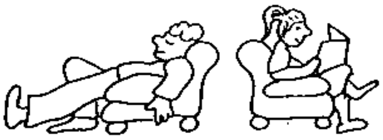

☐ **mestadels stillasittande, ibland någon promenad eller liknande.**

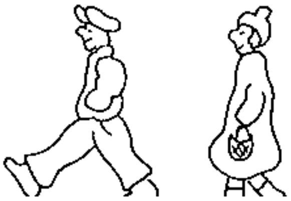

☐ **lättare fysisk ansträngning <u>minst 2 timmar i veckan</u>,**
t.ex. gång och cykling (även till och från arbetet eller skolan) fiske, dans etc.

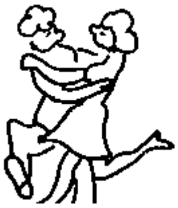

☐ **mer ansträngande motion <u>1-2 timmar i veckan</u>,**
t.ex. motionslöpning, tennis, simning, badminton, motionsgymnastik.

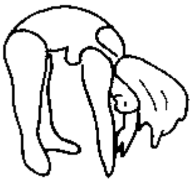

☐ **mer ansträngande motion <u>minst 3 timmar i veckan,</u>**
t.ex. motionslöpning, tennis, simning, badminton, motionsgymnastik

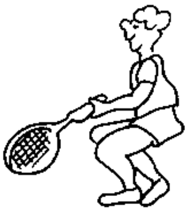

☐ **<u>hård träning eller tävling</u> regelbundet och <u>flera gånger i veckan</u>** där den fysiska ansträngningen är stor
t.ex. löpning, skidåkning, fotboll, simning

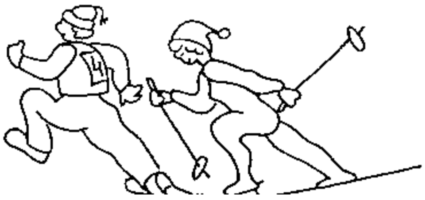

Sätt bara X i ett av alternativen

120. **Hur mycket har du rört dig eller ansträngt dig kroppsligt i ditt arbete under det senaste året?**
Denna fråga besvaras också om du arbetar i hemmet.

☐ **jag har ålderspension/förtidspension**

☐ **stillasittande arbete**
Du har övervägande stillasittande arbete och går inte mycket ut under tiden .T.ex. skrivbordsarbete, urmakeri och montering av lättare delar.

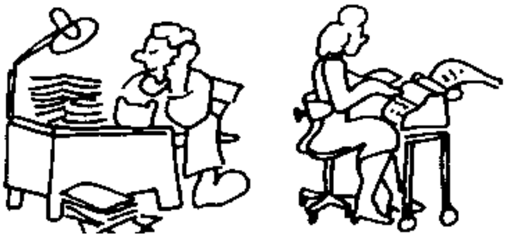

☐ **lätt, men något rörligt arbete.**
Du har ett arbete där du går ganska mycket men inte bär eller lyfter tyngre saker. T.ex. rörligt expeditionsarbete, lätt industriarbete, förmanssysslor, sådan undervisning där man går mycket, affärsbiträde, hushållsarbete

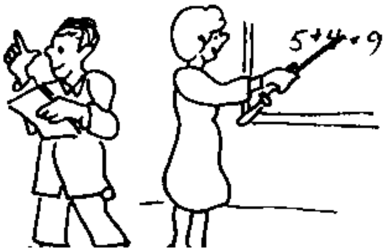

☐ **måttligt tungt arbete.**
Du går mycket och lyfter dessutom ganska mycket eller går uppför trappor eller backar. T.ex. brevbärare, arbete vid tyngre industri, byggnadssnickeri, rörläggningsarbete, vårdarbete

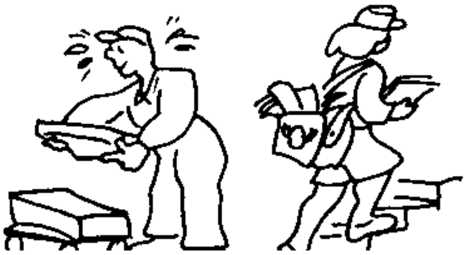

☐ **tungt arbete.**
Du har ett tungt kroppsarbete, lyfter tunga föremål och anstränger dig mycket kroppsligt. T.ex. skogsarbete, tungt lantbruksarbete, fiske med tunga redskap, byggnadsarbete

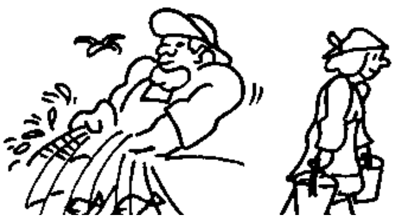

121. **Hur lång är du?**

[ ] centimeter

122. **Hur mycket väger du?**

[ ] kg

Ifylles endast av kvinnor
Männen kan sluta att fylla i formuläret här.

123. Hur många gånger har du varit gravid

[ ] gånger

124. Hur många barn har du fött?

[ ] barn

125. Om du fött barn, hade du då graviditetsdiabetes (dvs. socker i urinen eller förhöjt blodsocker) under graviditeten?

- ☐ ja
- ☐ nej
- ☐ har inga barn
- ☐ vet ej

Om **ja** ange födelsevikt på barnet/barnen

[ ] g	[ ] g	[ ] g
[ ] g	[ ] g	[ ] g

126. Har du fortfarande menstruation varje månad?

☐ ja, regelbundet varje månad	➔ gå till fråga **127**
☐ ibland, men inte så regelbundet som tidigare, eller att menstruationen har upphört de senaste sex månaderna	➔ gå till fråga **127**
☐ nej, det är mer än sex månader sedan den upphörde	➔ gå till fråga **131**
☐ jag är gravid	➔ gå till fråga **127**

127. Om du menstruerar regelbundet/ibland, försök ange <u>senaste menstruationens</u> <u>första dag.</u>

(Ange år, månad, dag) [ ]

128. Har du tagit preventivmedel (p-piller) eller preventivinjektion de senaste två månaderna?

- ☐ ja
- ☐ nej

129. Vilket/vilka preventivmedel har du använt under senaste året?

- ☐ använder inte preventivmedel
- ☐ p-piller
- ☐ minipiller
- ☐ hormonspiral
- ☐ p-stavar
- ☐ p-spruta
- ☐ annan metod

130. Har du någon gång under de senaste 2 åren vid något tillfälle haft mer än 35 dagar mellan menstruationerna?

- ☐ ja
- ☐ nej

131. Har du ökad behåring på kroppen i jämförelse med jämnåriga kvinnor?

- ☐ ja
- ☐ nej

Om du menstruerar eller är gravid så kan du sluta fylla i enkäten här. Övriga kvinnor går till nästa fråga.

I fylles endast av kvinnor som är i eller har passerat klimakteriet.

132. Hur gammal var du när menstruationerna upphörde helt?

☐ år

133. Har du använt något östrogenpreparat (ex tabletter eller plåster) för dina klimakteriebesvär under den senaste månaden?

- ☐ ja
- ☐ nej, men har tidigare använt
- ☐ nej

134. Om du använder eller har använt östrogenpreparat, ange antal år:

- ☐ 0-1 år
- ☐ 2-5 år
- ☐ 6-10 år
- ☐ mer än 10 år

TACK FÖR DIN MEDVERKAN!

Var vänlig tag med detta formulär när du går till undersökningen
