# Supplementary material for: Time trends of comparative self-rated health in adults aged 25-34 in the Northern Sweden MONICA study, 1990-2014
Source: PLoS One. 2017 Nov 20;12(11):e0187896. doi: 10.1371/journal.pone.0187896 (PMC5695772; doi:10.1371/journal.pone.0187896)
Supplement: S6 Text — (PDF) [file pone.0187896.s006.pdf]

NORRA SVERIGE
MONICA
HÄLSOUNDERSÖKNING

Frågeformulär

Norra Sveriges MONICA-undersökning 2014

en befolkningsstudie avseende riskfaktorer
för hjärt-kärlsjukdom och diabetes

VÄSTERBOTTENS LÄNS LANDSTING

NORRBOTTENS LÄNS LANDSTING

INFORMATION

Det frågeformulär som vi nu ber dig att besvara, innehåller frågor rörande bl.a. hälsa, arbete, fysisk aktivitet, rök-, kost- och motionsvanor.
Alla uppgifter som du lämnar kommer att behandlas konfidentiellt. Resultaten kommer att sammanställas på sådant sätt att det inte går att identifiera enskilda individer.
Om det är något du funderar över angående frågeformulären eller undersökningen, kontakta gärna någon av oss. Du når oss säkrast på telefon på förmiddagarna.
Om vi skulle få problem med att tolka något av dina ifyllda svar, skulle vi vara mycket tacksamma om vi får ringa dig eller skicka e-post för att reda ut det som är oklart. Därför är det bra om du anger det telefonnummer där vi lättast kan nå dig (helst dagtid) och eventuell e-post-adress.

Så här fyller du i enkäten

Att besvara enkäten tar ungefär en halv timme. Enkätsvaren registreras maskinellt, därför är det viktigt att de är ifyllda på rätt sätt.

- Använd en bra penna med **svart eller mörkblå färg**. Undvik blyertspenna.
- Sätt ett kryss i de rutor du tycker stämmer bäst för dig. Markera om möjligt inom rutorna.

Så här: ☒

- Om du råkar kryssa fel fyller du i hela den rutan med färg.

Så här: ■

Då kan inte maskinen läsa den. Kryssa sedan i den rätta rutan.

Vi ber dig att noga läsa igenom frågorna och svarsalternativen, innan du besvarar frågorna.

Mitt telefonnummer dagtid är: ____________________

Jag träffas säkrast:

Efter klockan	Fram till klockan

E-postadress: ____________________

Gunborg Rönnberg
Forskningssjuksköterska
MONICA-projektet
Medicincentrum
Norrlands Universitetssjukhus
901 85 UMEÅ
Tel: 090 - 785 96 01

Karin Ruikka
Forskningssjuksköterska
MONICA-projektet
Björkskatans hälsocentral
Höstvägen 7
976 27 LULEÅ
Tel: 0920 – 719 47
Epost: karin.ruikka@nll.se

Endast ett X på varje fråga där inte annat anges

______ Frågor rörande CIVILSTÅND och BOENDE ________

1. Vilket är ditt nuvarande civilstånd?

- ☐ ogift
- ☐ gift, registrerat partnerskap eller sammanboende
- ☐ skild eller separerad
- ☐ änka/änkling
- ☐ annat____

2. Vilka personer sammanbor du med?

- ☐ vuxen (make, maka, sambo, partner)
- ☐ barn, *ange antal barn* ☐
- ☐ vuxen och barn, *ange antal barn* ☐
- ☐ annan
- ☐ bor ensam

3. Var bor du?

- ☐ större tätort - Umeå, Skellefteå, Luleå, Boden, Piteå, Kiruna, orter med än 15 000 invånare
- ☐ annan tätort med mer än 1 000 invånare
- ☐ ort med mindre än 1 000 invånare

4. I vilket land är du född?

- ☐ Sverige ➔ gå till fråga **5**
- ☐ Finland ➔ gå till fråga **6**
- ☐ annat Skandinaviskt land ➔ gå till fråga **6**
- ☐ övriga Europa ➔ gå till fråga **6**
- ☐ annat utomeuropeiskt land ➔ gå till fråga **6**

5. Var är du född? *(Ange vilken kyrkoförsamling, ort och län du tillhörde vid födseln)*

Församling	Ort	Län

Frågor rörande LEVNADSVANOR

6. **Röker du cigaretter för närvarande?**

- ☐ ja, regelbundet (1 cigarett eller mer per dag)
- ☐ nej ➔ gå till fråga **10**
- ☐ ibland (mindre än 1 cigarett per dag) ➔ gå till fråga **10**

7. **Ungefär hur många cigaretter röker du i genomsnitt per dag?**

ange antal cigaretter ☐ */dag*

8. **Hur många dagar i veckan röker du cigaretter?**

- ☐ vanligtvis en dag eller mindre
- ☐ vanligtvis 2 till 4 dagar
- ☐ nästan varje dag

9. **Ungefär hur många cigaretter röker du i genomsnitt per vecka?**

ange antal cigaretter ☐ */vecka*

10. **Har du någonsin rökt cigaretter regelbundet tidigare?**

- ☐ ja, regelbundet (1 cigarett eller mer per dag)
- ☐ nej ➔ gå till fråga **15**

11. **När slutade du att röka cigaretter regelbundet?**

ange årtal, 4 siffror ☐

12. **Om du slutade röka under det senaste året, när slutade du?**

- ☐ för mindre än en månad sedan
- ☐ för 1-6 månader sedan
- ☐ för 7-12 månader sedan

13. **Vilket är det högsta antal cigaretter/dag, som du rökt under en så lång period som ett år?**

ange antal cigaretter ☐ */dag*

14. **Hur gammal var du när du började röka cigaretter?**

ange ålder ☐

15. **Hur du någonsin rökt pipa?**

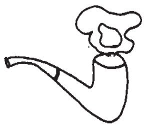

- ☐ ja, för närvarande regelbundet
- ☐ nej ➔ gå till fråga **17**
- ☐ jag röker pipa ibland (mindre än 1 gång/dag)
- ☐ tidigare, men inte nu ➔ gå till fråga **17**

16. **Hur många gram tobak röker du per vecka?**

Ett paket piptobak väger som regel 50 gram *ange antal gram* ☐ */vecka*

17. **Har du någonsin använt snus?**

- ☐ ja, snusar mindre än 2 dosor per vecka
- ☐ ja, snusar 2 - 4 dosor per vecka
- ☐ ja, snusar mer än 4 men mindre än 7 dosor per vecka
- ☐ ja, snusar 7 dosor eller fler per vecka
- ☐ ja, snusade tidigare men inte nu
- ☐ nej ➔ gå till fråga **21**

18. **Hur många år har du använt snus?**

ange antal år ☐

19. **Började du att snusa i samband med att du slutade röka?**

- ☐ ja
- ☐ nej
- ☐ jag både röker och snusar

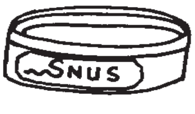

20. **Vilken typ av snus har du använt som mest?**

- ☐ Lössnus
- ☐ Portionsnus
- ☐ Miniportionsnus

21. **Hur ofta dricker du alkohol (gäller senaste året)?**

- ☐ aldrig ➔ gå till fråga **24**
- ☐ 1 gång i månaden eller mer sällan
- ☐ 2-4 ggr i månaden
- ☐ 2-3 ggr i veckan
- ☐ 4 ggr i veckan eller mer

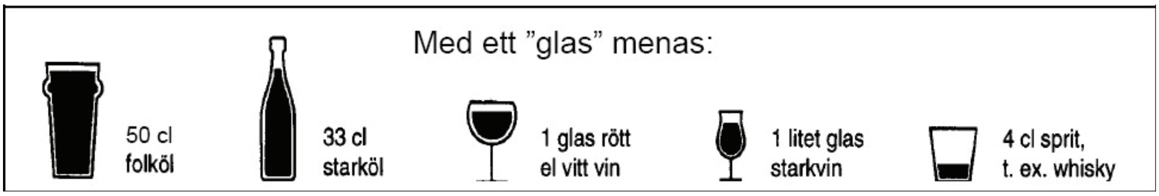

22. **Hur många glas (se bild ovan) dricker du en typisk dag då du dricker alkohol?**

- ☐ 1-2
- ☐ 3-4
- ☐ 5-6
- ☐ 7-9
- ☐ 10 eller fler

23. **Hur ofta dricker du sex sådana ”glas” eller mer vid samma tillfälle?**

- ☐ aldrig
- ☐ mer sällan än en gång i månaden
- ☐ varje månad
- ☐ varje vecka
- ☐ dagligen eller nästan varje dag

Frågor rörande HÄLSA

Har du av läkare fått diagnos och behandlats (medicinskt eller kirurgiskt) för någon av följande sjukdomar?
Om du vårdats för samma sjukdom flera gånger, ange senaste vårdtillfället!

24. **Hjärtinfarkt /propp i hjärtat?**

- ☐ ja, *ange årtal, 4 siffror* ☐
- ☐ Ja, flera *ange senaste årtal, 4 siffror* ☐
- ☐ nej
- ☐ vet ej

Fortsättning: Har du av läkare fått diagnos och behandlats (medicinskt eller kirurgiskt) för någon av följande sjukdomar? *Om du vårdats för samma sjukdom flera gånger, ange årtal,* ***4 siffror****, för* *<u>senaste</u>* *vårdtillfälle/vårdkontakt!*

25. Kärlkramp/anginapectoris?

- ☐ ja, *ange årtal* ☐
- ☐ nej
- ☐ vet ej

26. Opererat hjärtats kranskärl med by-pass eller ballongvidgning?

- ☐ ja, *ange årtal* ☐
- ☐ nej
- ☐ vet ej

27. Förmaksflimmer?

- ☐ ja, *ange årtal* ☐
- ☐ nej
- ☐ vet ej

28. Hjärtsvikt?

- ☐ ja, *ange årtal* ☐
- ☐ nej
- ☐ vet ej

29. Hjärtklaffsjukdom?

- ☐ ja
- ☐ nej
- ☐ vet ej

om ja och **opererad**, *ange årtal* ☐

Ange vilken typ av klaff

- ☐ aortaklaff
- ☐ mitralisklaff
- ☐ annan klaff
- ☐ flera klaffar
- ☐ vet ej

Fortsättning: Har du av läkare fått diagnos och behandlats (medicinskt eller kirurgiskt) för någon av följande sjukdomar? *Om du vårdats för samma sjukdom flera gånger, ange årtal, **4 siffror**, för* <u>*senaste*</u> *vårdtillfälle/vårdkontakt!*

30. Åtgärdat förträngningar i pulsåderkärl, t.ex ben eller halskärl?

☐ ja, *ange årtal* ☐

☐ nej

☐ vet ej

31. Åtgärdat vidgning av stora kroppspulsådern (aorta)?

☐ ja, *ange årtal* ☐

☐ nej

☐ vet ej

32. Stroke/slaganfall (hjärnblödning eller blodpropp i hjärnan)?

☐ ja, *ange årtal* ☐

☐ nej

☐ vet ej

33. Högt blodtryck?

☐ ja, *ange årtal* ☐ Hälsocentral ____________________

☐ nej

☐ vet ej

34. Höga blodfetter/högt kolesterol?

☐ ja, *ange årtal* ☐ Hälsocentral ____________________

☐ nej

☐ vet ej

35. Diabetes?

☐ ja, *ange årtal* ☐ Hälsocentral ____________________

☐ nej ➔ gå till fråga **38**

☐ vet ej ➔ gå till fråga **38**

36. **Hur behandlar du din diabetes och vilket år började du behandlingen?**

- ☐ insulin — *ange årtal, 4 siffror* ☐
- ☐ tabletter — *ange årtal, 4 siffror* ☐
- ☐ både insulin och tabletter — *ange årtal, 4 siffror* ☐
- ☐ enbart kost — *ange årtal, 4 siffror* ☐

37. **Hur gammal var du när du fick diabetes?**

ange <u>ålder</u> ☐

Fortsättning: Har du av läkare fått diagnos och behandlats (medicinskt eller kirurgiskt) för någon av följande sjukdomar? *Om du vårdats för samma sjukdom flera gånger, ange årtal, **4 siffror**, för <u>senaste</u> vårdtillfälle/vårdkontakt!*

38. **Kronisk obstruktiv lungsjukdom (KOL), kronisk bronkit eller emfysem?**

- ☐ ja, — *ange årtal* ☐
- ☐ nej
- ☐ vet ej

39. **Sömnapné/andningsuppehåll under sömn?**

- ☐ ja, — *ange årtal* ☐
- ☐ nej
- ☐ vet ej

40. **Reumatisk sjukdom, reumatoid artrit/Bechterews sjukdom/ psoriasisartrit /SLE/ Sjögrens syndrom?**

- ☐ ja, — *ange årtal* ☐
- ☐ nej
- ☐ vet ej

41. **Har du under de senaste <u>14 dagarna</u> använt något läkemedel som ordinerats av läkare?**

- ☐ ja
- ☐ nej ➔ gå till fråga **43**

42. **Om du svarat "ja" i så fall vilken eller vilka mediciner?**

1 ____________ 4 ____________

2 ____________ 5 ____________

3 ____________ 6 ____________

43. **Har läkare eller annan sjukvårdspersonal föreskrivet en särskild diet/kost till dig, för att sänka dina kolesterol/blodfettnivåer?**

- ☐ ja
- ☐ nej
- ☐ håller en föreskriven diet, men **vet ej** om den är till för att sänka mina blodfettnivåer

44. **Har du fått ditt kolesterol/blodfett mätt under det senaste året?**

- ☐ ja
- ☐ nej
- ☐ vet ej

45. **Får du smärtor - stickningar - ont i bröstet när du går uppför backar eller trappor, eller när du går fort på plan mark?**

- ☐ ja
- ☐ nej

46. **Får du smärtor - stickningar - ont i bröstet när du går i vanlig takt på plan mark?**

- ☐ ja
- ☐ nej

Om du svarat **nej** på både fråga 45 och 46, gå till fråga **50**

47. **Om du får smärtor eller obehag i bröstet i samband med att du rör dig, vad brukar du då göra?**

- ☐ stanna
- ☐ sakta ner farten
- ☐ fortsätta i samma takt

48. **Om du stannar eller saktar ner, försvinner smärtorna då?**

- ☐ ja
- ☐ nej ➔ gå till fråga **50**

49. **Om de försvinner, hur snart försvinner de?**

- ☐ efter mindre än 10 minuter
- ☐ efter mer än 10 minuter

50. Har du någon gång haft svår smärta i bröstet som varat i en halvtimme eller mer?

- ☐ ja
- ☐ nej

51. Brukar du få smärtor i vaderna, när du går i uppförsbackar, trappor eller på plan mark?

- ☐ ja
- ☐ nej

52. Blir du andfådd av att gå två trappor upp eller motsvarande i samma takt som jämnåriga?

- ☐ ja
- ☐ nej

53. Har du haft pip eller har det väst i bröstet vid något tillfälle under de senaste 12 månaderna?

- ☐ ja
- ☐ nej ➔ gå till fråga **56**

54. Har du överhuvudtaget varit det minsta andfådd när du haft detta pip eller väsande ljud i bröstet?

- ☐ ja
- ☐ nej

55. Har du haft detta pip eller väsande i bröstet när du <u>inte</u> samtidigt varit förkyld?

- ☐ ja
- ☐ nej

56. Hostar eller harklar du upp slem (eller har slem som det är svårt att få upp, trots hosta) de flesta dagar i perioder om minst 3 månader per år?

- ☐ ja, *ange hur många år* ☐
- ☐ nej

57. Vaknar du nattetid på grund av andfåddhet?

- ☐ ja, varje natt
- ☐ ja, en gång per vecka eller mer
- ☐ ja, mindre än en gång per vecka (1-3 ggr/mån)
- ☐ nej

58. Har du någon gång av läkare fått diagnosen sorkfeber?

- ☐ ja *ange årtal, 4 siffror* ☐
- ☐ nej

59. Vet du eller tror du, att du haft harpest?

- ☐ ja, jag vet det *ange årtal, 4 siffror* ☐
- ☐ ja, jag tror det *ange årtal, 4 siffror* ☐
- ☐ nej

60. Har du haft någon kontakt med katt under tidsperioden augusti till och med oktober 2013?

- ☐ ja
- ☐ nej

61. Vilken boendeform hade du under tidsperioden augusti till och med oktober 2013?
Fler alternativ är möjliga!

- ☐ hus/villa i tätort ☐ antal veckor under perioden
- ☐ lägenhet ☐ antal veckor under perioden
- ☐ sommarbostad ☐ antal veckor under perioden
- ☐ lantbruk/hus/villa i glesbygd ☐ antal veckor under perioden

62. Uppskattningsvis, hur många timmar per vecka tidsperioden augusti till och med oktober 2013 har du i genomsnitt vistats utomhus?

ange antal timmar ☐

63. Uppskattningsvis, hur många timmar per vecka tidsperioden augusti till och med oktober 2013 har du i genomsnitt vistats i skog och mark? *Ex bär/svampplockning, jakt, motion*

ange antal timmar ☐

64. Har du någon gång av läkare fått diagnosen Ockelbosjukan?

- ☐ ja, jag vet det *ange årtal, 4 siffror* ☐
- ☐ ja, jag tror det *ange årtal, 4 siffror* ☐
- ☐ nej

65. Hur ofta äter du älgfärs eller grytbitar från älg?

☐ aldrig
☐ någon gång per år
☐ 1-3 ggr per månad
☐ 1 gång per vecka
☐ 2-3 ggr i veckan
☐ 4-6 ggr i veckan

66. Hur ofta äter du mat där råvaror kommer från konservburkar?

☐ varje dag
☐ varannan dag
☐ några dagar per vecka
☐ 1 gång per vecka
☐ mer sällan

Frågor om sjukdom i SLÄKTEN

67. Finns det någon i din familj som insjuknat/avlidit i hjärtinfarkt ?
Här avses biologiska föräldrar och syskon! Flera alternativ är möjliga!

☐ ja, min mamma
☐ ja, min pappa
☐ ja, något/några syskon
☐ nej
☐ vet ej

68. Finns det någon i din familj som insjuknat/avlidit i stroke/slaganfall?
Här avses biologiska föräldrar och syskon! Flera alternativ är möjliga!

☐ ja, min mamma
☐ ja, min pappa
☐ ja, något/några syskon
☐ nej
☐ vet ej

69. Finns det någon i din familj som har/har haft diabetes?
Här avses biologiska föräldrar, syskon och barn! Flera alternativ är möjliga!

- ☐ ja, min mamma
- ☐ ja, min pappa
- ☐ ja, något/några syskon
- ☐ ja, något/några barn
- ☐ nej
- ☐ vet ej

70. Finns det någon i din familj som har insjuknat i kronisk luftrörskatarr (bronkit) kronisk obstruktiv lungsjukdom (KOL) eller emfysem?
Här avses biologiska föräldrar, syskon och barn! Flera alternativ är möjliga!

- ☐ ja, min mamma
- ☐ ja, min pappa
- ☐ ja, något/några syskon
- ☐ ja, något/några barn
- ☐ nej
- ☐ vet ej

Frågor om ARBETE

71. Vilken är den högsta utbildning som du har avslutat?

- ☐ ej avslutat grundskola eller annan grundläggande utbildning
- ☐ grundskola, folkskola, yrkesutbildning eller motsvarande (högst 9 år)
- ☐ läroverk, gymnasium, folkhögskola eller motsvarande
- ☐ universitet eller högskola

72. Vilken är din nuvarande sysselsättning? *Du kan ange flera alternativ*.

- ☐ yrkesarbetar ange ☐ % av heltid
- ☐ tjänstledig eller föräldraledig
- ☐ studerar, praktiserar
- ☐ arbetsmarknadsåtgärd
- ☐ arbetslös
- ☐ ålderspensionär
- ☐ avtalspensionär
- ☐ förtidspensionär, sjukpensionär
- ☐ långtidssjukskriven (mer än 3 månader)
- ☐ övrigt – icke förvärvsarbetare

73. Vilket har varit ditt yrke/din sysselsättning under huvuddelen av ditt yrkesliv?

____

74. Har du yrkesarbetat de senaste 12 månaderna?

☐ ja

Om ja ange a: nuvarande yrke

____

och b: ange vilket år började du arbeta i detta yrke, 4 siffror ☐

☐ nej

75. Om du plötsligt skulle hamna i en situation, där du på en vecka måste skaffa fram 20 000 kronor, skulle du klara det?

☐ ja

☐ nej

76. Har det under de senaste 12 månaderna hänt att du haft svårigheter att klara utgifterna för mat, hyra, räkningar etc.?

☐ ja

☐ nej

Männen går vidare till fråga 80.

77. Har du fortfarande menstruation varje månad?

☐ ja, regelbundet varje månad

☐ ibland, men inte så regelbundet som tidigare, eller att menstruationen har upphört de senaste sex månaderna

☐ nej, det är mer än sex månader sedan den upphörde

☐ jag är gravid

78. Har du under graviditet någon gång fått diagnosen graviditetsdiabetes?

☐ ja

☐ nej

☐ har inte varit gravid

79. Hur gammal var du när menstruationerna upphörde helt?

ange ålder ☐

Frågor rörande LIVSKVALITET

Besvara följande frågor genom att ange det svarsalternativ som bäst stämmer överens med din personliga situation/uppfattning

80. Hur tycker du ditt allmänna hälsotillstånd varit det senaste året?

- ☐ mycket gott
- ☐ ganska gott
- ☐ någorlunda
- ☐ tämligen dåligt
- ☐ dåligt

81. Hur bedömer du ditt allmänna hälsotillstånd jämfört med andra personer i din egen ålder?

- ☐ det är bättre
- ☐ det är sämre
- ☐ ungefär likadant

82. Jämfört med andra personer av samma ålder och kön som du själv, känner du dig ”yngre” eller ”äldre” än dessa rent kroppsligen?

- ☐ ja, jag känner mig kroppsligen ”yngre”
- ☐ jag känner mig kroppsligen i samma ålder som jämnåriga
- ☐ ja, jag känner mig kroppsligen ”äldre”
- ☐ vet inte

83. Brukar andra personer uppfatta att du förefaller "yngre” eller "äldre" än jämnåriga av samma kön?

- ☐ ja, jag uppfattas "yngre"
- ☐ jag uppfattas som jämnårig
- ☐ ja, jag uppfattas "äldre"
- ☐ vet inte

Markera, genom att kryssa <u>*en ruta i varje grupp*</u>, vilket påstående som bäst beskriver ditt hälsotillstånd och eventuellt hjälpbehov i dag

84. Rörlighet

- ☐ jag går utan svårighet
- ☐ jag kan gå men med viss svårighet
- ☐ jag är sängliggande

85. **Hygien**

- ☐ jag behöver ingen hjälp med min dagliga hygien eller påklädning
- ☐ jag har vissa problem med att tvätta och klä mig
- ☐ jag kan inte tvätta eller klä mig själv

86. **Huvudsakliga aktiviteter** (t ex arbete, studier, hushållsysslor, familje- och fritidsaktiviteter)

- ☐ jag klarar av mina huvudsakliga aktiviteter
- ☐ jag har vissa problem med att klara av mina huvudsakliga aktiviteter
- ☐ jag klarar inte av mina huvudsakliga aktiviteter

87. **Smärtor/besvär**

- ☐ jag har varken smärtor eller besvär
- ☐ jag har måttliga smärtor eller besvär
- ☐ jag har svåra smärtor eller besvär

88. **Oro/nedstämdhet**

- ☐ jag är inte orolig eller nedstämd
- ☐ jag är orolig eller nedstämd i viss utsträckning
- ☐ jag är i högsta grad orolig eller nedstämd

Genom följande påståenden vill vi få en uppfattning om hur du känt dig **de senaste dagarna**. Som ett exempel, tänk på påståendet ”jag har känt mig avspänd”. Om du tycker att det stämmer fullständigt med hur du känt dig, sätt då ett kryss längst till vänster, så här:

EXEMPEL: jag har känt mig avspänd

Ja, det stämmer helt	☒ 1	☐ 2	☐ 3	☐ 4	☐ 5	Nej, det stämmer inte alls

Ju mindre du instämmer med påståendet, desto längre till höger placerar du krysset. Var vänlig och ta ställning till samtliga påståenden.

89. **Jag känner mig i form**

Ja, det stämmer helt	☐ 1	☐ 2	☐ 3	☐ 4	☐ 5	Nej, det stämmer inte alls

90. **Kroppsligt känner jag mig bara i stånd att göra väldigt lite**

Ja, det stämmer helt ☐ 1 ☐ 2 ☐ 3 ☐ 4 ☐ 5 Nej, det stämmer inte alls

91. **Jag känner mig mycket aktiv**

Ja, det stämmer helt ☐ 1 ☐ 2 ☐ 3 ☐ 4 ☐ 5 Nej, det stämmer inte alls

92. **Jag har lust att göra en massa trevliga saker**

Ja, det stämmer helt ☐ 1 ☐ 2 ☐ 3 ☐ 4 ☐ 5 Nej, det stämmer inte alls

93. **Jag känner mig trött**

Ja, det stämmer helt ☐ 1 ☐ 2 ☐ 3 ☐ 4 ☐ 5 Nej, det stämmer inte alls

94. **Jag tycker att jag hinner med mycket på en dag**

Ja, det stämmer helt ☐ 1 ☐ 2 ☐ 3 ☐ 4 ☐ 5 Nej, det stämmer inte alls

95. **När jag gör något kan jag koncentrera mig på det**

Ja, det stämmer helt ☐ 1 ☐ 2 ☐ 3 ☐ 4 ☐ 5 Nej, det stämmer inte alls

96. **Kroppsligt orkar jag mycket**

Ja, det stämmer helt ☐ 1 ☐ 2 ☐ 3 ☐ 4 ☐ 5 Nej, det stämmer inte alls

97. **Jag fasar för att behöva göra något**

Ja, det stämmer helt ☐ 1 ☐ 2 ☐ 3 ☐ 4 ☐ 5 Nej, det stämmer inte alls

98. **Jag får väldigt lite gjort under en dag**

Ja, det stämmer helt ☐ 1 ☐ 2 ☐ 3 ☐ 4 ☐ 5 Nej, det stämmer inte alls

99. **Jag har lätt för att koncentrera mig**

Ja, det stämmer helt ☐ 1 ☐ 2 ☐ 3 ☐ 4 ☐ 5 Nej, det stämmer inte alls

100. Jag är utvilad

Ja, det stämmer helt ☐ 1 ☐ 2 ☐ 3 ☐ 4 ☐ 5 Nej, det stämmer inte alls

101. Jag använder mycket kraft för att koncentrera mig på saker

Ja, det stämmer helt ☐ 1 ☐ 2 ☐ 3 ☐ 4 ☐ 5 Nej, det stämmer inte alls

102. Kroppsligt känner jag mig i dålig form

Ja, det stämmer helt ☐ 1 ☐ 2 ☐ 3 ☐ 4 ☐ 5 Nej, det stämmer inte alls

103. Jag har massor av planer

Ja, det stämmer helt ☐ 1 ☐ 2 ☐ 3 ☐ 4 ☐ 5 Nej, det stämmer inte alls

104. Jag blir lätt trött

Ja, det stämmer helt ☐ 1 ☐ 2 ☐ 3 ☐ 4 ☐ 5 Nej, det stämmer inte alls

105. Jag får inte mycket gjort

Ja, det stämmer helt ☐ 1 ☐ 2 ☐ 3 ☐ 4 ☐ 5 Nej, det stämmer inte alls

106. Jag har ingen lust att göra något

Ja, det stämmer helt ☐ 1 ☐ 2 ☐ 3 ☐ 4 ☐ 5 Nej, det stämmer inte alls

107. Mina tankar far lätt iväg

Ja, det stämmer helt ☐ 1 ☐ 2 ☐ 3 ☐ 4 ☐ 5 Nej, det stämmer inte alls

108. Kroppsligt känner jag mig i utmärkt form

Ja, det stämmer helt ☐ 1 ☐ 2 ☐ 3 ☐ 4 ☐ 5 Nej, det stämmer inte alls

109. Vi vill att du på denna skala markerar hur bra eller dåligt ditt hälsotillstånd är, som du själv bedömer det. Den här skalan är numrerad från 0 till 100.

- 100 är den <u>bästa</u> hälsa du kan tänka dig.
  0 är den <u>sämsta</u> hälsa du kan tänka dig.

Skriv nu i rutan nedan det nummer på skalan mellan 0 till 100 som visar din hälsa IDAG.

DIN HÄLSA IDAG = ☐

Bästa hälsa du kan tänka dig

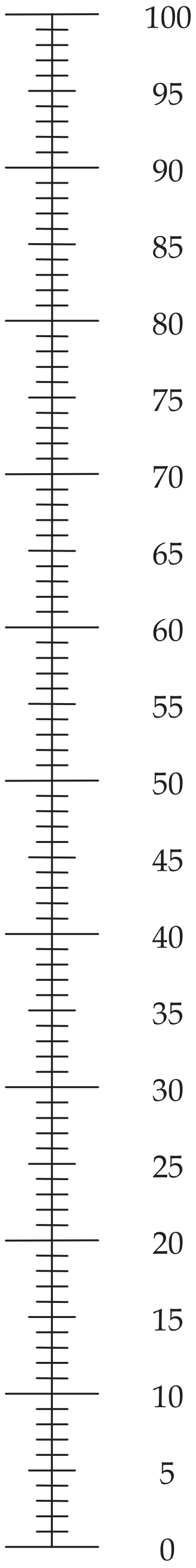

Sämsta hälsa du kan tänka dig

Frågor om FYSISK AKTIVITET

110. Markera i tabellen nedan hur du oftast färdas till och från arbetet varje årstid.
Du som inte arbetar kan gå till fråga ***114***

Flera alternativ möjliga, markera med kryss

	**Vår**	**Sommar**	**Höst**	**Vinter**
**Bil**	☐	☐	☐	☐
**Buss, tåg, båt**	☐	☐	☐	☐
**Promenerar**	☐	☐	☐	☐
**Cyklar**	☐	☐	☐	☐

111. Hur många kilometer har du till arbetet? *(enkel resa)*

ange antal km ☐

Sätt bara X i ett av alternativen

112. Markera det alternativ som bäst beskriver ditt arbete

- ☐ stillasittande eller stående
- ☐ lätt men delvis rörligt
- ☐ lätt och rörligt
- ☐ ibland fysiskt tungt
- ☐ fysiskt tungt större delen av tiden

113. Tänk på den tid som du tillbringat sittande på *arbetet* under en typisk arbetsdag den senaste arbetsveckan?

Hur mycket tid har du tillbringat sittande under en sådan dag?

ange timmar ☐ */dag*

ange minuter ☐ */dag*

114. Under en <u>vanlig</u> vecka det sista året, hur många timmar per vecka utförde du någon av följande aktiviteter?

Promenerade (till arbete, till affär och på fritid etc)

sommartid *ange timmar* ☐ */vecka*

vintertid *ange timmar* ☐ */vecka*

Cyklade (till arbete, till affär och på fritid etc)

sommartid *ange timmar* ☐ */vecka*

vintertid *ange timmar* ☐ */vecka*

115. Hur ofta har du tränat eller motionerat i träningskläder de senaste tre månaderna, i syfte att förbättra din kondition och/eller för att må bra?

aldrig

då och då – ej regelbundet

1-2 ggr/vecka

2-3 ggr/vecka

mer än 3 ggr/vecka

116. Hur mycket har du rört dig och ansträngt dig kroppsligt på fritiden under de senaste 12 månaderna?

☐ **Stillasittande fritid**
(*Du ägnar dig mest åt läsning, TV, datorer eller annan stillasittande sysselsättning på fritiden. Du promenerar, cyklar eller rör dig på annat sätt mindre än 2 timmar i veckan.)*

☐ **Måttlig motion på fritiden**
(*Du promenerar, cyklar eller rör dig på annat sätt under <u>minst 4 timmar</u> i veckan. I detta inräknas t.ex. promenad eller cykling till och från arbetet, övriga promenader, tyngre hushållsarbete, ordinärt trädgårdsarbete, fiske, bordtennis, bowling)*

☐ **Måttlig men regelbunden motion på fritiden**
(Du ägnar dig åt t ex löpning, simning, tennis, badminton, motionsgymnastik eller liknande. Tyngre trädgårdsarbete och liknande räknas till denna grupp. Observera att det ska vara i genomsnitt minst 2-3 timmar i veckan. Du motionerar regelbundet <u>1-2 ggr per vecka minst 30 minuter per gång</u> med t.ex. löpning, simning, tennis, badminton eller annan aktivitet <u>som gör att du svettas</u>.)

☐ **Regelbunden motion och träning**
(*Du ägnar dig åt t.ex. hård träning eller tävling i löpning, orientering, skidåkning, simning, simning, fotboll, handboll etc regelbundet och flera gånger i veckan. Tennis, badminton, motionsgymnastik eller liknande vid genomsnitt <u>minst 3 tillfällen</u> per vecka. Varje tillfälle varar <u>minst 30 minuter</u> per gång.)*

117. **Tänk på den tid som du tillbringat sittande under en _hel_ typisk dag, de senaste 7 dagarna?**
(I samband med arbete, studier, transporter, i hemmet och på din fritid, exempelvis tid vid skrivbordet, hemma hos vänner eller i TV-soffan.)

Hur mycket tid har du totalt tillbringat sittande under en sådan dag?

ange timmar ☐ *dag*

ange minuter ☐ *dag*

Frågor om SÖMN

118. **Har du de senaste 2 veckorna haft några svårigheter med att somna?**

- ☐ inga
- ☐ lindriga
- ☐ måttliga
- ☐ svåra
- ☐ mycket svåra

119. **Har du de senaste 2 veckorna haft problem med att få sova en hel natt?**

- ☐ inga
- ☐ lindriga
- ☐ måttliga
- ☐ svåra
- ☐ mycket svåra

120. **Har du de senaste 2 veckorna haft problem med att du vaknar för tidigt?**

- ☐ inga
- ☐ lindriga
- ☐ måttliga
- ☐ svåra
- ☐ mycket svåra

121. **Hur nöjd/missnöjd är du med ditt nuvarande sömnmönster?**

- ☐ mycket nöjd ➔ gå till fråga **125**
- ☐ nöjd ➔ gå till fråga **125**
- ☐ varken nöjd eller missnöjd
- ☐ missnöjd
- ☐ mycket missnöjd

122. **I vilken utsträckning anser du att dina sömnproblem stör ditt liv (ditt arbete, vid aktiviteter, koncentration, minne, humör o.s.v.)?**

- ☐ stör inte alls
- ☐ Lite
- ☐ Måttligt
- ☐ mycket
- ☐ väldigt mycket

123. **Hur märkbart för andra tror du ditt problem med nedsatt sömnkvalitet är?**

- ☐ inte alls märkbart
- ☐ lite
- ☐ måttligt
- ☐ mycket
- ☐ väldigt mycket

124. **Hur oroad eller besvärad är du över ditt sömnproblem?**

- ☐ inte alls oroad
- ☐ lite
- ☐ måttligt
- ☐ mycket
- ☐ väldigt mycket

125. **Hur mycket vätska dricker du per dygn?**

- ☐ mindre än 1,5 liter
- ☐ mellan 1,5 och 3 liter
- ☐ mer än 3 liter

126. **Vaknar du på natten av att du behöver kissa?**

- ☐ ja
- ☐ nej ➔ Gå till fråga **133**

127. **Om ja, hur ofta?**

- ☐ 1 gång/natt
- ☐ 2 gånger/natt
- ☐ 3 gånger/natt
- ☐ 4 gånger/natt
- ☐ 5 gånger eller mer/natt

128. **Hur ofta de senaste 4 veckorna har dina nattliga problem med att kissa gjort att du tycker att något är fel med dig?**

- ☐ aldrig
- ☐ sällan
- ☐ ibland
- ☐ ofta
- ☐ nästan alltid

129. **Hur ofta har det gjort dig frustrerad?**

- ☐ aldrig
- ☐ sällan
- ☐ ibland
- ☐ ofta
- ☐ nästan alltid

130. **Hur mycket har dina besvär förhindrat att du får den sömn du behöver?**

- ☐ aldrig
- ☐ sällan
- ☐ ibland
- ☐ ofta
- ☐ alltid

131. **Hur mycket har dina problem med att kissa påverkat ditt arbete om du varit yrkesverksam under de senaste 4 veckorna?** *Du som inte arbetar gå till fråga* ***132***

Tänk på dagar då du var begränsad av hur mycket du kunde arbeta, dagar då du uppnådde mindre än du skulle vilja göra och dagar då du inte kunde göra ditt arbete så noggrant som vanligt.

inte alls hindrat mej	☐	☐	☐	☐	☐	☐	☐	fullständigt hindrat mej
	**1**	**2**	**3**	**4**	**5**	**6**	**7**	

132. **Hur mycket har dina problem med att kissa påverkat din förmåga att utföra vanliga dagliga aktiviteter som inte är relaterade till ditt arbete de senaste 4 veckorna?**

Med vanliga aktiviteter menar vi aktiviteter du brukar göra som jobba kring huset, handla, ta hand om barn, träna, studera osv. Tänk på de gånger då du var begränsad i dina aktiviteter och dagar då du gjorde mindre än du skulle vilja göra.

inte alls hindrat mej	☐	☐	☐	☐	☐	☐	☐	fullständigt hindrat mej
	**1**	**2**	**3**	**4**	**5**	**6**	**7**	

Frågor om MILJÖPÅVERKAN

133. Vilken typ av golv har du i ditt sovrum?

- ☐ trägolv
- ☐ plastgolv
- ☐ heltäckningsmatta
- ☐ linoleum
- ☐ laminat
- ☐ sten/klinker
- ☐ inget av ovanstående

134. Hur gammalt är golvet i ditt sovrum?

- ☐ nyare än 5 år
- ☐ 5-15 år
- ☐ 16-30 år
- ☐ äldre än 30 år

Tack för din medverkan!
