# Supplementary material for: Time trends of comparative self-rated health in adults aged 25-34 in the Northern Sweden MONICA study, 1990-2014
Source: PLoS One. 2017 Nov 20;12(11):e0187896. doi: 10.1371/journal.pone.0187896 (PMC5695772; doi:10.1371/journal.pone.0187896)
Supplement: S7 Text — (DOCX) [file pone.0187896.s007.docx]

| 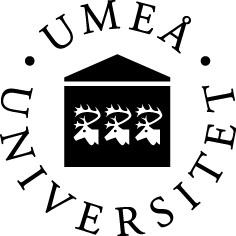 | Enheten för biobanksforskning  Umeå universitet |
| --- | --- |

# Variabelbeställning – VIP + MO

**Projektets titel:**

**Ansvarig forskare:**

**E-postadress:**

**Datauttaget ska tillvarata de vetenskapliga möjligheterna, men samtidigt begränsas till de variabler som är nödvändiga för projektets genomförande. Vänligen hjälp oss att dokumentera att så görs, genom att nedan kortfattat tala om varför dessa variabler behövs. Du behöver inte motivera varje enskild variabel, utan beskriv gärna i sammanfattande form.**

**Diarienummer och kortnamn (ifylles av EBF):**

**Antal registrerade enkäter**

***
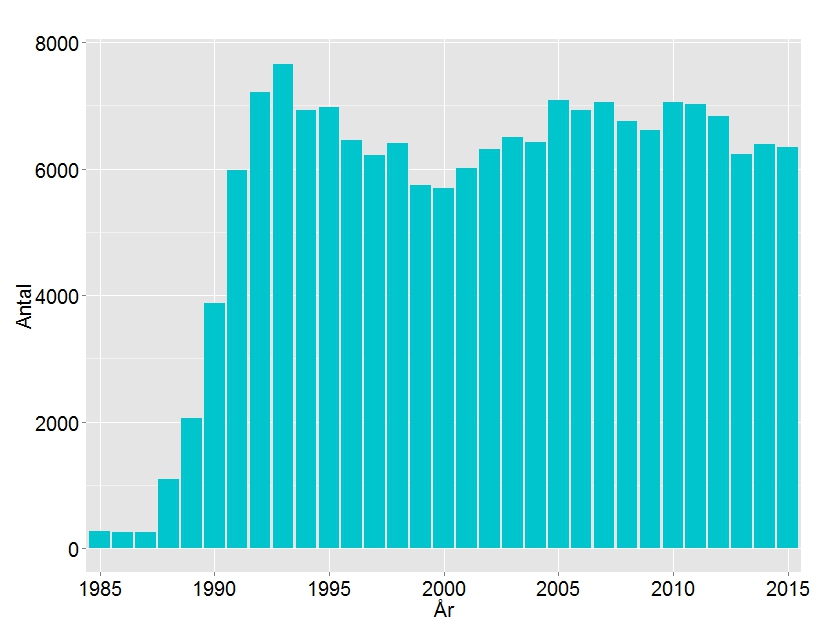
***

Diagrammet visar t.ex. att få enkäter inkom före 1990. För varje enkätvariabel nedan så är det också angivet när respektive fråga infördes på blanketten.

**Utlämning av data**

De data EBF lämnar ut är i regel harmoniserade data där enkäternas olikheter har vägts samman för både VIP och MONICA och där möjlighet finns att ansöka om bearbetade kostdata (som rensats för orealistiska värden och relaterats till bl.a. energiintag).

För att välja ut vilka variabler du har intresse av, fyll i formuläret nedan.

Om istället rådata önskas, kontakta EBF.

***Kryssa i önskat format***

| Önskat filformat vid levereras | Sas-format |
| --- | --- |
|  | Excel-format |
|  | Annat format, meddela i så fall detta i samband med att denna lista skickas in. (ebf@umu.se) |
|  | Vid leverans av data, önskar du variabellistan på:  Svenska  Engelska |

| Gruppering | Variabelnamn | Kategorier |
| --- | --- | --- |
| Bakgrundsvariabler Dessa variabler levereras alltid om de är aktuella för studien. | **id** | **För studien aktuellt id** |
|  | **case_control** | 1 = Fall  0 = Kontroll |
|  | **case_set** | **Fall/kontroll tillhörighet** |
|  | **date** | **Datum vid hälsoundersökning**  (år månad dag) |
|  | **gender** | **Kön**  1 = Man  2 = Kvinna |
|  | **delproj** | **Delkohort**  MO = MONICA-screening  VIP = Västerbotten intervention program |
|  | **diadat** | **Diagnosdatum**  (år månad dag) |
|  | **age** | **Ålder vid undersökningstillfället** |
|  | **fasta** | **Fastestatus** |
| ***Kryssa i önskade variabler i rutan framför variabelnamnet*** | | |
| Medicinska och antropometriska **variabler**  *I VIP provtas Hdl och Ldl för samtliga från 20090901. Värden finns innan dess på enstaka individer.  I MONICA finns värden på HDL och LDL 1986-1994. | **langd** | **Kroppslängd i cm** |
|  | **vikt** | **Vikt i kg** |
|  | **bmi** | **Body mass index** |
|  | **midja** | **Antal cm runt midjan** (VIP från 2003) |
|  | **skol** | **Totalkolesterol mmol/l** |
|  | **hdl** | **Hdl kolesterol mmol/l*** |
|  | **ldl** | **Ldl kolesterol mmol/l*** |
|  | **stg** | **Triglycerider mmol/l** |
|  | **blods0** | **Blodsocker 0-timmars värde** |
|  | **blods2** | **Blodsocker 2-timmars värde** |
|  | **sbt** | **Systoliskt blodtryck** (liggande värden fram till 20090831 och sittande värden från 20090901 gällande VIP. Sittande värden i delproj MA och MO). |
|  | **dbt** | **Diastoliskt blodtryck** (liggande värden fram till 20090831 och sittande värden från 20090901 gällande VIP. Sittande värden i delproj MA och MO). |
| Enkätvariabler | **civil**  VIP: 1985 ->  MONICA: 1986 - 2014 | **Civilstånd?** 1 = Ogift  2 = Gift/sambo + omgift/på nytt sambo  3 = Frånskild/separerad  4 = Änka/änkling |
|  | **utbild**  VIP: 1985 ->  MONICA: 1986 - 2014 | **Utbildningsnivå?** 1 = Folkskola + 9-årig grundskola (obligatorisk skola)  2 = Folkhögskola motsv. grundsk. + real + flickskola + yrkes-/fackskola  3 = Folkhögskola motsv. gymnasium + gymnasium/flickskola motsv. gymnasiekompetens  4 = Akademisk utbildning/högskola  5 = Har ej avslutat folkskola eller annan grundläggande utbildning (endast MONICA 2014)  6 = Grundskola, folkskola, yrkesutbildning eller motsvarande (högst 9 år)  (endast MONICA 2014) |
|  | **sambo**  VIP: 1985 ->  MONICA: 1986 – 2014 | **Vilka sammanbor du med?** 1 = Enbart en vuxen (make, maka, sambo)  2 = Enbart barn  3 = Vuxen och barn  4 = Annan/andra  5 = Bor helt ensam  6= Vuxna med eller utan barn (endast MONICA) |
|  | **skiftarbete**  VIP: 1985 ->  MONICA: 1986-2009 | **Har du skiftarbete/helgtjänstgöring?** 1 = Ja  2 = Nej |
|  | **sjukskriven****  VIP: 1989 ->  Gäller endast personer som deltagit i VIP | **Har du varit långtidssjukskriven mer än 6 månader?** 1 = Ja 2 = Nej |
|  | **arbsjukpens_8604**  MONICA: 1986-2004 Gäller endast personer som deltagit i MONICA | **Är just nu sjukskriven eller har ålders/förtids/sjukpens/bidrag?** 1 = Ja, pension/bidrag  2 = Ja, sjukskriven  3 = Nej |
|  | **arbsjukpens_0409**  MONICA: 2004-2009 Gäller endast personer som deltagit i MONICA | **Just nu sjukskriven, har ålders-, förtids-, sjukpension eller sjukbidrag?** 1 = Ja, ålders-/sjukpension på heltid  2 = Ja, ålders-/sjukpension på deltid 3 = Ja, förtidspension/sjukbidrag 4 = Ja, sjukskriven  5 = Nej |
|  | **arbsjukpens_14**  MONICA: 2014  Gäller endast personer som deltagit i MONICA | **Är du just nu något av följande?** 1 = Ja, ålderspensionär  2 = Ja, avtalspensionär  3 = Ja, förtidspensionär, sjukpensionär  4 = Ja, långtidssjukskriven  5 = Nej  Tvåsiffriga tal eller större anger vilka alternativ som kombinerats. |
|  | **arbyrke_8699**  MONICA: 1986-1999 Gäller endast personer som deltagit i MONICA | **Yrkesklassificering (NYK)**Vid intresse för denna variabel, vänligen kontakta Robert Lundqvist för närmare beskrivning. *Robert.Lundqvist*[*@nll.se*](mailto:per.ivarsson@medicin.umu.se) |
|  | **arbyrke_0409**  MONICA: 2004-2009 Gäller endast personer som deltagit i MONICA | **Yrkesklassificering (NYK)**Vid intresse för denna variabel, vänligen kontakta Robert Lundqvist för närmare beskrivning. *Robert.Lundqvist*[*@nll.se*](mailto:per.ivarsson@medicin.umu.se) |
|  | **ansttyp_a - ansttyp_m**  VIP: 1985 ->  MONICA: 1986-2014 | **Anställningstyp?** ansttyp_a = Fast anställning  ansttyp_b = Tillfällig anställning, vikariat, beredskapsarbete, arbetsmarknadsåtgärd  ansttyp_c = Arbetar i hemmet  ansttyp_d = Arbetslös  ansttyp_e = Studerar  ansttyp_f = Egen företagare  ansttyp_g = Förtids-/sjukpensionerad, helpension (**endast VIP**)  ansttyp_h = Förtids-/sjukpensionerad, delpension  ansttyp_i = Förtids-/sjukpensionerad, ospec. hel eller delpension (**endast VIP**)  ansttyp_j = Annat, övrigt (**endast MONICA**)  ansttyp_k = Tjänstledig eller föräldraledig (**endast MONICA 2014**)  ansttyp_l = Pensionär, ålderspensionär, avtalspensionär (**endast MONICA**)  ansttyp_m = Långtidssjukskriven (**endast MONICA 2014**) |
|  | **ursprungsland****  VIP: 1985 ->  MONICA: 1986-2014 | **Vilket land kommer du från?** 1 = Sverige  2 = Annat land, vilket? (se ursprungsland_vilket) |
|  | **ursprungsland_vilket****  VIP: 1985 ->  MONICA: 1986-2014 | **Anger vilket land personen kommer från** |
|  | **halsojf****  VIP: 1985 -2003  MONICA: 1990-2014 | **Allmänna hälsotillstånd jmf med andra i din ålder?** 1 = Bättre  2 = Ungefär likadant  3 = Sämre |
|  | **halsoal****  VIP: 1985-1994  MONICA: 1990-1994 | **Hur bedömer du ditt allmänna hälsotillstånd?** 1 = Gott  2 = Varken dåligt eller gott/något däremellan  3 = Dåligt |
|  | **halsoar****  VIP: 1989 ->  MONICA: 1986, 1999-2014 | **Hälsotillstånd senaste året?** 1 = Dåligt  2 = Tämligen dåligt  3 = Någorlunda  4 = Ganska gott  5 = Mycket gott |
|  | **hjartinf_foraldrar_syskon****  VIP: 1989 ->  Gäller endast personer som deltagit i VIP | **Har någon av dina föräldrar eller syskon före 60-års ålder drabbats av hjärtinfarkt eller hjärnblödning/propp i hjärnan?** 1 = Ja  2 = Nej  3 = Vet ej |
|  | **mislakt_9409**  MONICA: 1994-2009 Gäller endast personer som deltagit i MONICA | **Någon i släkten som avlidit i hjärtinfarkt före 65 års ålder?** 1 = Ja  2 = Nej  3 = Vet ej |
|  | **mifamilj_14**  MONICA: 2014 Gäller endast personer som deltagit i MONICA | **Någon i din familj som insjuknat/avlidit i hjärtinfarkt?** 1 = Ja  2 = Nej  3 = Vet ej |
|  | **cvsslakt_9409**  MONICA: 1994-2009 Gäller endast personer som deltagit i MONICA | **Finns det någon i din släkt som avlidit i slaganfall (propp eller blödning i hjärnan) före 65 års ålder?** 1 = Ja  2 = Nej 3 = Vet ej |
|  | **cvsfamilj_14**  MONICA: 2014 Gäller endast personer som deltagit i MONICA | **Någon i din familj som insjuknat/avlidit i stroke/slaganfall?** 1 = Ja  2 = Nej 3 = Vet ej |
|  | **diab_foraldrar_syskon****  VIP: 1989 ->  MONICA: 1999-2014 | **Förekommer diabetes hos någon av dina föräldrar och syskon?** 1 = Ja  2 = Nej  3 = Vet ej |
|  | **beskbltr****  VIP: 1988 ->  MONICA: 1986-2014 | **Har du vid något tillfälle fått besked om att du har högt blodtryck?**  1 = Ja  2 = Nej |
|  | **mediciner**  VIP: 1985 ->  MONICA: 1986-2014 | **Har du under den senaste 14 dagars perioden använt några av följande mediciner?** Blodtrycksmedicin  1 = ja  Hjärt/kärlkrampsmedicin (endast VIP + MONICA 1986)  1 = ja  2 = nej (endast MONICA 1986)  Lugnande medel eller sömnmedel (endast VIP + MONICA 1986)  1 = ja  2 = nej (endast MONICA 1986)  Magsårs/magkatarrmedicin** (endast VIP)  1 = ja Blodfettssänkande** 1 = ja (i VIP finns endast alt 1)  2 = nej  3 = Vet ej Nej, använder inga av ovanstående mediciner (endast VIP) 1 = ja Smärtstillande medel** (endast VIP + MONICA 1986) 1 = ja  2 = nej (endast MONICA 1986)  Acetylsalicylsyra mot hjärtkärlsjukdom (endast MONICA)  1 = ja  2 = För annan sjd  3 = nej  Acetylsalicylsyra mot slaganfall (endast MONICA)  1 = ja  2 = För annan sjd  3 = nej  Har du under den senaste 14-dagarsperioden använt andra mediciner som skrivits ut av läkare som t ex medel mot depression, epilepsi, penicillin eller hormoner, eller någon annan medicin som ej skrivits ut av läkare som t ex magnecyl, vitamin- eller järnpreparat, omega-3, eller annat kosttillskott, naturmedicin eller annat?**  1 = ja  2 = nej |
|  | **med1-med29**  VIP: 1985 ->  MONICA: 1986-2014 | **ATC-koder** Observera att ATC-kod inte är digitaliserad för samtliga tillfällen. Kan begäras studievis efter diskussion med EBF. |
|  | **diabet**  VIP: 1986 ->  MONICA: 1986-2014 | **Har du diabetes?** 1 = Ja  2 = Nej  3 = Vet ej (endast MONICA 2014) |
|  | **diabetesbehandling****  VIP: 1988 ->  MONICA: 1986-2014 | **Behandlas du med…** diabetesbehandling_a = Enbart diet och motion  diabetesbehandling_b = Tabletter  diabetesbehandling_c = Insulin  diabetesbehandling_d = Ingen behandling med ovanstående |
|  | **graviditetsdiabetes**  VIP: 2011 ->  MONICA: 1999-2014 | **Har du haft graviditetsdiabetes?** 1 = Ja  2 = Nej  3 = Har inga barn (endast MONICA)  4 = Vet ej (endast MONICA) |
|  | **infarkt_sjukhus****  VIP: 1989 ->  MONICA: 1986-2014 | ***Har du legat på sjukhus för säker hjärtinfarkt?***  1 = Ja  2 = Nej  3 = Vet ej om säker (endast MONICA) |
|  | **infarkt_sjukhus_ar****  VIP: 1991 ->  MONICA: 1990-2014 | ***Om du legat på sjukhus för säker hjärtinfarkt, vilket år?*** |
|  | **RAND36**  RAND36 levereras som uträknat index och baserar sig på variablerna SF36_RAND_1-SF36_RAND_11d.  Gäller endast personer som deltagit i VIP  Vid önskemål om enskilda SF36_RAND-variabler vänligen kryssa för de som önskas nedan. | **RAND-36 – Mäter olika aspekt av hälsa baserat på 36 hälsofrågor.** Frågorna sammanfattas i 8 delskalor:  PF – Physical functioning (Fysisk funktionsförmåga)  RP – Role limitations due to physical health (Begränsningar p.g.a. fysisk hälsa)  BP – Role limitations due to emotional problems (Begränsningar på grund av emotionella problem)  GH – Energy/Fatigue (Energi/Utmattning)  VT – Emotional well-being (Psykiskt välmående)  SF – Social functioning (Social funktionsförmåga)  RE – Pain (Smärta)  MH – General health (Allmän Hälsa)  Skalorna får ett värde mellan 0 och 100.  RAND-36 mäts av samma frågor som i 36-item Short Form Survey (SF-36) och uppvisar ingen större skillnad mot den. SF-36 kräver dock licens och vid avsikt att beräkna SF-36 får sökande beställa de enskilda frågorna separat och beräkna med egen licens. |
|  | **SF36_RAND_1**  VIP: 2003 ->  Gäller endast personer som deltagit i VIP | ***I allmänhet, skulle du säga att din hälsa är:***  1 = Utmärkt  2 = Mycket god  3 = God  4 = Någorlunda  5 = Dålig |
|  | **SF36_RAND _2**  VIP: 2003 ->  Gäller endast personer som deltagit i VIP | ***Jämfört med för ett år sedan, hur skulle du vilja bedöma ditt allmänna hälsotillstånd nu?***  1 = Mycket bättre nu än för ett år sedan  2 = Något bättre nu än för ett år sedan  3 = Ungefär detsamma  4 = Något sämre nu än för ett år sedan  5 = Mycket sämre nu än för ett år sedan |
|  | **SF36_RAND _3a**  VIP: 2003 ->  Gäller endast personer som deltagit i VIP | ***Är du pga ditt kroppsliga hälsotillstånd begränsad vid ansträngande aktiviteter som att springa, lyfta tunga saker, delta i ansträngande sporter?***  1 = Ja, mycket begränsad  2 = Ja, lite begränsad  3 = Nej, inte alls begränsad |
|  | **SF36_RAND _3b**  VIP: 2003 ->  Gäller endast personer som deltagit i VIP | ***Är du pga ditt kroppsliga hälsotillstånd begränsad vid måttligt ansträngande aktiviteter, som att flytta ett bord, dammsuga, skogspromenader eller trädgårdsarbete?***  1 = Ja, mycket begränsad  2 = Ja, lite begränsad  3 = Nej, inte alls begränsad |
|  | **SF36_RAND _3c**  VIP: 2003 ->  Gäller endast personer som deltagit i VIP | ***Är du pga ditt kroppsliga hälsotillstånd begränsad vid måttligt ansträngande aktiviteter, som att lyfta eller bära matkassar?***  1 = Ja, mycket begränsad  2 = Ja, lite begränsad  3 = Nej, inte alls begränsad |
|  | **SF36_RAND _3d**  VIP: 2003 ->  Gäller endast personer som deltagit i VIP | ***Är du pga ditt kroppsliga hälsotillstånd begränsad vid måttligt ansträngande aktiviteter, som att gå uppför flera trappor?***  1 = Ja, mycket begränsad  2 = Ja, lite begränsad  3 = Nej, inte alls begränsad |
|  | **SF36_RAND _3e**  VIP: 2003 ->  Gäller endast personer som deltagit i VIP | ***Är du pga ditt kroppsliga hälsotillstånd begränsad vid måttligt ansträngande aktiviteter, som att gå uppför en trappa?***  1 = Ja, mycket begränsad  2 = Ja, lite begränsad  3 = Nej, inte alls begränsad |
|  | **SF36_RAND _3f**  VIP: 2003 ->  Gäller endast personer som deltagit i VIP | ***Är du pga ditt kroppsliga hälsotillstånd begränsad vid måttligt ansträngande aktiviteter, som att böja dig eller gå ner på knä?***  1 = Ja, mycket begränsad  2 = Ja, lite begränsad  3 = Nej, inte alls begränsad |
|  | **SF36_RAND _3g**  VIP: 2003 ->  Gäller endast personer som deltagit i VIP | ***Är du pga ditt kroppsliga hälsotillstånd begränsad vid måttligt ansträngande aktiviteter, som att gå mer än 2 kilometer?***  1 = Ja, mycket begränsad  2 = Ja, lite begränsad  3 = Nej, inte alls begränsad |
|  | **SF36_RAND _3h**  VIP: 2003 ->  Gäller endast personer som deltagit i VIP | ***Är du pga ditt kroppsliga hälsotillstånd begränsad vid måttligt ansträngande aktiviteter, som att gå några hundra meter?***  1 = Ja, mycket begränsad  2 = Ja, lite begränsad  3 = Nej, inte alls begränsad |
|  | **SF36_RAND _3i**  VIP: 2003 ->  Gäller endast personer som deltagit i VIP | ***Är du pga ditt kroppsliga hälsotillstånd begränsad vid måttligt ansträngande aktiviteter, som att gå hundra meter?***  1 = Ja, mycket begränsad  2 = Ja, lite begränsad  3 = Nej, inte alls begränsad |
|  | **SF36_RAND _3j**  VIP: 2003 ->  Gäller endast personer som deltagit i VIP | ***Är du pga ditt kroppsliga hälsotillstånd begränsad vid måttligt ansträngande aktiviteter, som att bada eller klä på dig?***  1 = Ja, mycket begränsad  2 = Ja, lite begränsad  3 = Nej, inte alls begränsad |
|  | **SF36_RAND _4a**  VIP: 2003 ->  Gäller endast personer som deltagit i VIP | ***Under de senaste fyra veckorna, har du som följd av ditt kroppsliga hälsotillstånd skurit ned den tid du normalt ägnar åt arbete eller andra aktiviteter?***  1 = Ja  2 = Nej |
|  | **SF36_RAND _4b**  VIP: 2003 ->  Gäller endast personer som deltagit i VIP | ***Under de senaste fyra veckorna, har du som följd av ditt kroppsliga hälsotillstånd uträttat mindre än du skulle önska?***  1 = Ja  2 = Nej |
|  | **SF36_RAND _4c**  VIP: 2003 ->  Gäller endast personer som deltagit i VIP | ***Under de senaste fyra veckorna, har du som följd av ditt kroppsliga hälsotillstånd varit hindrad att utföra vissa arbetsuppgifter eller andra aktiviteter?***  1 = Ja  2 = Nej |
|  | **SF36_RAND _4d**  VIP: 2003 ->  Gäller endast personer som deltagit i VIP | ***Under de senaste fyra veckorna, har du som följd av ditt kroppsliga hälsotillstånd haft svårigheter att utföra ditt arbete eller andra aktiviteter?***  1 = Ja  2 = Nej |
|  | **SF36_RAND _5a**  VIP: 2003 ->  Gäller endast personer som deltagit i VIP | ***Under de senaste fyra veckorna, har du som följd av känslomässiga problem skurit ner den tid du normalt ägnat åt arbete eller andra aktiviteter?***  1 = Ja  2 = Nej |
|  | **SF36_RAND _5b**  VIP: 2003 ->  Gäller endast personer som deltagit i VIP | ***Under de senaste fyra veckorna, har du som följd av känslomässiga problem uträttat mindre än du skulle önskat?***  1 = Ja  2 = Nej |
|  | **SF36_RAND _5c**  VIP: 2003 ->  Gäller endast personer som deltagit i VIP | ***Under de senaste fyra veckorna, har du som följd av känslomässiga problem inte utfört arbete eller andra aktiviteter så noggrant som vanligt?***  1 = Ja  2 = Nej |
|  | **SF36_RAND _6**  VIP: 2003 ->  Gäller endast personer som deltagit i VIP | ***Under de senaste fyra veckorna, i vilken utsträckning har dit kroppsliga hälsotillstånd eller dina känslomässiga problem stört ditt vanliga umgänge med anhöriga, vänner, grannar eller andra?***  1 = Inte alls  2 = Lite  3 = Måttligt  4 = Mycket  5 = Väldigt mycket |
|  | **SF36_RAND _7**  VIP: 2003 ->  Gäller endast personer som deltagit i VIP | ***Hur mycket värk eller smärta har du haft under de senaste fyra veckorna?***  1 = Ingen  2 = Mycket lätt  3 = Lätt  4 = Måttlig  5 = Svår  6 = Mycket svår |
|  | **SF36_RAND _8**  VIP: 2003 ->  Gäller endast personer som deltagit i VIP | ***Under de senaste fyra veckorna, hur mycket har värken eller smärtan stört ditt normala arbete?***  1 = Inte alls  2 = Lite  3 = Måttligt  4 = Mycket  5 = Väldigt mycket |
|  | **SF36_RAND _9a**  VIP: 2003 ->  Gäller endast personer som deltagit i VIP | ***Hur stor del av tiden de senaste fyra veckorna har du känt dig riktigt pigg och stark?***  1 = Hela tiden  2 = Största delen av tiden  3 = En hel del av tiden  4 = En del av tiden  5 = Lite av tiden  6 = Inget av tiden |
|  | **SF36_RAND _9b**  VIP: 2003 ->  Gäller endast personer som deltagit i VIP | ***Hur stor del av tiden de senaste fyra veckorna har du känt dig mycket nervös?***  1 = Hela tiden  2 = Största delen av tiden  3 = En hel del av tiden  4 = En del av tiden  5 = Lite av tiden  6 = Inget av tiden |
|  | **SF36_RAND _9c**  VIP: 2003 ->  Gäller endast personer som deltagit i VIP | ***Hur stor del av tiden de senaste fyra veckorna har du känt dig så nedstämd att inget kunnat muntra upp dig?***  1 = Hela tiden  2 = Största delen av tiden  3 = En hel del av tiden  4 = En del av tiden  5 = Lite av tiden  6 = Inget av tiden |
|  | **SF36_RAND _9d**  VIP: 2003 ->  Gäller endast personer som deltagit i VIP | ***Hur stor del av tiden de senaste fyra veckorna har du känt dig lugn och harmonisk?***  1 = Hela tiden  2 = Största delen av tiden  3 = En hel del av tiden  4 = En del av tiden  5 = Lite av tiden  6 = Inget av tiden |
|  | **SF36_RAND _9e**  VIP: 2003 ->  Gäller endast personer som deltagit i VIP | ***Hur stor del av tiden de senaste fyra veckorna har du varit full av energi?***  1 = Hela tiden  2 = Största delen av tiden  3 = En hel del av tiden  4 = En del av tiden  5 = Lite av tiden  6 = Inget av tiden |
|  | **SF36_RAND _9f**  VIP: 2003 ->  Gäller endast personer som deltagit i VIP | ***Hur stor del av tiden de senaste fyra veckorna har du känt dig dyster och ledsen?***  1 = Hela tiden  2 = Största delen av tiden  3 = En hel del av tiden  4 = En del av tiden  5 = Lite av tiden  6 = Inget av tiden |
|  | **SF36_RAND _9g**  VIP: 2003 ->  Gäller endast personer som deltagit i VIP | ***Hur stor del av tiden de senaste fyra veckorna har du känt dig utsliten?***  1 = Hela tiden  2 = Största delen av tiden  3 = En hel del av tiden  4 = En del av tiden  5 = Lite av tiden  6 = Inget av tiden |
|  | **SF36_RAND _9h**  VIP: 2003 ->  Gäller endast personer som deltagit i VIP | ***Hur stor del av tiden de senaste fyra veckorna har du känt dig glad och lycklig?***  1 = Hela tiden  2 = Största delen av tiden  3 = En hel del av tiden  4 = En del av tiden  5 = Lite av tiden  6 = Inget av tiden |
|  | **SF36_RAND _9i**  VIP: 2003 ->  Gäller endast personer som deltagit i VIP | ***Hur stor del av tiden de senaste fyra veckorna har du känt dig trött?***  1 = Hela tiden  2 = Största delen av tiden  3 = En hel del av tiden  4 = En del av tiden  5 = Lite av tiden  6 = Inget av tiden |
|  | **SF36_RAND _10**  VIP: 2003 ->  Gäller endast personer som deltagit i VIP | ***Under de senaste fyra veckorna, hur stor del av tiden har ditt kroppsliga hälsotillstånd eller dina känslomässiga problem stört dina möjligheter att umgås (t ex hälsa på släkt, vänner, etc.)?***  1 = Hela tiden  2 = Största delen av tiden  3 = En del av tiden  4 = Lite av tiden  5 = Inget av tiden |
|  | **SF36_RAND _11a**  VIP: 2003 ->  Gäller endast personer som deltagit i VIP | ***Jag verkar ha lite lättare att bli sjuk än andra människor***  1 = Stämmer precis  2 = Stämmer ganska bra  3 = Osäker  4 = Stämmer inte särskilt bra  5 = Stämmer inte alls |
|  | **SF36_RAND _11b**  VIP: 2003 ->  Gäller endast personer som deltagit i VIP | ***Jag är lika frisk som vem som helst av dem jag känner***  1 = Stämmer precis  2 = Stämmer ganska bra  3 = Osäker  4 = Stämmer inte särskilt bra  5 = Stämmer inte alls |
|  | **SF36_RAND _11c**  VIP: 2003 ->  Gäller endast personer som deltagit i VIP | ***Jag tror att min hälsa kommer att bli sämre***  1 = Stämmer precis  2 = Stämmer ganska bra  3 = Osäker  4 = Stämmer inte särskilt bra  5 = Stämmer inte alls |
|  | **SF36_RAND _11d**  VIP: 2003 ->  Gäller endast personer som deltagit i VIP | ***Min hälsa är utmärkt***  1 = Stämmer precis  2 = Stämmer ganska bra  3 = Osäker  4 = Stämmer inte särskilt bra  5 = Stämmer inte alls |
|  | **livskvalitet****  VIP: 1996->  MONICA: 1999-2004 | **Markera hur tillfredsställd du är med din situation i olika avseenden.**  Mycket dålig=1 . . . . alldeles utmärkt=7  livskvalitet_d1 = Hem och familjesituation  livskvalitet_d2 = Bostad  livskvalitet_d3 = Arbetssituation  livskvalitet_d4 = Ekonomi  livskvalitet_d5 = Fritid  Man kan uppleva en del förändringar inom sig själv under  årens lopp, försök att nedan markera hur du känner dig nu.  Mycket dålig=1 . . . . alldeles utmärkt=7  livskvalitet_d6 = Hörseln  livskvalitet_d7 = Synen  livskvalitet_d8 = Minnet  livskvalitet_d9 = Konditionen  livskvalitet_d10 = Aptiten  livskvalitet_d11 = Humöret  livskvalitet_d12 = Energin  livskvalitet_d13 = Tålamodet  livskvalitet_d14 = Självförtroendet livskvalitet_d15 = Sömnen  Känner du dig betydelsefull och uppskattad  Mycket dålig=1 . . . . alldeles utmärkt=7  livskvalitet_d16 = utanför hemmet?  livskvalitet_d17 = i hemmet? |
|  | **ISSI**  ISSI levereras som uträknat index och baserar sig på variablerna socont-sochelp.  Gäller endast personer som deltagit i VIP  Vid önskemål om enskilda ”soc”-variabler vänligen kryssa för de som önskas nedan. | **Interview Schedule for Social Interaction (ISSI)** är en serie frågor om ens sociala nätverk som kan räknas samman till skalorna AVSI – AVailability of Social Interaction (Sociala nätverkets storlek och funktionalitet), och AVAT - AVailability of ATtachment (Tillgång till närhet och emotionellt stöd). |
|  | **sockont****  VIP: 1985->  MONICA: 1986-2009 | **Hur många människor känner du och har kontakt med som har samma intressen som du?**  1 = Ingen  2 = 1-2 personer  3 = 3-5 personer  4 = 6-10 personer  5 = 11-15 personer  6 = > 15 personer |
|  | **socsam****  VIP: 1985->  MONICA: 1986-2009 | ***Hur många människor, som du känner, träffar eller samtalar du med under en vanlig vecka?***  1 = Ingen  2 = 1-2 personer  3 = 3-5 personer  4 = 6-10 personer  5 = 11-15 personer  6 = mer än 15 personer |
|  | **soclago****  VIP: 1985->  MONICA: 1986-2009 | **Är det ungefär lagom många människor du träffar i ditt dagliga liv? Skulle du vilja träffa fler eller färre människor?**  1 = Färre  2 = Lagom många  3 = Fler |
|  | **sochem****  VIP: 1985->  MONICA: 1986-2009 | ***Hur många vänner har du som kan komma hem till dig när som helst och känna sig hemma?***  (Du skulle inte bry dig om, om det var ostädat eller om du höll på att äta. Nära släktingar skall inte räknas med.)  1 = Ingen  2 = 1-2 personer  3 = 3-5 personer  4 = 6-10 personer  5 = 11-15 personer  6 = mer än 15 personer |
|  | **soctala****  VIP: 1985->  MONICA: 1986-2009 | **Hur många finns det som du kan tala öppet med utan att tänka dig för?**  1 = Ingen  2 = 1-2 personer  3 = 3-5 personer  4 = 6-10 personer  5 = 11-15 personer  6 = > 15 personer |
|  | **socstod****  VIP: 1985->  MONICA: 1986-2009 | **Finns någon särskild person att verkligen få stöd från?** 1 = Ja  2 = Ja, men jag behöver inte  3 = Nej |
|  | **socnara****  VIP: 1985->  MONICA: 1986-2009 | ***Finns det någon särskild person som känner sig stå väldigt nära dig?***  1 = Ja  2 = Är inte säker  3 = Nej |
|  | **soclyck****  VIP: 1985->  MONICA: 1986-2009 | ***Har du någon särskild person som du kan dela dina innersta känslor med när du känner dig lycklig? Någon som själv känner sig lycklig bara för att du är det?***  1 = Ja  2 = Nej |
|  | **socanfo****  VIP: 1985->  MONICA: 1986-2009 | **Har du någon att dela dina innersta känslor med och anförtro dig åt?**  1 = Ja  2 = Nej |
|  | **soctrost****  VIP: 1985->  MONICA: 1986-2009 | ***Händer det att någon håller om dig till tröst och stöd?***  1 = Ja  2 = Nej |
|  | **socupps****  VIP: 1985->  MONICA: 1986-2009 | **Tror du att de därhemma eller andra uppskattar vad du gör?**  1 = Ja  2 = Inte tillräckligt  3 = Nej, inte alls |
|  | **soclana****  VIP: 1985->  MONICA: 1986-2009 | ***Finns det människor i din omgivning som du lätt kan be om saker, t.ex. människor som du känner så väl att du kan låna verktyg eller köksgrejor?***  1 = Ja  2 = Nej |
|  | **sochelp****  VIP: 1985->  MONICA: 1986-2009 | **Bortsett från de därhemma kan du vända dig till någon när du är i svårigheter?**  1 = Ja  2 = Nej |
|  | **socdelta****  VIP: 1989->  MONICA: 1999-2009 | ***Har du under det senaste året deltagit i någon föreningsverksamhet, frivilligorganisation etc tillsammans med andra(t.ex. sport, studiecirkel, teatergrupp, sångkör, politisk förening)?***  1 = Ja  2 = Nej |
|  | **socofta**  VIP: 1985->  MONICA: 1986-2009 | ***Hur ofta ägnar du dig åt föreningsaktivitet, klubbverksamhet, studiecirkel etc. tillsammans med andra?***  1 = 1-2 gånger per år  2 = 1-2 gånger per månad  3 = 1-2 gånger per vecka  4 = varje dag  5 = vet ej |
|  | **socforening**  VIP: 2001->  MONICA: 2004-2009 | ***Vilken/vilka föreningsaktiviteter deltar du i?***  socforening_a = Sport & motion  socforening_b = Studiecirkel  socforening_c = Teatergrupp  socforening_d = Sångkör  socforening_e = Annan förening |
|  | **Karasek**  Karasek levereras som uträknat index och baserar sig på variablerna arbfys-arbvad.  Gäller endast personer som deltagit i VIP  Vid önskemål om enskilda ”arb”-variabler vänligen kryssa för de som önskas nedan. | **Karasek beräknas från en serie frågor om arbetssituation framtagna av Robert Karasek och medarbetare.** Från frågorna poängsätts 2 skalor: KDL – Decision latitude (inflytande) och KWS – Work strain (Krav). Baserat på skalornas värde på individnivå jämfört med resten av studiepopulationen klassificeras arbetssituationen som:  1 = Avspänd  2 = Passiv  3 = Aktiv  4 = Spänd |
|  | **arbfys**  VIP: 1986->  MONICA: 1986-2009 | **Är ditt arbete fysiskt tungt?** 1 = Ja ofta  2 = Ja ibland  3 = Nej sällan  4 = Nej så gott som aldrig |
|  | **arbfort**  VIP: 1985->  MONICA: 1986-2009 | **Kräver ditt arbete att du arbetar mycket fort?** 1 = Ja ofta  2 = Ja ibland  3 = Nej sällan  4 = Nej så gott som aldrig |
|  | **arbpsyk**  VIP: 1986->  MONICA: 1986-2009 | **Är ditt arbete psykiskt påfrestande?** 1 = Ja ofta  2 = Ja ibland  3 = Nej sällan  4 = Nej så gott som aldrig |
|  | **arbhin**  VIP: 1985->  MONICA: 1986-2009 | **Har du tillräckligt med tid för dina arbetsuppgifter?** 1 = Ja ofta  2 = Ja ibland  3 = Nej sällan  4 = Nej så gott som aldrig |
|  | **arbkrav**  VIP: 1985->  MONICA: 1986-2009 | **Förekommer det motstridiga krav i ditt arbete?** 1 = Ja ofta  2 = Ja ibland  3 = Nej sällan  4 = Nej så gott som aldrig |
|  | **arbnytt**  VIP: 1985->  MONICA: 1986-2009 | **Får du lära dig nya saker i ditt arbete?** 1 = Ja ofta  2 = Ja ibland  3 = Nej sällan  4 = Nej så gott som aldrig |
|  | **arbski**  VIP: 1985->  MONICA: 1986-2009 | **Kräver ditt arbete skicklighet?** 1 = Ja ofta  2 = Ja ibland  3 = Nej sällan  4 = Nej så gott som aldrig |
|  | **arbide**  VIP: 1985->  MONICA: 1986-2009 | **Kräver ditt arbete påhittighet?** 1 = Ja ofta  2 = Ja ibland  3 = Nej sällan  4 = Nej så gott som aldrig |
|  | **arbrut**  VIP: 1985->  MONICA: 1986-2009 | **Innebär ditt arbete att man gör samma saker om och om igen?** 1 = Ja ofta  2 = Ja ibland  3 = Nej sällan  4 = Nej så gott som aldrig |
|  | **arbhur**  VIP: 1985->  MONICA: 1986-2009 | **Frihet att bestämma hur arbetet ska utföras?** 1 = Ja ofta  2 = Ja ibland  3 = Nej sällan  4 = Nej så gott som aldrig |
|  | **arbvad**  VIP: 1985->  MONICA: 1986-2009 | **Frihet att bestämma vad som ska utföras?** 1 = Ja ofta  2 = Ja ibland  3 = Nej sällan  4 = Nej så gott som aldrig |
|  | **arbtala****  VIP: 1991->  MONICA: 1990-2009 | ***Har du i vanliga fall möjlighet att tala med dina arbetskamrater under rasten, om du skulle vilja det?***  1 = Ja, alltid  2 = Ja, för det mesta  3 = Nej, jag har inga raster  4 = Nej, jag har inga raster med arbetskamrater |
|  | **arblamna****  VIP: 1991->  MONICA: 1990-2009 | ***Är ditt arbete av den karaktären att du kan lämna det ett tag om du vill tala med en arbetskamrat?***  1 =Ja, för det mesta  2 = Ja, ibland  3 = Bara för brådskande ärenden  4 = Nej, det är helt omöjligt |
|  | **arbkontakt****  VIP: 1991->  MONICA: 1990-2009 | ***Har du, som en del i ditt arbete, en mängd kontakter med dina arbetskamrater?***  1 = Ja, ständigt kontakter i arbetet  2 = En eller ett par ggr/månad  3 = Nej, jag arbetar för det mesta ensam  4 = Sällan eller aldrig |
|  | **arbfritid****  VIP: 1991->  MONICA: 1990-2009 | ***Hur ofta är du vanligen tillsammans med en eller flera av dina arbetskamrater på fritid?***  1 = En eller flera ggr/vecka  2 = En eller ett par ggr/månad  3 = En eller ett par ggr/år  4 = Sällan eller aldrig |
|  | **arbbesok****  VIP: 1991->  MONICA: 1990-2009 | ***När besökte någon av dina arbetskamrater dig senast?***  1 = För en till fyra veckor sedan  2 = För en till tolv månader sedan  3 = För mer än ett år sedan  4 = Har aldrig haft besök av någon arbetskamrat |
|  | **MONICA_motion_fritid_86_09**  MONICA: 1986-2009 Gäller endast personer som deltagit i MONICA | **Hur mycket har du rört dig eller ansträngt dig kroppsligt på din fritid under det senaste året?** 1 = Knappast något alls  2 = Mestadels stillasittande, ibland någon promenad eller liknande  3 = Lättare fysisk ansträngning minst 2 timmar i veckan.  4 = Mer ansträngande motion 1-2 timmar i veckan.  5 = Mer ansträngande motion minst 3 timmar i veckan.  6 = Hård träning eller tävling regelbundet och flera gånger i veckan där den fysiska ansträngningen är stor.  7 = Aldrig (endast 1986)  8 = 1-2 ggr per månad (endast 1986)  9 = 1 gång per vecka (endast 1986)  10 = 2-3 ggr per vecka (endast 1986)  11 = 4 eller fler gånger per vecka (endast 1986) |
|  | **MONICA_motion_arbete**  MONICA: 1990-2009 Gäller endast personer som deltagit i MONICA | **Hur mycket har du rört dig eller ansträngt dig kroppsligt i ditt arbete under det senaste året?** 1 = Har ålders-förtidspension (alternativet finns inte 1990)  2 = Stillasittande arbete  3 = Lätt, men rörligt arbete  4 = Måttligt tungt arbete  5 = Tungt arbete |
|  | **g1_a - g1_d****  VIP: 1989->  MONICA: 2014 | **Markera i tabellen nedan hur Du oftast färdas till och från arbetet för varje årstid** Vår, g1_a 1 = Bil  2 = Buss  3 = Promenerar  4 = Cyklar  Sommar, g1_b  1 = Bil  2 = Buss  3 = Promenerar  4 = Cyklar  Höst, g1_c  1 = Bil  2 = Buss  3 = Promenerar  4 = Cyklar  Vinter, g1_d  1 = Bil  2 = Buss  3 = Promenerar  4 = Cyklar |
|  | **antal_km****  VIP: 1989->  MONICA: 2014 | **Antal km till arbetet** |
|  | **g2_a – g2_e****  VIP: 1989->  MONICA: 2014 | **Markera det alternativ som bäst beskriver ditt arbete** g2_a = Stillasittande eller stående  g2_b = Lätt men delvis rörligt  g2_c = Lätt och rörligt  g2_d = Ibland fysiskt tungt  g2_e = Fysiskt tungt större delen av tiden |
|  | **g3_a****  VIP: 1989->  Gäller endast personer som deltagit i VIP | ***Hur ofta ägnar du dig på din fritid åt promenader?***  0 = Aldrig  1 = 1-2 ggr/månad  2 = 3-4 ggr/månad  3 = 2-3 ggr/vecka  4 = Varje dag |
|  | **g3_b****  VIP: 1989->  Gäller endast personer som deltagit i VIP | ***Hur ofta ägnar du dig på din fritid åt cykelturer?***  0 = Aldrig  1 = 1-2 ggr/månad  2 = 3-4 ggr/månad  3 = 2-3 ggr/vecka  4 = Varje dag |
|  | **g3_c****  VIP: 1989-2005  Gäller endast personer som deltagit i VIP | ***Hur ofta ägnar du dig på din fritid åt dans/folkdans?***  0 = Aldrig  1 = Sällan  2 = Någon gång varje månad  3 = Varje vecka  4 = Varje dag |
|  | **g3_d****  VIP: 1989-2005  Gäller endast personer som deltagit i VIP | ***Hur ofta ägnar du dig på din fritid åt snöskottning?***  0 = Aldrig  1 = Sällan  2 = Någon gång varje månad  3 = Varje vecka  4 = Varje dag |
|  | **g3_e****  VIP: 1989-2005  Gäller endast personer som deltagit i VIP | ***Hur ofta ägnar du dig på din fritid åt trädgårdsarbete?***  0 = Aldrig  1 = Sällan  2 = Någon gång varje månad  3 = Varje vecka  4 = Varje dag |
|  | **g3_f****  VIP: 1989-2005  Gäller endast personer som deltagit i VIP | ***Hur ofta ägnar du dig på din fritid åt jakt/fisketurer?***  0 = Aldrig  1 = Sällan  2 = Någon gång varje månad  3 = Varje vecka  4 = Varje dag |
|  | **g3_g****  VIP: 1989-2005  Gäller endast personer som deltagit i VIP | ***Hur ofta ägnar du dig på din fritid åt bär/svampplockning?***  0 = Aldrig  1 = Sällan  2 = Någon gång varje månad  3 = Varje vecka  4 = Varje dag |
|  | **g4****  VIP: 1989-2005  Gäller endast personer som deltagit i VIP | **Har du ändrat din ”vardagsmotion” under det senaste året?** 1 = Minskat mycket  2 = Minskat något  3 = Som tidigare  4 = Ökat något  5 = Ökat mycket |
|  | **g5****  VIP: 1989-2005  Gäller endast personer som deltagit i VIP | **”Den vardagsmotion jag får tillfredställer mitt behov av att röra på mig”. Stämmer detta påstående in på dig?** 1 = Inte alls  2 = Ganska dåligt  3 = Delvis  4 = Helt och hållet |
|  | **g6****  VIP: 1989->  MONICA: 2014 | **Hur ofta har du tränat eller motionerat i träningskläder de senaste tre månaderna, i syfte att förbättra din kondition och/eller för att må bra?** 1 = Aldrig  2 = Då och då – ej regelbundet  3 = 1-2 ggr/vecka  4 = 2-3 ggr/vecka  5 = Mer än 3 ggr/vecka |
|  | **g7****  VIP: 1989-2005  Gäller endast personer som deltagit i VIP | **Om du motionerar – har du ändrat dina motionsvanor under det senaste året?** 1 = Minskat mycket  2 = Minskat något  3 = Som tidigare  4 = Ökat något  5 = Ökat mycket |
|  | **g8****  VIP: 1989-2005  Gäller endast personer som deltagit i VIP | **Hur fysiskt aktiv var du före 20-års ålder?** 1 = Befriad från skolgymnastiken  2 = Deltog enbart i skolgymnastiken  3 = Tränade utan att tävla  4 = Deltog i både träning och tävling (ej på elitnivå)  5 = Tränade och tävlade på elitnivå  Tvåsiffriga tal eller större anger vilka alternativ som kombinerats. |
|  | **g9**  VIP: 2005->  Gäller endast personer som deltagit i VIP | ***Hur mycket har du ansträngt dig kroppsligt under de senaste***  ***12 månaderna?***  Om din aktivitet varierar mellan t.ex. sommar och vinter, så försök att ta ett genomsnitt.  1 = Stillasittande fritid.  Du ägnar dig mest åt läsning, tv, bio eller annan stillasittandesysselsättning på fritiden. Du promenerar, cyklar rör på dig på annat sätt mindre än två timmar i veckan.  2 = Måttlig motion på fritiden.  Du promenerar, cyklar eller rör dig på annat sätt under minst 2 timmar i veckan oftast utan att svettas. I detta inräknas t.ex. promenad eller cykling till och från arbetet, övriga promenader, tyngre hushållsarbete, ordinärt trädgårdsarbete, fiske, bordtennis, bowling.  3 = Måttligt, regelbunden motion på fritiden.  Du motionerar regelbundet 1-2 gånger per vecka minst 30 minuter per gång med t.ex. löpning, simning, tennis, badminton eller annan aktivitet som gör att du svettas.  4 = Regelbunden motion och träning.  Du ägnar dig åt t.ex. löpning, simning, tennis, badminton, motionsgymnastik eller liknande vid genomsnitt minst tre tillfällen per vecka. Vardera tillfälle varar minst 30 minuter per gång. |
|  | **g10**  VIP: 2005->  Gäller endast personer som deltagit i VIP | ***Hur mycket tid ägnar du en vanlig vecka åt måttligt ansträngande aktiviteter som får dig att bli varm?*** t.ex. promenader i rask takt, trädgårdsarbete, tyngre hushållsarbete, cykling, simning. Det kan variera under året, men försök ta någon slags genomsnitt.  1 = 5 timmar per vecka eller mer  2 = mer än 3 timmar, men mindre än 5 timmar per vecka.  3 = Mellan 1 till 3 timmar per vecka.  4 = Högst 1 timme per vecka.  5 = Inte alls.  6 = Vet inte/kan inte ta ställning. |
|  | **g11a_h – g11b_ej**  VIP: 2011->  Gäller endast personer som deltagit i VIP  Detta är frågor om stillasittande hämtade ur IPAQ (validerade frågor om fysisk aktivitet). | ***Hur lång tid har du per dag under de senaste 7 dagarna tillbringat sittande i samband med arbete, studier och transporter, i hemmet och på din fritid?***  Försök uppskatta hur många timmar i genomsnitt. Exempel på detta är tid vid skrivbordet, hemma hos vänner, att åka bil eller buss, att sitta och äta eller prata att sitta framför datorn, och att se på film eller tv.  Antal timmar vardagar, g11a_h  Antal minuter vardagar, g11a_m  Vet ej vardagar, g11a_ej  Antal timmar helger, g11b_h  Antal minuter helger, g11b_m  Vet ej helger, g11b_ej |
|  | **motion****  VIP: 1988-1991  Gäller äldre enkäter i VIP | **Hur mycket motionerar du på din fritid?** 0 = Praktiskt taget inget alls  1 = Då och då  2 = Regelbundet ungefär en gång i veckan  3 = Regelbundet ungefär två gånger i veckan  4 = Regelbundet ganska kraftigt minst två gånger i veckan |
|  | **motion2****  VIP: 1986-1994  Gäller äldre enkäter i VIP | **Hur ofta motionerar du?** 1 = Aldrig  2 = 1-2 ggr/månad  3 = 1 gång/vecka  4 = 2-3 ggr/vecka  5 = 4 eller fler ggr/vecka |
|  | **sleep_h7a-sleep_h7h**  VIP: 2005->  Gäller endast personer som deltagit i VIP | ***Hur stor risk är det att du skulle slumra till eller somna i följande situationer, till skillnad från att bara känna dig trött?***  Det avser ditt vanliga levnadssätt den senaste tiden. Även om du inte gjort allt detta nyligen, så försök att komma på hur det skulle ha påverkat dig.  sleep_h7a, Sitter och läser.  1 = Ingen  2 = Liten  3 = Måttlig  4 = Stor  sleep_h7b, Tittar på tv. (samma alternativ som ovan)  sleep_h7c, Sitter overksam på allmän plats (t.ex. teater eller ett möte). (samma alternativ som ovan)  sleep_h7d, Som passagerare i en bil i en timme utan paus. (samma alternativ som ovan)  sleep_h7e, Ligger ner och vilar på eftermiddagen om omständigheterna tillåter. (samma alternativ som ovan)  sleep_h7f, Sitter och pratar med någon. (samma alternativ som ovan)  sleep_h7g, Sitter stilla efter att ha ätit lunch(utan alkohol). (samma alternativ som ovan)  sleep_h7h, I en bil som stannat några minuter i trafiken. (samma alternativ som ovan) |
|  | **sleep_h8a**  VIP: 2005->  Gäller endast personer som deltagit i VIP | ***Snarkar du när du sover?***  1 = Ja, alltid  2 = Ja, nästan alltid  3 = Ja, ibland  4 = Nej nästan aldrig  5 = Nej aldrig  6 = Vet ej |
|  | **sleep_h8b**  VIP: 2005->  Gäller endast personer som deltagit i VIP | ***Har din maka/make/sambo märkt att du har andningsuppehåll under sömn?***  1 = Ja, alltid  2 = Ja, nästan alltid  3 = Ja, ibland  4 = Nej nästan aldrig  5 = Nej aldrig  6 = Vet ej |
|  | **Alkoholkonsumtion och alkoholrelaterade problem/effekter**  Det finns två olika validerade formulär (CAGE och AUDIT) för beräkning av index gällande alkoholförbrukning och/eller problematik relaterat till alkoholförbrukning. | |
|  | **CAGE**  Gäller endast personer som deltagit i VIP  Vid önskemål om enskilda i- och j-variabler vänligen kryssa för de som önskas nedan. | **CAGE (Cut down, Annoyance, Guilt, Eye-opener)** beräknas utifrån frågorna i2, i3, i4 och i5 alternativt j12, j11, j7 och j6. CAGE räknas ihop till ett index mellan 0-4 där 0-1 tolkas som ”ej tecken till riskabel alkoholkonsumtion” och 2-4 tolkas som ”möjlig riskabel alkoholkonsumtion/alkoholberoende” |
|  | **AUDIT**  Gäller endast personer som deltagit i VIP  Vid önskemål om enskilda j-variabler vänligen kryssa för de som önskas nedan. | **AUDIT (Alcohol Use Disorders Identification Test)** beräknas utifrån frågorna j1-j10. Svaren på frågorna poängsätts och summeras. Maxpoäng är 40.  *För kvinnor:*  6 poäng eller mer indikerar riskfylld/skadlig alkoholkonsumtion  14 poäng eller mer innebär troligt missbruk/beroende  *För män:*  8 poäng eller mer indikerar riskfylld/skadlig alkoholkonsumtion  16 poäng eller mer innebär troligt missbruk/beroende |
|  | **i1****  VIP: 1988-2005  Gäller endast personer som deltagit i VIP | ***Är du absolutist?*** (Från 1992 har man uppmanats att inte svara på fråga i2-i5 om man svarat Ja på i1. Då vi inte utför någon datatvätt kan det ändå finnas värden på dessa.)  1 = Ja  2 = Nej |
|  | **i2**** (Samma fråga som J12 i enkäten)  VIP: 1989->  Gäller endast personer som deltagit i VIP | ***Har du någonsin tyckt att du borde minska din alkoholkonsumtion?***  1 = Ja  2 = Nej |
|  | **i3**** (Samma fråga som J11 i enkäten)  VIP: 1989->  Gäller endast personer som deltagit i VIP | ***Har andra människor irriterat dig genom att kritisera ditt drickande?***  1 = Ja  2 = Nej |
|  | **i4****  VIP: 1989-2005  Gäller endast personer som deltagit i VIP | ***Har du någonsin känt dig illa till mods eller haft skuldkänslor för ditt sätt att dricka?***  1 = Ja  2 = Nej |
|  | **i5****  VIP: 1989-2005  Gäller endast personer som deltagit i VIP | ***Har du någonsin druckit det första du gör på morgonen för att lugna nerverna eller bota baksmälla (tagit dig en återställare)?***  1 = Ja  2 = Nej |
|  | **j1**  VIP: 2005->  MONICA: 2014 | ***Hur ofta dricker du alkohol?***  1 = Aldrig  2 = 1 gång per månad eller mer sällan  3 = 2-4 ggr i mån  4 = 2-3 ggr i veckan  5 = 4 ggr/vecka eller mer |
|  | **j2**  VIP: 2005->  MONICA: 2014 | ***Hur många glas dricker du en typisk dag när du dricker alkohol?***  Med ett glas menas:  50 cl folköl  33 cl starköl  1 glas rött eller vitt vin  1 litet glas starkvin  4 cl sprit t.ex. whisky  1 = 0-2 glas (för MONICA 1-2 glas)  2 = 3-4 glas  3 = 5-6 glas  4 = 7-9 glas  5 = 10 glas eller mer |
|  | **j3**  VIP: 2005->  MONICA: 2014 | ***Hur ofta dricker du sex sådana glas eller mer vid samma tillfälle?***  1 = Aldrig  2 = Mer sällan än en gång i månaden  3 = Varje månad  4 = Varje vecka  5 = Dagligen eller nästan varje dag |
|  | **j4**  VIP: 2005->  Gäller endast personer som deltagit i VIP | ***Hur ofta under senaste året har du inte kunnat sluta dricka sedan du börjat?***  1 = Aldrig  2 = Mer sällan än en gång i månaden  3 = Varje månad  4 = Varje vecka  5 = Dagligen eller nästan varje dag |
|  | **j5**  VIP: 2005->  Gäller endast personer som deltagit i VIP | ***Hur ofta under senaste året har du låtit bli att göra något som du borde på grund av att du drack?***  1 = Aldrig  2 = Mer sällan än en gång i månaden  3 = Varje månad  4 = Varje vecka  5 = Dagligen eller nästan varje dag |
|  | **j6**  VIP: 2005->  Gäller endast personer som deltagit i VIP | ***Hur ofta under senaste året har du behövt en drink på morgonen efter drickande dagen innan (tagit en återställare)?***  1 = Aldrig  2 = Mer sällan än en gång i månaden  3 = Varje månad  4 = Varje vecka  5 = Dagligen eller nästan varje dag |
|  | **j7**  VIP: 2005->  Gäller endast personer som deltagit i VIP | ***Hur ofta under senaste året har du haft skuldkänslor eller samvetsförebråelser på grund av ditt drickande?***  1 = Aldrig  2 = Mer sällan än en gång i månaden  3 = Varje månad  4 = Varje vecka  5 = Dagligen eller nästan varje dag |
|  | **j8**  VIP: 2005->  Gäller endast personer som deltagit i VIP | ***Hur ofta under senaste året har du druckit så att du dagen efter inte kommit ihåg vad du sagt eller gjort?***  1 = Aldrig  2 = Mer sällan än en gång i månaden  3 = Varje månad  4 = Varje vecka  5 = Dagligen eller nästan varje dag |
|  | **j9**  VIP: 2005->  Gäller endast personer som deltagit i VIP | ***Har du eller någon annan blivit skadad på grund av ditt drickande?***  1 = Nej  2 = Ja, men inte under det senaste året  3 = Ja, under det senaste året |
|  | **j10**  VIP: 2005->  Gäller endast personer som deltagit i VIP | ***Har en släkting eller vän, en läkare (eller någon annan inom sjukvården) oroat sig över ditt drickande eller antytt att du borde minska på det?***  1 = Nej  2 = Ja, men inte under det senaste året  3 = Ja, under det senaste året |
|  | **Alkohol och dryckesvanor** | |
|  | I kostdatabasen (NSDD) finns fler variabler som rör alkohol, bla kvalitetskontrollerade frekvenser av alkoholintag. De flesta forskare väljer att använda bearbetade kostdata som rensats för orealistiska värden och relaterats till bla energiintag.  Är du intresserad av:  Bearbetade kostdata gällande alkohol. Tex typ av alkoholhaltig dryck, gram alkohol per dag osv. Ett uttag ur kostdatabasen behöver då göras, se <http://www.biobank.umu.se/for-forskare/biobanksforskning---uttag-av-prover-och-data/biobanksforskning---kostdatabasen/> | |
|  | **Kostdata** | |
|  | Är du intresserad av:  Bearbetade kostdata. Tex dagsportion av olika livsmedel, gram/dag av olika livsmedel, näringsvärdesberäkningar osv. Ett uttag ur kostdatabasen (NSDD) behöver då göras, se <http://www.biobank.umu.se/for-forskare/biobanksforskning---uttag-av-prover-och-data/biobanksforskning---kostdatabasen/> | |
| Rökdata *Vissa antaganden har gjorts för att korrigera för svårigheter i att tolka rådata på grund av enkäternas utformning.* | | |
|  | **sm_status**  VIP: 1985->  MONICA: 1986-2014 | ***Rökstatus:***  1 = Rökare  2 = Ex rökare  3 = Icke rökare  4 = Röker då och då  5 = Rökte tidigare då och då |
|  | **sm_cig_groups****  VIP: 1992->  Gäller endast personer som deltagit i VIP | ***Antal cigaretter per dag:***  1= 1-4  2= 5 -14  3= 15 - 24  4= >25 |
|  | **sm_num_cig**  VIP: 1985-1992  MONICA: 1986-2014 | ***Antal cigaretter per dag*** |
|  | **sm_num_cigar**  VIP: 1985-1992  MONICA: 1986-2009 | ***Antal cigarrer per dag***  *(pga av att man i en del enkättyper efterfrågat cigarrer per vecka har antalet i vissa fall dividerats med 7)* |
|  | **sm_gr_tobacco**  VIP: 1985-1992  MONICA: 1986-2014 | ***Gram tobak per vecka*** |
|  | **sm_how_often**  VIP: 2011->  MONICA: 1994-2014 | ***Hur ofta röker du?***  *(Är en följdfråga till de som svarat "Ja, jag röker då och då ej dagligen")*  1 = Mindre än 1 dag/månad  2 = 1 till 3 dagar/månad  3 = Vanligtvis 1 dag/vecka  4 = Vanligtvis 2 till 4 dagar/vecka  5 = Nästan varje dag  *Notering: I MONICA finns endast alternativ 3-5.* |
|  | **sm_start**  VIP: 1985->  MONICA: 1986-2014 | ***Ålder vid rökstart*** |
|  | **sm_stop**  VIP: 1985->  MONICA: 1986-2014 | ***Ålder vid rökslut*** |
|  | **sm_duration**  VIP: 1985->  MONICA: 1986-2014 | ***Rökt i antal år beräknad utifrån sm_start och sm_stop.***  För de som uppgett att de röker och värde för sm_stop saknas anges sm_duration fram tom datum för enkäten. Observera att informationen i sm_duration kan vara missvisande för gruppen rökare som angett att de någon gång slutat röka (och sedan börjat igen). |
|  | **sm_whystop_1 ****  VIP: 1988-1992  MONICA: 1990-2009 | ***Slutade röka av hälsoskäl, på eget initiativ***  1 = Ja |
|  | **sm_whystop_2****  VIP: 1988-1992  MONICA: 1990-2009 | ***Slutade röka på inrådan av läkare/sjukvårdspersonal***  1 = Ja |
|  | **sm_whystop_3 ****  VIP: 1988-1992  MONICA: 1990-2009 | ***Slutade röka pga. annan information/upplysning***  1 = Ja |
|  | **sm_whystop_4 ****  VIP: 1988-1992  MONICA: 1990-2009 | ***Slutade röka pga. tryck från kamrater/familjemedlemmar***  1 = Ja |
|  | **sm_whystop_5 ****  VIP: 1988-1992  MONICA: 1990-2009 | ***Slutade röka av andra skäl***  1 = Ja |
|  | **sn_status**  VIP: 1985->  MONICA: 1986-2014 | ***Snusstatus:***  1 = Snusare  2 = Ex snusare  3 = Icke snusare |
|  | sn_quantity  VIP: 1985->  MONICA: 1986-2014 | ***Snusar antal snusdosor per vecka***  1 = Mindre än 2  2 = 2 till 4  3 = Mer än 4 men mindre än 7  4 = 7 eller mer |
|  | **sn_time**  VIP: 1988->  MONICA: 1990-2014 | ***Snusat antal år*** |
|  | **sn_stopsmoke_a****  VIP: 1991->  MONICA: 1990-2014 | ***Började snusa i samband med att du slutade röka:***  1 = Ja  2 = Nej  3 = Både röker och snusar |
|  | **sn_stopsmoke_b ****  VIP: 1988-1992  Gäller endast personer som deltagit i VIP | ***Började snusa i samband med att du slutade röka:***  1 = Ja  2 = Nej |
|  | **sn_nicotine_replace****  VIP: 2006->  Gäller endast personer som deltagit i VIP | **Använde du nikotinersättningsmedel för att bli snusfri?** 1 = Ja  2 = Nej |
|  | **sm_nicotine_replace****  VIP: 2006->  Gäller endast personer som deltagit i VIP | **Använde du nikotinersättningsmedel för att bli rökfri?** 1 = Ja  2 = Nej |
|  | **nicotine_replace****  VIP: 2006->  Gäller endast personer som deltagit i VIP | **Använder du fortfarande läkemedel mot nikotinberoende trots att du varken röker eller snusar längre?** 1 = Ja  2 = Nej |
|  | **nicotine_94_09**  MONICA: 1994-2009  Gäller endast personer som deltagit i MONICA | **Använder du dagligen andra nikotinprodukter?** 1 = Ja, tuggtobak  2 = Ja, nikotinersättning (nikotintuggummi, -plåster, -nässpray)  3 = Annat  4 = Nej |
|  | **sm_yes_no**  VIP: 1985-2013  Gäller endast personer som deltagit i VIP | **Rökning (gäller de som inte har rökdata från VIP-enkät)**0 = Icke-rökare/inget svar 1 = Rökare |
|  | **sn_yes_no**  VIP: 1985-2013  Gäller endast personer som deltagit i VIP | **Snusning (gäller de som inte har rökdata från VIP-enkät)**0 = Icke-snusare/inget svar 1 = Snusare |

| Cambridge index för fysisk aktivitet***(Detta index är begränsat till fysisk aktivitet vid träningspass och i arbetssituationen. Det tar inte hänsyn till frågor om vardagsmotion, för dessa, se övriga G-frågor.) | |
| --- | --- |
| **pa_index**  VIP: 1989->  Gäller endast personer som deltagit i VIP | **Index för fysisk aktivitet**  1 = Inaktiv  2 = Måttligt inaktiv  3 = Måttligt aktiv  4 = Aktiv  För deltagare där värde saknas i en av de två inkluderade variablerna ersätts saknat värde med den lägsta intensitetsnivån för den variabeln. |
| **pa_index_miss**  VIP: 1989->  Gäller endast personer som deltagit i VIP | **Index för fysisk aktivitet**  1 = Inaktiv  2 = Måttligt inaktiv  3 = Måttligt aktiv  4 = Aktiv  Deltagare där värde saknas i en av de två inkluderade variablerna inkluderas inte i beräkningen. |
| **occup_pa_miss**  VIP: 1989->  Gäller endast personer som deltagit i VIP | 1 = Värde saknas för fysisk aktivitet under arbetstid |
| **leisure_pa_miss**  VIP:1989->  Gäller endast personer som deltagit i VIP | 1 = Värde saknas för fysisk aktivitet på fritiden |

*** Cambridge index för fysisk aktivitet är ett validerat index baserat på två frågor i VIP-enkäten relaterade till fysisk aktivitet på arbetet (G2) och på fritiden (G6). (Interact Consortium "Validity of a short questionnaire to assess physical activity in 10 European countries". Eur J Epidemiol. 2012 Jan;27(1):15-25)
